# Linear Epitope Binding Patterns of Grass Pollen-Specific Antibodies in Allergy and in Response to Allergen-Specific Immunotherapy

**DOI:** 10.3389/falgy.2022.859126

**Published:** 2022-03-31

**Authors:** Linnea Thörnqvist, Ronald Sjöberg, Lennart Greiff, Marianne van Hage, Mats Ohlin

**Affiliations:** ^1^Department of Immunotechnology, Lund University, Lund, Sweden; ^2^Autoimmunity and Serology Profiling, Division of Affinity Proteomics, Department of Protein Science, KTH Royal Institute of Technology, SciLifeLab, Stockholm, Sweden; ^3^Department of Otorhinolaryngology, Head and Neck Surgery, Skåne University Hospital, Lund, Sweden; ^4^Department of Clinical Sciences, Lund University, Lund, Sweden; ^5^Division of Immunology and Allergy, Department of Medicine Solna, Karolinska Institutet and Karolinska University Hospital, Stockholm, Sweden

**Keywords:** allergen, allergen-specific immunotherapy, antibody, grass pollen, linear epitope, peptide microarray

## Abstract

Allergic diseases affect many individuals world-wide and are dependent on the interaction between allergens and antibodies of the IgE isotype. Allergen-specific immunotherapy (AIT) can alter the development of the disease, e.g., through induction of allergen-specific IgG that block allergen-IgE interactions. The knowledge of epitopes recognized by allergy-causing and protective antibodies are limited. Therefore, we developed an allergome-wide peptide microarray, aiming to track linear epitope binding patterns in allergic diseases and during AIT. Here, we focused on immune responses to grass pollen allergens and found that such epitopes were commonly recognized before initiation of AIT and that AIT commonly resulted in increased antibody production against additional epitopes already after 1 year of treatment. The linear epitope binding patterns were highly individual, both for subjects subjected to and for individuals not subjected to AIT. Still, antibodies against some linear epitopes were commonly developed during AIT. For example, the two rigid domains found in grass pollen group 5 allergens have previously been associated to a diversity of discontinuous epitopes. Here, we present evidence that also the flexible linker, connecting these domains, contains regions of linear epitopes against which antibodies are developed during AIT. We also describe some commonly recognized linear epitopes on Phl p 2 and suggest how antibodies against these epitopes may contribute to or prevent allergy in relation to a well-defined stereotyped/public IgE response against the same allergen. Finally, we identify epitopes that induce cross-reactive antibodies, but also antibodies that exclusively bind one of two highly similar variants of a linear epitope. Our findings highlight the complexity of antibody recognition of linear epitopes, with respect to both the studied individuals and the examined allergens. We expect that many of the findings in this study can be generalized also to discontinuous epitopes and that allergen peptide microarrays provide an important tool for enhancing the understanding of allergen-specific antibodies in allergic disease and during AIT.

## Introduction

Antibodies of IgE isotype play a central role in allergic diseases, which affects hundreds of millions of individuals world-wide (1). Through interactions between allergens and the IgE binding receptor FcεRI, allergen-specific IgE can initiate release of inflammatory mediators by effector cells, subsequently leading to downstream symptoms of rhinoconjunctivitis, asthma, or anaphylaxis (2). Most available treatments act by easing symptoms, but allergen-specific immunotherapy (AIT), which is a unique immunological approach, can alter the progression of the disease, with long-term positive effects (3–5). The exact biological mechanisms through which AIT acts still need to be unraveled, but production of protective allergen-specific IgG that block the binding between IgE and allergen is often suggested to be an important factor (6–8). Since AIT can give rise to side-effects (in some cases even anaphylaxis) (9), requires repeated treatment for up to 3 years, and is not always successful, a better understanding of its effects on the immune system is required to facilitate development of safer and more efficient AIT.

Many allergen sources express highly homologous allergens, i.e., so-called isoallergens and variants, which have at least 67% and 90% amino acid sequence identity, respectively (10). Diagnostic measures as well as AIT may be performed using one or several allergen extracts containing multiple different allergens and isoallergens/variants in poorly defined and varying concentrations (11). Recent studies have shown that IgE from allergic donors may bind isoallergens or variants to different extent (12–14), highlighting the importance of taking these variants into account in both research and assessment of clinical outcome.

Defining the epitopes of allergy-causing IgE, and of protective IgG, is one of many important tasks in increasing the understanding of allergic disease and the effects of AIT. However, such analyses are not without challenges. Many antibodies involved in allergy recognize discontinuous epitopes on folded protein allergens [reviewed in (15)], and such epitopes are not yet possible to analyse in a comprehensive, high-throughput/high-content manner. In contrast, linear epitopes are amendable to analysis as they can be represented by short linear peptides and are therefore less tedious to define and track. Indeed, such technology has been used to study linear epitope binding patterns for antibodies against many different allergens (16–22). In some instances, antibodies binding these linear peptides have also been shown to have a potential clinical relevance, as they may act as biomarkers for diagnosis of allergic disease (21, 22). In addition, we reason that this addressable subpopulation of epitopes may give insight into processes of epitope diversity and spread, which also may apply to the important set of discontinuous epitopes.

We have contributed to this work by creating an allergome-wide 16-mer peptide microarray (20). Subsequently, we have used this microarray to analyse allergen recognition of antibodies in serum collected from allergic individuals during treatment with AIT against pollen allergy and identified peptides that represent candidate epitopes within the generated data (20). We have also exploited this data for in-depth analysis of the response to linear epitopes on group 1 grass pollen allergens (a member of the expansin allergen family) and of tree pollen allergen Bet v 1 and its related allergens of the PR-10 family of allergens (20). Now, we extend our study to other major and minor grass pollen allergens represented on the microarray, thereby further expanding the understanding of antibody responses in grass pollen allergy and the effect on these by AIT. For example, we identify antibodies against linear epitopes on the surface of Phl p 2.0101, which may have a potential to act as preventing or cross-linking antibodies in relation to the previously well-described stereotyped/public Phl p 2 specific IgE (23–25). As our microarray platform includes peptides from different isoallergens and variants, we are also able to describe a variation in linear epitopes between highly homologous allergens and provide evidence on how single amino acid differences may differentially impact the binding of antibodies from different allergic individuals.

## Materials and Methods

### Microarray Design and Assay

An allergen peptide microarray had previously been designed, produced, and utilized to determine the peptide binding pattern of IgE, IgG, and IgG4 in serum or plasma from 15 allergic donors (20). Briefly, the microarray contained 16-mer peptides, with generally 15 amino acid overlap, of all allergens that at the time were present in the allergen database generated by the World Health Organization/IUIS Allergen Nomenclature Sub-Committee (http://www.allergen.org, accessed 2014-10-30) and of five additional proteins.

As previously described (26), samples were collected from 15 allergic subjects, of whom subjects 1–8 were subjected to AIT and subjects 9–15 were untreated controls. Donor 2, 3, 4, 7, and 8 were treated with subcutaneous injections with a grass pollen mix (Alutard®, ALK-Abelló, Hørsholm, Denmark), while the AIT used for donor 1, 5, and 6 contained no grass pollen allergens. Samples were obtained at treatment initiation, during vaccine up-dosing (at the stage when a dose of 10,000 SQ-U had been reached), and 1 and 3 years later (timepoint 1, 2, 3, and 4, respectively). The reactivity of these samples had previously been analyzed in-depth with respect to antibody responses against linear epitopes on pollen allergens of the beta-expansin and the PR-10 families (20).

Antibody binding patterns were evaluated using allergen peptide microarray slides. Quantification was, as described previously (20), done using Alexa Fluor 532 (for IgE) and Alexa Fluor 647 (for IgG4 and IgG) conjugated secondary antibodies and a NimbleGen MS200 scanner (Roche NimbleGen Inc.). Scanned images were converted into signal intensity values using GenePix Pro 5.1 image analysis program.

### Data Pre-processing

Candidate epitopes were determined as earlier described (20). In short, signal intensity values were log-transformed and candidate epitopes were defined by being represented by at least three adjacent peptides with a signal at least two standard deviations above the background signal for that sample.

To allow for comparison of homologous grass pollen allergens from different species and of isoallergens and variants, the amino acid sequences of all allergens belonging to the same group were aligned using MacVector Inc. (version 18.0.0). ClustalW was applied, using the Gonnet substitution matrix, open and extended gap penalty set to 10.00 and 0.20, respectively, and the slow pairwise alignment mode (Supplementary Table 1). Each peptide was assigned a number representing the position of its first amino acid, according to the alignment, and visualizations were made based on these numbers. Allergens belonging to grass pollen groups 2 and 3 as well as to groups 5 and 6 were aligned and visualized together.

Acidic ribosomal protein 1 allergens and acidic ribosomal protein 2 allergens, i.e., control allergens that were not included in the AIT, were also aligned and renumbered accordingly. Sequences of the human 60S acidic ribosomal protein 1 (P05386) and the human 60S acidic ribosomal protein 2 (P05387) were obtained from UniProt (27) and included in the alignments.

### Comparison of Linear Epitope Binding Before and After AIT

Differences in linear grass pollen-epitope recognition before and after AIT were evaluated for patients subjected to grass pollen AIT and compared with differences in epitope recognition during the same period for untreated patients and patients receiving AIT containing other allergens. First, non-reactive peptides, i.e., peptides that had not passed the candidate epitope filter, were set to zero. Background was calculated as the mean signal of a sample and subtracted from the signal intensity of all reactive peptides. Finally, the difference in signal between each peptide 3 years after treatment initiation (time 4) and the same peptide at the start of treatment (time 1) were calculated and visualized. For donors where the 3-year sample was missing, the 1-year sample was used instead. Additionally, the mean number of peptides with signal difference above 0 was compared for individuals who were or were not subjected to grass pollen vaccination. Mean values before and after AIT were calculated using peptide signal intensities of all grass pollen allergens of an allergen group and the number of peptides with higher mean value at time 4 (or in some cases time 3) compared to time 1 were counted, for each individual separately. Subsequently, the calculated values and Mann–Whitney *U*-test were used to compare the number of peptides with increased antigen binding over time in donors who had, or who had not, been subjected to grass pollen AIT. For some selected regions of group 5 and 6 grass pollen allergens, the mean signal intensities were also compared between the treatment initiation sample and the last collected sample. Average peptide signals for group 5 and 6 grass pollen allergens were calculated for each peptide in that region, each sample and donor separately. Differences between the time 1 and the time 3 or time 4 sample were evaluated using one-sided Wilcoxon signed-rank test, in order to identify individuals in whom the antibody response against the examined region increased during the investigated time period.

Subsequently, the total number of linear epitopes in all allergens belonging to either of three species of grass [*Lolium perenne* (English ryegrass), *Dactylis glomerata* (orchard grass), and *Phleum pratense* (timothy)] that are present in the mix used for AIT were evaluated. *Alopecurus pratensis* (meadow foxtail) and *Festuca pratensis* (meadow fescue) are also included in the grass pollen extract mix, but they had no available sequences in the allergen database used to design the peptide microarray and could therefore not be included in the analysis. The total number of continuous regions of peptides that were considered as reactive were calculated for each sample separately. To balance the impact of allergens with more than one known isoallergen or variant, mean values of number of epitopes were calculated for each allergen, based on all its isoallergens/variants, and used for calculation of the total number of epitopes of a sample. Finally, the number of linear epitopes recognized by antibodies in samples from donors who had, or who had not, been treated with grass pollen were compared and evaluated using Mann–Whitney *U*-test. The calculated values were based on six allergens (nine isoallergens/variants), four allergens, and 10 allergens (29 isoallergens/variants) originating in *Lolium perenne, Dactylis glomerata*, and *Phleum pratense*, respectively (Supplementary Table 1).

### Visualization of Epitope on Allergen Structures

Selected epitopes were visualized on 3D structures of allergens [obtained from PDB (28)], using PyMol version 2.5.2 (29). In cases where the region of reactive peptides could be expected to contain multiple close-by epitopes, the entire region of amino acids covered by the reactive peptides was highlighted in the structure. In other cases, only amino acids that were present in all reactive peptides were expected to be part of the epitope and consequently marked in the structure. Structures with PDB id 2VXQ (30), 2M64 (31) (model 6), and 4BEH (model 11) (32) were used for Phl p 2, Phl p 5, and acidic ribosomal protein 1 and 2, respectively.

### Estimation of Amino Acid Signal Values

For analysis of epitope patterns in different variants of Phl p 5.01 and Phl p 5.02, the peptide signal intensity values were transformed into estimated values for each amino acid. Background values were calculated for each sample and allergen, as the mean signal intensity of all peptides that were considered as non-reactive. All non-reactive peptides were given the background signal level, to avoid single, high false positive signals to have an impact on estimated amino acid signal values in the next step. Finally, signal values were estimated for each amino acid by calculating a mean value of all peptides that were covering that amino acid and dividing by the background signal. Hence, most amino acid values were based on 16 peptide signal values, but the 15 amino acids closest to the N- and C-termini were based on fewer peptide signal values with the N- and C-terminal amino acids based solely on the first and last peptide signal, respectively.

## Results

### Multiple Grass Pollen Allergens Induce Antibodies Against Diverse Linear Epitopes

We have recently developed an allergome-wide peptide microarray to study the IgE, IgG, and IgG4 responses to linear epitopes on some selected aeroallergens, for serum collected from allergic individuals during AIT (20). Here we exploited the collected data and the generated bioinformatic pipeline to evaluate the epitope recognition pattern against different grass pollen allergens in 15 allergic individuals, eight of which were subjected to AIT, five of whom with subcutaneous injections with a grass pollen mix (26). Linear epitopes recognized by IgG (Supplementary Figure 1) and IgG4 (Supplementary Figure 2) were identified in all examined groups of grass pollen allergens and generally seen already at time 1, i.e., before start of AIT. Antibodies of IgE isotype that bound allergen peptides were detected more rarely, and in most cases only after vaccination with grass pollen extract (Supplementary Figure 3). For grass pollen allergens belonging to group 7 and group 12, no linear epitopes were recognized by IgE at any timepoint.

### Vaccination Induces Antibodies Against Linear Epitopes on Grass Pollen Allergens

**Figure 1:**
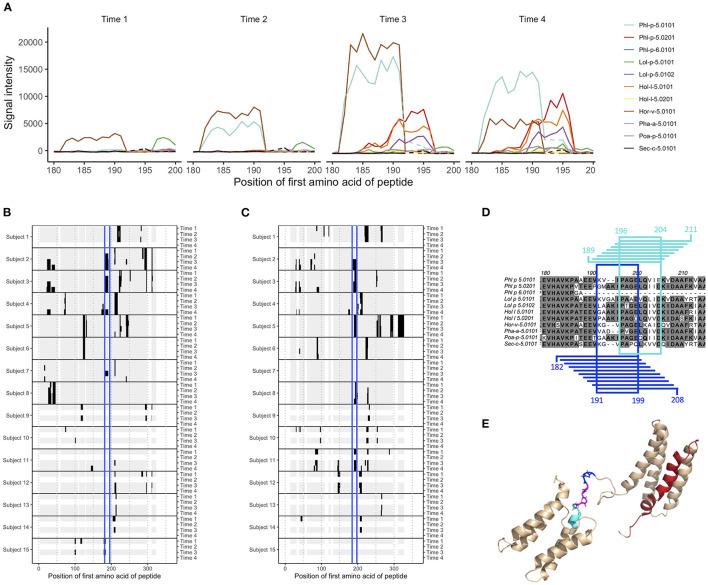
Epitope B on group 5 and 6 grass pollen allergens stretches over linear peptides with their first amino acid in position 182–196, and can be further divided into epitope B1 and epitope B2. **(A)** IgG peptide-binding signal intensities with background subtracted (defined as mean for each sample and isotype) for subject 2, in the region covering epitope B. Signal intensity plots for other donors and isotypes are presented in Supplementary Figure 8. Samples had been collected at AIT treatment initiation (time 1), and 8 weeks (time 2, when vaccine up-dosing had been reached), 1 year (time 3), and 3 years later (time 4). **(B,C)** Heatmap visualization of IgG recognition of linear epitopes on **(B)** Phl p 5.0101 and **(C)** Phl p 5.0201, with reactive peptides in black, non-reactive peptides in gray, and epitope B marked in blue. **(D)** Alignment of group 5 and 6 grass pollen allergens in the region covering epitope B. Peptides representing epitope B1 and their common amino acids are marked in blue, and peptides representing epitope B2 and their common amino acids are marked in cyan. **(E)** Solution structure (PDB id 2M64) of Phl p 5.0101 with epitope B1 (blue), B2 (cyan), their overlapping region (magenta), and epitope C (red) marked.

The mechanism through which AIT alters the progression of allergic disease is often suggested to include increased levels of protective allergen-specific IgG (in particular IgG4) (7, 8, 15). We examined if such increased levels of IgG/IgG4 could be seen against linear grass pollen epitopes by comparing the difference in estimated signal level for each peptide between the first and last sample collected for each individual, i.e., at treatment initiation and at 1 or 3 years later. Indeed, the levels of IgG and IgG4 against many linear epitopes increased for individuals vaccinated against grass, while increased levels of IgE were more rarely seen (Supplementary Figures 4–6). In individuals who had been subjected to AIT only containing pollen extract from other sources than grass, or who had not been subjected to AIT at all, the level of antibodies against linear grass pollen epitopes did generally not increase over time (Supplementary Figures 4–6). Indeed, statistical comparison of the number of peptides with increased signal intensity over time showed that these were often more common in donors who had been subjected to grass pollen AIT compared to donors who had not (Supplementary Figures 4–6). As an example, grass pollen allergens belonging to group 5 and 6 had multiple epitopes for which IgG4 appeared to increase after vaccination, for example in the peptides starting at amino acid 21–53 (epitope A) and at amino acid 182–196 (epitope B). We further examined the peptide signal intensities for these reactive regions identified in individual 2, 3, and 4. As expected, the IgG4 signals against the relevant peptides increased significantly during the treatment compared to baseline levels in all three individuals and against both peptides, except for donor 4 and epitope B (Figure 1A; Supplementary Figures 7, 8). Total epitope-specific IgG levels also often increased, but in many cases from higher starting levels and to a lower degree, indicating both an increased production of IgG against the examined epitopes and a shift in the relationship between IgG4 and other types of IgG.

**Figure 2:**
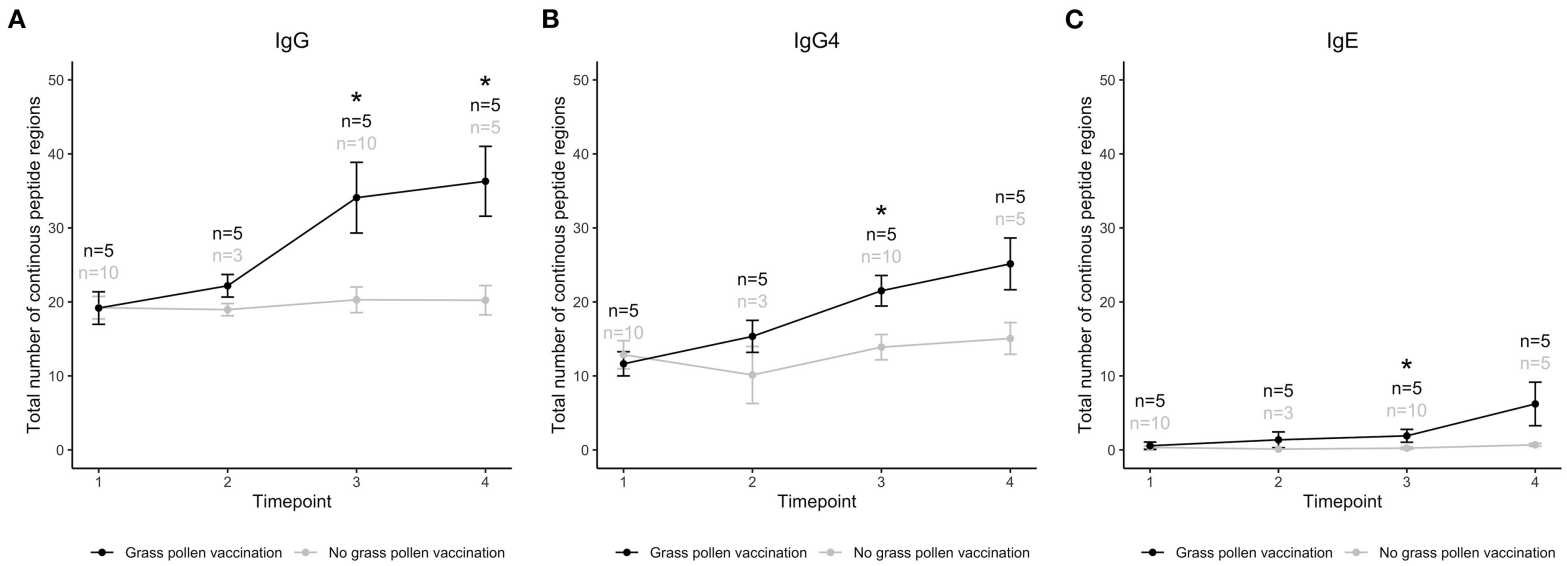
Total number of linear epitopes in allergens of three grass species (*Lolium perenne, Dactylis glomerata, Phleum pratense*) recognized by IgG **(A)**, IgG4 **(B)**, and IgE **(C)** in individuals that had (black) or had not (gray) been treated with AIT containing extract from these three, and two additional (*Alopecurus pratensis* and *Festuca pratensis*), grass species. Samples had been collected at treatment initiation (time 1), and 8 weeks (time 2), 1 year (time 3), and 3 years later (time 4). *Timepoints with a significant difference between treated and untreated individuals, using Mann–Whitney *U*-test and *p*-value < 0.05.

As exemplified by these epitopes, AIT can introduce antibody responses against linear epitopes not recognized by that isotype before treatment. In order to examine if vaccination generally induced production of antibodies of IgG, IgG4, and IgE isotype against novel epitopes, we also studied the immune response against the total number of linear epitopes in allergens from grass species present in the used vaccine before, during, and after AIT (Figure 2). Before treatment (time 1), the number of epitopes were similar in individuals that were to and in individuals that were not to be subjected to grass AIT. During treatment, the number of epitopes recognized by IgG, IgG4, and IgE isotypes increased, and became significantly higher than for the groups of subjects not subjected to AIT after 1 year, i.e., at timepoint 3 (Figure 2). After 3 years (timepoint 4), significant difference between the groups were only seen for epitopes recognized by IgG, likely due to a lower number of samples collected in the unvaccinated group at this timepoint compared to at timepoint 3.

### Epitope Profiles Commonly Remain Stable Over Time, but Antibodies Against One Linear Epitope on Group 5 Allergens Are Commonly Developed During AIT

**Figure 3:**
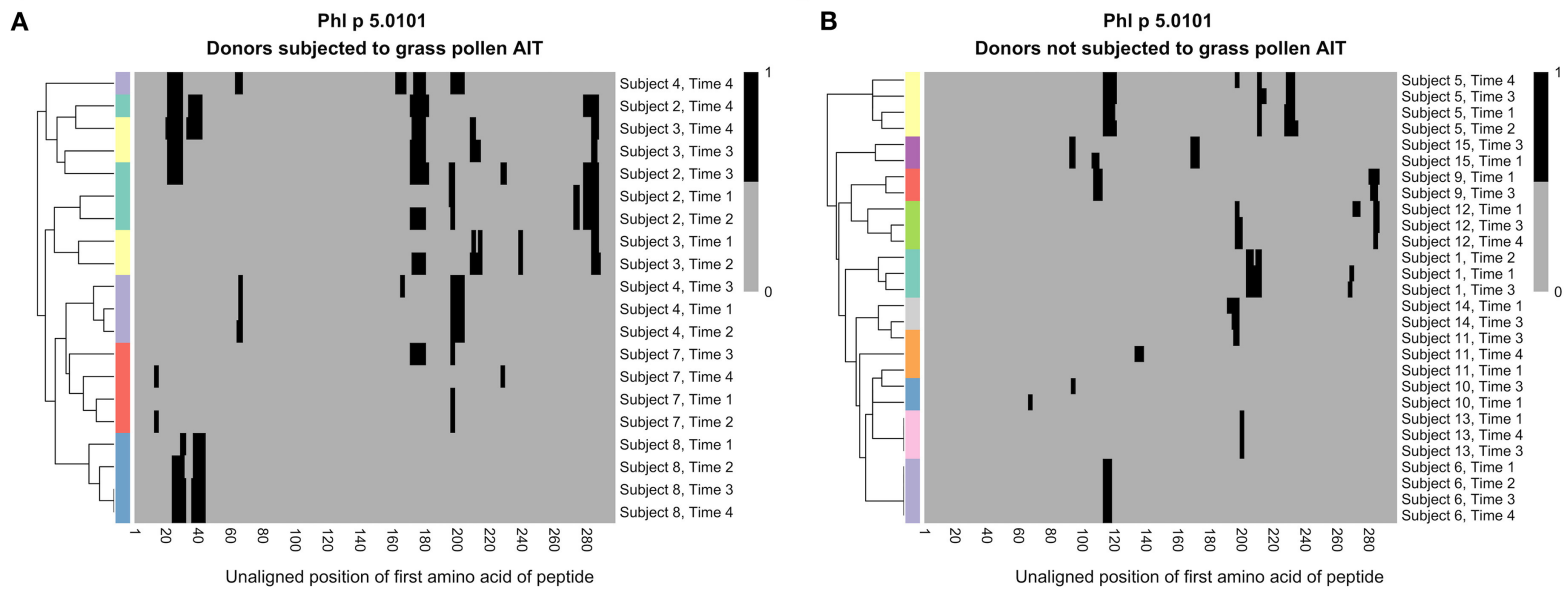
Hierarchical clustering of IgG recognition of Phl p 5.0101 peptides in serum samples collected at AIT treatment initiation (time 1), and 8 weeks (time 2), 1 year (time 3), and 3 years later (time 4). Donor 2 (cyan), donor 3 (yellow), donor 4 (light purple), donor 7 (red), and donor 8 (blue) had been subjected to grass pollen AIT **(A)**, while donor 1 (cyan), donor 5 (yellow), and donor 6 (light purple) had been subjected to AIT containing non-grass allergens. Donor 9 (red), donor 10 (blue), donor 11 (orange), donor 12 (green), donor 13 (pink), donor 14 (gray), and donor 15 (dark purple) had not been subjected to AIT at all **(B)**. Clustering was done using euclidean distance and the R package “pheatmap”.

As the level of IgG against certain epitopes increased during grass pollen AIT, we wanted to examine if the overall profile of epitopes recognized by IgG remained stable for each subject or if AIT introduced some common profile patterns. Hierarchical clustering of epitope profiles against Phl p 5.0101 (Figure 3), Phl p 2.0101, and Phl p 6.0101 (Supplementary Figure 9), showed that IgG and IgG4 peptide recognition were more similar in samples of the same subject collected over time than in samples of different individuals, independently if the patient had been subjected to grass pollen AIT or not, implying that allergen-epitope profiles are highly individual.

Still, some shared patterns could be seen in many of the vaccinated individuals. One epitope (epitope B), found at Phl p 5.0101, was not recognized by IgG or IgG4 from any of the studied individuals at time 1 (Figure 1B). During grass pollen AIT, however, four out of five individuals developed IgG and IgG4 against this epitope. One of these, subject 2, expressed IgE against the same epitope during the entire time course, i.e., even before treatment initiation. For individuals not subjected to AIT containing grass pollen, no antibodies could be identified against this epitope (Figure 1B).

### Epitope-Specific Recognition of Allergens Across Species and Isoallergens

With peptides from 731 different allergens, the microarray platform allows for a detailed study of antibody cross-reactivity. Comparison of antibody binding patterns between allergens from different grass species, belonging to group 2 and 3 or to group 5 and 6, showed that while some linear epitopes were unique for one single grass pollen allergen, many were seen in two or more allergens of the same group (Supplementary Figures 1–3). Epitope B at Phl p 5.0101, for which IgG and IgG4 were commonly developed during AIT, could also be identified in other grass pollen allergens of group 5 and 6, including the isoallergen Phl p 5.0201 (Figure 1C), even though Phl p 5.0101 (and its related variants Phl p 5.0102-Phl p 5.0109) has a two amino acid deletion (Figure 1D) within this region. In comparison with Phl p 5.0101, multiple individuals expressed IgG against this epitope on Phl p 5.0201 already at time 1, suggesting that it is not the same IgG binding to epitope B on these two isoallergens. Indeed, a more detailed study of this region showed that the binding patterns were slightly different between different isoallergens, and that the region more likely carries two, slightly overlapping epitopes: epitope B1 which is found on group 5 and 6 grass pollen allergens with the amino acid deletion and epitope B2 which is found on allergens lacking this deletion (Figures 1A,D; Supplementary Figure 8). Notably, some individuals express antibodies against only one of these epitopes, such as subject 4 (only recognizes epitope B1) and subject 11 (only recognizes epitope B2), while others (e.g., subject 2 and 3) express antibodies against both epitopes. The antibodies recognizing epitope B1 and B2 often also bind homologous allergens from other grass species that contain (Hor v 5.0101 and Sec C 5.0101) or do not contain (Lol p 5.0101, Lol p 5.0102, Hol l 5.0101, Hol l 5.0102, Poa P 5.0101) the two amino acid deletion, respectively (Supplementary Figure 8). A major part of epitope B1 and the entire epitope B2 reside in the flexible linker that connects the allergens' N- and C-terminal domains (Figure 1E).

### Allergen Recognition Is Typically Restricted to Different Allergen Variants

Phl p 5 has two known isoallergens (Phl p 5.01 and Phl 5.02) that are further divided into nine and seven different known variants (Phl p 5.0101-Phl p 5.0109 and Phl p 5.0201-Phl p 5.0207). Peptides from each of these variants have been included in the peptide microarray, thereby enabling us to examine the antibody binding pattern deviations between the different isoforms. We transformed the peptide signal intensity data into estimated signal intensities for each amino acid, to better follow the effect of small sequence differences on antibody recognition. Differences between variants, even if being limited to only one amino acid residue, often had a clear effect on the binding of antibodies, resulting in either decreased or completely absent binding signals (Supplementary Figures 10, 11). Similar as for epitope B1 and B2, binding patterns varied to a great extent between different individuals, as highlighted by for example epitope C, which covers residue 79 (Supplementary Figure 12) in Phl p 5.0101-Phl p 5.0109 and is situated in one of the alpha helices of the allergens' N- terminal domain (Figure 1E). This position may carry either an alanine or an aspartate amino acid. Antibodies found in subjects 2, 12, and 15 recognize peptides with an aspartate in this position but bind peptides with alanine to a lower degree (with signals too low for passing the cut-off) or not at all. Antibodies found in other individuals (3 and 4) instead prefer peptides with an A79. In one individual (donor 7) IgG signals were only seen for D79 peptides, while IgG4 signals only could be measured for A79 peptides.

### Linear Epitopes on Group 2 Grass Pollen Allergen Complement Allergen Recognition Defined by Public Antibody Response to Group 2 Allergens

**Figure 4:**
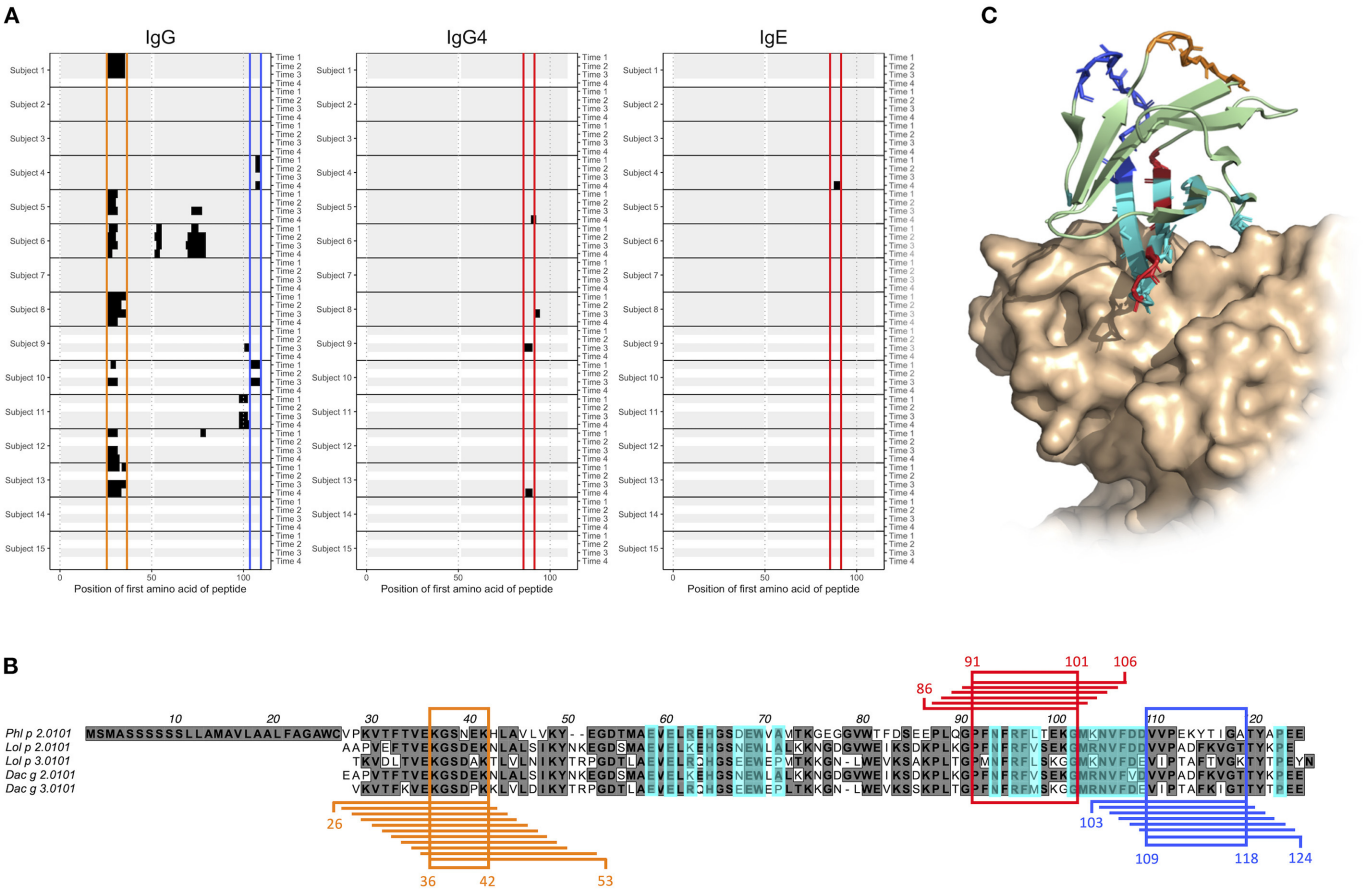
Epitope D (red), E (orange), and F (blue) are found at Phl p 2.0101 and represented by peptides starting with amino acid 86–91, 26–36, and 103–109, respectively. **(A)** Phl p 2.0101 linear epitope binding pattern of IgG, IgG4, and IgE in serum samples of allergic individuals collected at start of AIT treatment (time 1), and 8 weeks (time 2), 1 year (time 3), and 3 years later (time 4). Epitope D (red), E (orange), and F (blue) have been marked. **(B)** Alignment of group 2 grass pollen allergens, with peptides representing epitope D (red), E (orange), and F (blue), and the common amino acid of all peptides in an epitope highlighted. A previously described (30) discontinuous epitope is also marked (cyan). **(C)** Crystal structure (PDB id 2VXQ) of Phl p 2.0101 with epitope D (red), E (orange), and F (blue) marked together with a previously described (30) discontinuous epitope (cyan), known to be recognized by stereotyped IgE seen in many allergic individuals (23–25).

We and others (23–25) have previously identified antibodies that represent a so-called stereotyped/public IgE response against the timothy grass pollen allergen Phl p 2. Stereotyped antibodies develop in multiple individuals in response to a particular antigen, in this case Phl p 2, and feature a set of similar, common sequence characteristics. The binding of antibodies with these features has been associated with a discontinuous epitope (30), located mainly within a four-stranded β-sheet of the Phl p 2 allergen, and shown to largely inhibit the binding of IgE from grass pollen-allergic subjects to Phl p 2 (30). Now, we examined the generated microarray peptide data aiming to identify epitopes that are located elsewhere in Phl p 2, and that thus may be of relevance in the IgE crosslinking of FcεRI on effector cells in the presence of dominant stereotyped antibodies. The binding of IgE to linear peptides could only be identify in one individual, but the same epitope (epitope D) was also bound by IgG4 in two subjects (Figures 4A,B). Additionally, two major epitopes (epitope E and F) found in seven and two subjects, respectively, were seen in the IgG data. These two epitopes were found at a distance from both each other and the previously determined epitope of Phl p 2 stereotyped IgE, while epitope D partly overlapped with the conformational epitope (Figure 4C). Additional linear epitopes were seen in group 2 and 3 allergens of other species (Supplementary Figures 1, 2) including some that based on homology to the structure of Phl p 2 reside at a distance from the epitope recognized by stereotyped antibodies (data not shown).

### Commonly Recognized Linear Epitopes on Ribosomal Protein P1 and P2 Allergens Reside in Their Flexible Arms

**Figure 5:**
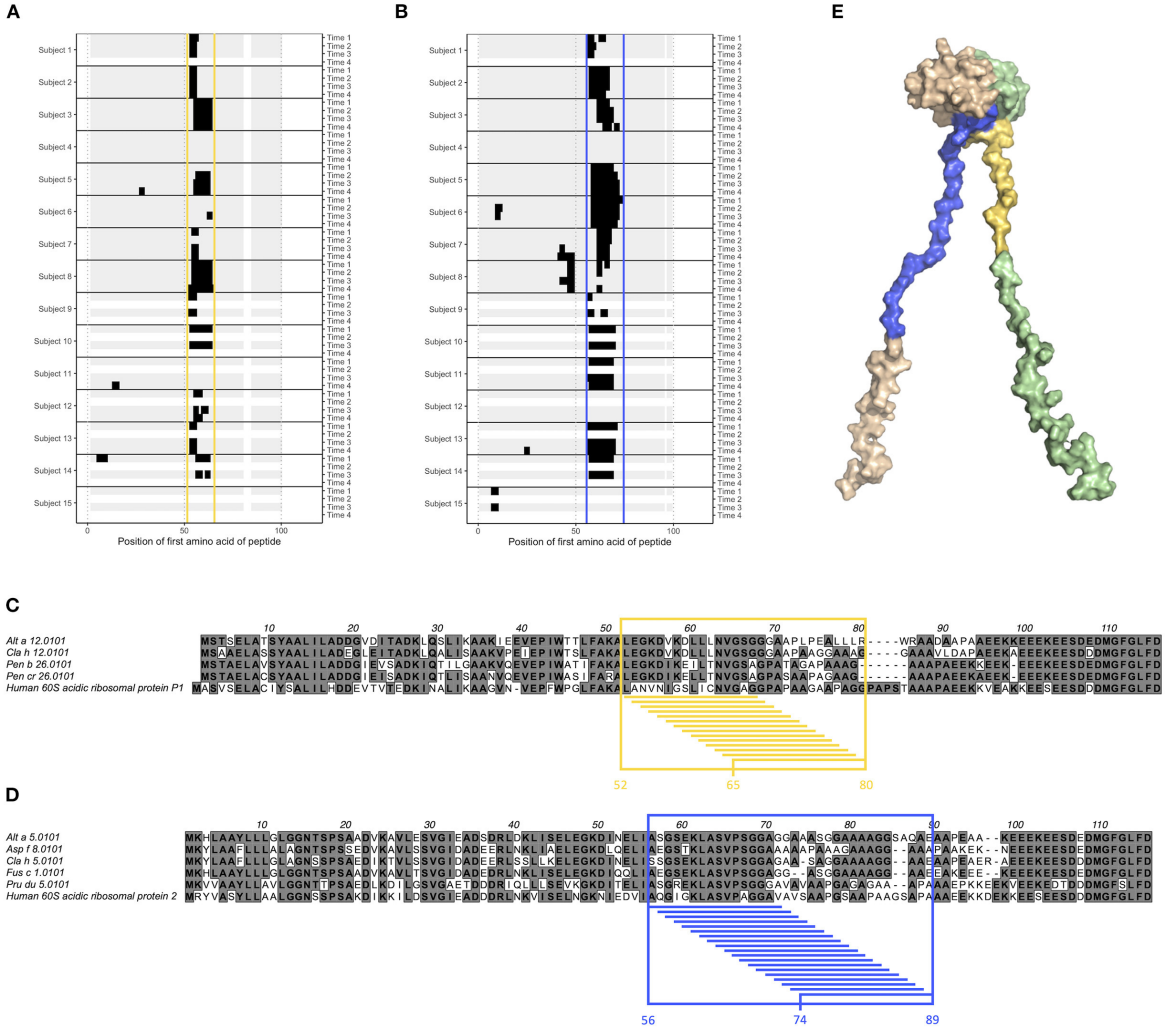
Epitope G (yellow) and H (blue) are found on acidic ribosomal protein 1 allergens and acidic ribosomal protein 2 allergens, respectively. **(A,B)** Linear epitope recognition pattern of IgG against the acidic ribosomal protein 1 allergen Alt a 12.0101 **(A)** and the acidic ribosomal protein 2 allergen Alt a 5.0101 **(B)** with epitope G (yellow) and H (blue) marked. IgG were sampled from allergic individuals at start of AIT treatment (time 1), and 8 weeks (time 2), 1 year (time 3), and 3 years later (time 4). **(C,D)** Alignment of acidic ribosomal protein 1 allergens together with human acidic ribosomal protein 1 **(C)**, and of acidic ribosomal protein 2 allergens together with human acidic ribosomal protein 2 **(D)**. Reactive peptides of epitope G (yellow) and H (blue) have been highlighted, together with all amino acids present in at least one of these peptides. **(E)** Solution structure (PDB id 4BEH) of human acidic ribosomal protein 1 (green) and 2 (beige) heterodimer with epitope G (yellow) and H (blue) marked.

Epitope B1 and B2 on grass pollen allergens belonging to group 5 were situated in the flexible linker of the allergen (Figure 1). We also examined another type of allergens with long, flexible arms; the acidic ribosomal protein P1 and P2. All examined individuals expressed IgG against linear epitopes on these two groups of allergens (Supplementary Figure 13), but no epitopes bound by IgE could be identified (data not shown), and only very limited IgG4 response was seen (Supplementary Figure 14). Much of the IgG reactivity was identified in one distinct region of allergens belonging to acidic ribosomal protein P1 (peptides starting at position 52–65, named epitope G) (Figures 5A,C) and in one distinct region of allergens belonging to acidic ribosomal protein P2 (peptides starting at position 56–74, named epitope H) (Figures 5B,D). Indeed, both these epitopes were found in the flexible arms of the allergen, close to their N-terminal domains (Figure 5E).

## Discussion

Analysis of antibody-binding linear peptides from allergenic proteins provides an opportunity to study allergen-specific antibodies in a high-throughput/high-content manner. Here, we employed a previously designed allergome-wide peptide microarray (20) to track the antibody-binding pattern against linear epitopes on grass pollen allergens, in samples collected from allergic individuals during 3 years of AIT. In order to expand an earlier study, which focused on the response against birch and grass pollen allergens of the PR-10 and beta-expansin families (20), we have now focused on antibodies specific against other common grass pollen allergen groups. As expected, we identified an increase in antibody response against linear epitopes on these allergens in individuals who had been subjected to grass pollen AIT, compared to before treatment initiation or to untreated allergic individuals. Such an increase was identified when comparing the signal intensities before and after vaccination, but also when examining the total number of linear epitopes on allergens from grass species present in the grass pollen mix extract used for the AIT. Notably, the exact allergen content of this grass pollen mix was not known and allergens of all grass species in the mix were not represented in the allergen peptide microarray as they have not yet been molecularly defined. It is likely that the observed effect would have been even more pronounced if the allergens included in analysis had better resembled the content of the vaccine.

Group 5 allergens, including Phl p 5, are known to be highly allergenic, and consist of two domains linked by a flexible region. The high allergenic potential of Phl p 5 has been linked to e.g., the high number of discontinuous epitopes located within its two rigid domains (33), and to the mobility induced by the flexible linker, which facilitates a higher degree of cross-linking of FcεRI on effector cells (31). Indeed, multiple human monoclonal IgE that bind such conformational epitopes have been identified (33). Here we have identified linear epitopes located within this flexible linker of the group 5 allergens, which may contribute to the allergenicity of these allergens when recognized by IgE. The results highlight not only the importance of this region for Phl p 5 as an allergy-causing protein, but also for the resolution of the disease when bound by antibodies of other isotypes. Indeed, the grass pollen vaccine used in the present study was, in some subjects, able to expand pre-existing humoral immunity or induce new responses to this linear epitope. Furthermore, a recombinant hypoallergenic variant of Phl p 5, incorporated into the BM32 grass pollen vaccine (34), carries part of this linker, a sequence that may contribute to its allergen-specific immune response (35). Areas of linear epitopes were also identified within the flexible arms found in acidic ribosomal protein 1 and 2 allergens. Such flexible sections may induce linear epitopes to a higher degree than more rigid regions, as their lack of ordered structure may restrain their incorporation in conformational epitopes. Indeed, MacRaild et al. have presented evidence that epitopes within discorded regions of antigens consistently are of linear nature (36). Hence, tracking of linear epitopes using for example peptide microarrays may be of particular relevance when studying antigens containing flexible regions. Such studies should, however, pay notice to that the high abundance of linear epitopes within these regions may result in multiple adjacent epitopes being interpreted as one long epitope. This is exemplified by epitope B, found in the flexible linker of group 5 and 6 grass pollen allergens, that could be subdivided into epitope B1 and B2. Similarly, the identified reactive regions within the flexible arms of acidic ribosomal protein 1 and 2 allergens may in fact also be composed of multiple adjacent or overlapping epitopes, as they are notably longer than 12 amino acids, an uncommon feature for linear epitopes (36).

Phl p 2 is a major grass pollen allergen for which many allergic individuals express stereotyped antibodies (23–25). These antibodies bind a conformational epitope located mainly in one of the beta sheets of the allergen (30). We present three novel linear epitopes on Phl p 2.0101, of which two (epitope E and F) are located distantly from both each other and the previously described discontinuous epitope. They may thus, in an individual expressing both IgE against any of them and stereotyped Phl p 2-specific IgE, be of importance in the cross-linking of FcεRI, and consequently in the release of inflammatory mediators and downstream symptoms of allergic disease. The third epitope, epitope D, on the other hand partly overlap with the previous described conformational epitope. It is thus possible that the IgG4 seen against this epitope in subjects 9 and 13, if they also recognize the folded antigen as presented to the immune system during an allergic response, can act protectively, by blocking the binding of stereotyped IgE. Not all allergic individuals with Phl p 2-specific IgE are able to express stereotyped antibodies, as an individual must possess specific immunoglobulin genes in its germline to do so. These genes, specifically IGHV4-30-4 and IGHV4-31 (24), are deleted in the genotypes of some subjects (37, 38). Also in these individuals, antibodies against one or multiple of the herein described linear epitopes may to some extent constitute the Phl p 2-specific antibody population. Donor 10 lacks the required immunoglobulin germline genes for expressing stereotyped antibodies (data not shown), and indeed express IgG against linear epitope F (Figure 4A).

As the herein used allergen peptide microarray contains peptides derived from many different allergens, isoallergens, and variants, it allows for examination of antibody cross-reactivity against homologous allergens from different grass sources and against highly similar isoallergens/variants. Group 5 and 6 allergens are well-suited for such analyses as they contain many different known allergens including nine and seven variants of the isoallergens Phl 5.01 and Phl p 5.02, respectively. Indeed, we identify multiple epitopes that induce cross-reactive antibodies, which recognize allergens also from grass species not included in the used vaccine. These findings are well in line with what we previously have described for allergens belonging to the PR-10 and beta-expansin groups (20). We also present evidence that small variations in the amino acid sequence—even single residue variations—can have a great impact on the binding of allergen-specific antibodies. For example, antibodies against epitope C, which stretches over residue 79 of Phl p 5.01 (represented by either an alanine or an aspartate), recognized either both variants, only variants carrying alanine, or only variants carrying aspartate (Supplementary Figure 12). Additionally, epitope B on group 5 and 6 allergens first appeared to be recognized by cross-reactive antibodies in many individuals, independently if the allergen contained a deletion of residue 194 and 195 or not. A more detailed analysis, however, showed that this epitope could be further divided into epitope B1, seen on allergens containing the deletion, and epitope B2, located on allergens lacking the deletion. Likely, these two epitopes were recognized by different antibody clones, also in individuals where reactivity against both type of allergens were seen. Our findings emphasize the importance of studying binding to different variants and isoallergens to determine the full allergen reactivity, and that multiple antibody clonotypes may contribute to recognition of a linear, peptide-defined epitope. In this perspective, the presented peptide microarray, with its full allergome coverage, constitutes a valuable tool in the study of linear epitopes.

The cross-reactivity pattern seen against e.g., epitope B and C of group 5 allergens was non-generic and varied from donor to donor. Similarly, hierarchical clustering of the binding pattern against Phl p 2.0101, Phl p 5.0101, and Phl p 6.0101 showed that samples of the same individual collected at different timepoints were more alike than samples obtained from different donors, independently if the donors had been vaccinated against grass pollen or not. These results do not only emphasize that the binding pattern against linear epitopes is highly individual, but also suggest that it is common for individual clonotypes that produce allergen-specific antibodies to be retained over a period of several years, even during AIT and that following establishment of the immune response its subsequent evolution is not an entirely stochastic process but originates in the pre-establish repertoire. We envisage that our findings, despite being represented by linear epitopes solely, can be generalized to the development and maintenance of allergen-specific immunity also against conformational epitopes. In fact, another study using the same serum samples showed that clones of cells producing allergen-specific antibodies binding to folded allergens could persist for at least 1 year during AIT (26).

To conclude, we have exploited an allergome-wide peptide microarray to describe the antibody binding pattern against linear grass pollen epitopes during AIT. As exemplified by multiple highlighted epitopes, we were able to track the evolution of linear epitope-specific antibodies of IgG, IgG4, and IgE isotype, evaluate their cross-reactivity against homologous allergens, isoallergens, and variants, and visualize their location on 3D structure to describe how antibodies against these and other epitopes may interact to block or induce cross-linking of effector cell FcεRI. Altogether, these finding emphasize the value of the used microarray, and how it can be utilized to enhance the understanding of antibodies in allergy and during AIT.

## Data Availability Statement

Summary data of samples clinical samples studied here have been published elsewhere (20, 26) and NGS read data relevant to the interpretation of clonal persistence have been submitted to SRA (Bioproject: PRJNA391821). Microarray data are available from the authors upon reasonable request.

## Ethics Statement

The study was approved by the regional ethics board at Lund University and performed according to the declaration of Helsinki. The patients/participants provided their written informed consent to participate in the study.

## Author Contributions

LT, MvH, and MO conceived the study. LG provided clinical samples. RS provided processed data for analysis. LT and RS planned the analysis pipeline. LT performed the analysis and wrote the manuscript draft. All authors performed data interpretation and commented on and approved the final version of the manuscript.

## Funding

This study was supported by a grant from The Swedish Research Council [grant no. 2019-01042 (MO)] and [2019-01060 (MvH)], The Swedish Heart Lung Foundation, The Cancer and Allergy Foundation, The Swedish Asthma and Allergy Association's Research Foundation, The Region Stockholm (ALF project), The European Union's Horizon 2020 [FoodEnTwin (GA 810752 (MvH)] and Imptox [RIA 965173 (MvH)], The Hesselman Foundation, and The King Gustaf V 80th Birthday Foundation. Lund University Library in part supported open access publication.

## Conflict of Interest

The authors declare that the research was conducted in the absence of any commercial or financial relationships that could be construed as a potential conflict of interest.

## Publisher's Note

All claims expressed in this article are solely those of the authors and do not necessarily represent those of their affiliated organizations, or those of the publisher, the editors and the reviewers. Any product that may be evaluated in this article, or claim that may be made by its manufacturer, is not guaranteed or endorsed by the publisher.
