# Supplementary material for: Linear Epitope Binding Patterns of Grass Pollen-Specific Antibodies in Allergy and in Response to Allergen-Specific Immunotherapy
Source: Front Allergy. 2022 Mar 31;3:859126. doi: 10.3389/falgy.2022.859126 (PMC9234942; doi:10.3389/falgy.2022.859126)
Supplement: Supplementary file 1 [file Data_Sheet_1.ZIP › Supplementary Table 1.docx]

**Supplementary Table 1.** Aligned amino acid sequences of allergens analyzed in this study. Sequences were obtained as described previously (20) and aligned in MacVector Inc (version 18.0.0), using ClustalW with the Gonnet substitution matrix, open and extended gap penalty set to 10.00 and 0.20, respectively, the slow pairwise alignment mode. Sequences of human 60S acidic ribosomal protein P1 and P2 were obtained from UniProt (27) (id P05386 and P05387).

| **Grass pollen group 1 allergens** | |
| --- | --- |
| **Allergen** | **Aligned sequence** |
| Phl p 1.0101 | ------MASSSSVLLVVALFAVFLGSAHGIPKVPPGPNITATYGDKWLDAKSTWYGKPTA  AGPKDNGGACGYKDVDKPPFSGMTGCGNTPIFKSGRGCGSCFEIKCTKPEACSGEPVVVH  ITDDNEEPIAAYHFDLSGIAFGSMAKKGDEQKLRSAGEVEIQFRRVKCKYPEGTKVTFHV  EKGSNPNYLALLVKFVAGDGDVVAVDIKEKGKDKWIALKESWGAIWRIDTPEVLKGPFTV  RYTTEGGTKGEAKDVIPEGWKADTAYESK |
| Phl p 1.0102 | ------MASSSSVLLVVVLFAVFLGSAYGIPKVPPGPNITATYGDKWLDAKSTWYGKPTG  AGPKDNGGACGYKDVDKPPFSGMTGCGNTPIFKSGRGCGSCFEIKCTKPEACSGEPVVVH  ITDDNEEPIAPYHFDLSGHAFGAMAKKGDEQKLRSAGELELQFRRVKCKYPEGTKVTFHV  EKGSNPNYLALLVKYVNGDGDVVAVDIKEKGKDKWIELKESWGAIWRIDTPDKLTGPFTV  RYTTEGGTKTEAEDVIPEGWKADTSYESK |
| Lol p 1.0101 | ------MASSSSVLLVVALFAVFLGSAHGIAKVPPGPNITAEYGDKWLDAKSTWYGKPTG  AGPKDNGGACGYKNVDKAPFNGMTGCGNTPIFKDGRGCGSCFEIKCTKPESCSGEAVTVT  ITDDNEEPIAPYHFDLSGHAFGSMAKKGEEQNVRSAGELELQFRRVKCKYPDDTKPTFHV  EKASNPNYLAILVKYVDGDGDVVAVDIKEKGKDKWIELKESWGAVWRIDTPDKLTGPFTV  RYTTEGGTKSEFEDVIPEGWKADTSYSAK |
| Lol p 1.0102 | -----------------ALFAVFLGSAHGIAKVPPGPNITAEYGDKWLDAKSTWYGKPTG  AGPKDNGGACGYKDVDKAPFNGMTGCGNTPIFKDGRGCGSCFEIKCTKPESCSGEAVTVT  ITDDNEEPIAPYHFDLSGHAFGSMAKKGEEQNVRSAGELELQFRRVKCKYPDDTKPTFHV  EKGSNPNYLAILVKYVDGDGDVVAVDIKEKGKDKWIELKESWGAVWRIDTPDKLTGPFTV  RYTTEGGTKSEVEDVIPEGWKADTSYSAK |
| Lol p 1.0103 | ------MASSSSVLLVVALFAVFLGSAHGIAKVPPGPNITAEYGDKWLDAKSTWYGKPTG  AGPKDNGGACGYKDVDKAPFNGMTGCGNTPIFKDGRGCGSCFEIKCTKPESCSGEAVTVT  ITDDNEEPIAPYHFDLSGHAFGSMAKKGEEQKLRSAGELELQFRRVKCKYPDGTKPTFHV  EKASNPNYLAILVKYVDGDGDVVAVDIKEKGKDKWIELKESWGAVWRIDTPDKLTGPFTV  RYTTEGGTKSEVEDVIPEGWKADTSYSAK |
| Hol l 1.0101 | ------MASSSLVLLVVALFAVFLGTAHGIAKVPPGPNITATYGDKWLDAKSTWYGKPTG  AGPKDNGGACGYKDVDKPPFSGMTGCGNTPIFKSGRGCGSCFEIKCTKPESCSGEPIVVH  ITDDNEEPIAAYHLDLSGKAFGAMAKKGEEQKLRSAGELELKFRRVKCEYPKGTKVTFHV  EKGSNPNYLALLVKYVDGDGDVVAVDIKEKGKDKWIELKESWGAVWRVDTPDKLTGPFTV  RYTTEGGTKVEAEDVIPEGWKADTAYESK |
| Hol l 1.0102 | ---------------------VFLGSAHGIAKVPPGPNITATYGDEWLDAKSTWYGKPTG  AGPKDNGGACGYKDVDKPPFSGMTGCGNTPIFKDGRGCGSCFEIKCSKPESCSGEPVTVH  ITDDNEEPIAPYHFDLSGHAFGSMAKKGEEQKLRSAGELELKFRRVKCKYPDGTKPTFHV  EKGSNPNYLALLVKYIDGDGDVVAVDIKEKGKDKWIELKESWGAVWRVDTPDKLTGPFTV  RYTTEGGTKGEAEDVIPEGWKADTAYEAK |
| Pha a 1.0101 | MMKMVCSSSSSSLLVVAALLAVFVGSAQGIAKVPPGPNITAEYGDKWLDAKSTWYGKPTG  AGPKDNGGACGYKDVDKAPFNGMTGCGNTPIFKDGRGCGSCFELKCSKPESCSGEPITVH  ITDDNEEPIAPYHFDLSGHAFGSMAKKGEEENVRGAGELELQFRRVKCKYPDGTKPTFHV  EKGSNPNYLALLVKYVDGDGDVVAVDIKEKGKDKWIELKESWGAIWRIDTPDKLTGPFTV  RYTTEGGTKAEFEDVIPEGWKADTHDASK |
| Poa p 1.0101 | ------MASSSSVLLVVALFAVFLGTAHGIAKVPPGPNITATYGDKWLDAKSTWYGKPTG  AGPKDNGGACGYKDVDKAPFSGMTGCGNTPIFKSGRGCGSCFEIKCTKPESCSGEPVLVH  ITDDNEEPIAAYHFDLSGKAFGAMAKKGEEQKLRSAGELELKFRRVKCEYPEGTKVTFHV  EKGSNPNYLALLVKYVTGDGDVVAVDIKEKGKDKWIELKESWGSIWRVDTPDKLTGPFTV  RYTTEGGTKGEAEDVIPEGWKADTAYASK |

| **Grass pollen group 2 and 3 allergens** | |
| --- | --- |
| **Allergen** | **Aligned sequence** |
| Phl p 2.0101 | MSMASSSSSSLLAMAVLAALFAGAWCVPKVTFTVEKGSNEKHLAVLVKY--EGDTMAEVE  LREHGSDEWVAMTKGEGGVWTFDSEEPLQGPFNFRFLTEKGMKNVFDDVVPEKYTIGATY  APEE- |
| Lol p 2.0101 | --------------------------AAPVEFTVEKGSDEKNLALSIKYNKEGDSMAEVE  LKEHGSNEWLALKKNGDGVWEIKSDKPLKGPFNFRFVSEKGMRNVFDDVVPADFKVGTTY  KPE-- |
| Lol p 3.0101 | ---------------------------TKVDLTVEKGSDAKTLVLNIKYTRPGDTLAEVE  LRQHGSEEWEPMTKKGN-LWEVKSAKPLTGPMNFRFLSKGGMKNVFDEVIPTAFTVGKTY  TPEYN |
| **Grass pollen group 4 allergens** | |
| **Allergen** | **Aligned sequence** |
| Dac g 2.0101 | --------------------------EAPVTFTVEKGSDEKNLALSIKYNKEGDSMAEVE  LKEHGSNEWLALKKNGDGVWEIKSDKPLKGPFNFRFVSEKGMRNVFVDVVPADFKVGTTY  KPEE- |
| Dac g 3.0101 | ---------------------------VKVTFKVEKGSDPKKLVLDIKYTRPGDTLAEVE  LRQHGSEEWEPLTKKGN-LWEVKSSKPLTGPFNFRFMSKGGMRNVFDEVIPTAFKIGTTY  TPEE- |
| Phl p 4.0101 | SSCEVALSYYPTPLAKEDFLRCLVKEIPPRLLYAKSSPAYPSVLGQTIRNSRWSSPDNVK  PIYIVTPTNASHIQSAVVCGRRHGVRIRVRSGGHDYEGLSYRSLQPEEFAVVDLSKMRAV  WVDGKARTAWVDSGAQLGELYYAIHKASPVLAFPAGVCPTIGVGGNFAGGGFGMLLRKYG  IAAENVIDVKLVDANGTLHDKKSMGDDHFWAVRGGGGESFGIVVAWKVRLLPVPPTVTVF  KIPKKASEGAVDIINRWQVVAPQLPDDLMIRVIAQGPTATFEAMYLGTCQTLTPMMSSKF  PELGMNASHCNEMSWIQSIPFVHLGHRDNIEDDLLNRNNTFKPFAEYKSDYVYEPFPKEV  WEQIFSTWLLKPGAGIMIFDPYGATISATPEWATPFPHRKGVLFNIQYVNYWFAPGAGAA  PLSWSKEIYNYMEPYVSKNPRQAYANYRDIDLGRNEVVNDVSTFSSGLVWGQKYFKGNFQ  RLAITKGKVDPTDYFRNEQSIPPLIQKY |
| Phl p 4.0201 | SSCQVAFSYFPPPAAKEDFLGCLVKEIPPRLLYAKSSPAYPSVLGQTIRNSRWSSPDNVK  PLYIITPTNVSHIQSAVVCGRRHSVRIRVRSGGHDYEGLSYRSLQPETFAVVDLNKMRAV  WVDGKARTAWVDSGAQLGELYYAIYKASPTLAFPAGVCPTIGVGGNFAGGGFGMLLRKYG  IAAENVIDVKLVDANGKLHDKKSMGDDHFWAVRGGGGESFGIVVAWQVKLLPVPPTVTIF  KISKTVSEGAVDIINKWQVVAPQLPADLMIRIIAQGPKATFEAMYLGTCKTLTPLMSSKF  PELGMNPSHCNEMSWIQSIPFVHLGHRDALEDDLLNRNNSFKPFAEYKSDYVYQPFPKTV  WEQILNTWLVKPGAGIMIFDPYGATISATPESATPFPHRKGVLFNIQYVNYWFAPGAAAA  PLSWSKDIYNYMEPYVSKNPRQAYANYRDIDLGRNEVVNDVSTYASGKVWGQKYFKGNFE  RLAITKGKVDPTDYFRNEQSIPPLIKKY |
| Lol p 4.0101 | ------------------------------------------------------------  ---------------AVVCGRRYDVRIRVRSGGHDYEGLSYRSLQPENFAVVDLNQMRAV  LVDGKARTAWVDSGAQLGELYYAISKYSRTLAFPAGVCPTIGVGGNLAGGGFGMLLRKYG  IAAENVIDVKLVDANGKLHDKKSMGDDHFWAVRGGGGESFGIVVSWQVKLLPVPPTVTIF  KIPKSVSEGAVDIINKWQLVAPQLPADLMIRIIAMGPKATFEAMYLGTCKTLTPMMQSKF  PELGMNASHCNEMSWIESIPFVHLGHRDSLEGDLLNRNNTFKPFAEYKSDYVYEPFPKSV  WEQIFGTWLVKPGAGIMIFDPYGATISATPEAATPFPHRKGVLFNIQYVNYWFAPGAGAA  PLSWSKEIYNYMEPYVSKNPRQAYANYRDIDLGRNEVVNGVSTYSSGKVWGQKYFKGNFE  RLAITKGKVDPTDYFRNE---------- |
| Dac g 4.0101 | ------------------------------------------------------------  -----------------------DI---------------YNYMEP-YVSKVDPTDY---  FGNEQARTAWVDSGAQLGELSY--------------------------------------  ------------------------------------------------------------  ------------------------------------------------------------  ------------------------------------------------------------  ----------------------------------------GVLFNIQYVNYWFAP-----  ------------------------------------------------------------  ---------------------------- |

| **Grass pollen group 5 and 6 allergens** | |
| --- | --- |
| **Allergen** | **Aligned sequence** |
| Phl p 5.0101 | --MAVHQYTVALFLAVALVAGPAASYAADLGYGPATPAAPAAGYTPATPAAPAE-----A  APAGKATTEEQKLIEKINAGFKAALAAAAGVQPADKYRTFVATFGAAS--NKAFAEGLSG  EPKGAAE--SSSKAALTSKLDAAYKLAYKTAEGATPEAKYDAYVATLSEALRIIAGTLEV  HAVKPAAEEVKV--IPAGELQVIEKVDAAFKVAATAANAAPANDKFTVFEAAFNDAIKAS  TGGAYESYKFIPALEAAVKQAYAATVATAPEVKYTVFETALKKAITAMSEAQKAAKP---  ----------AAAATATATAAVGAATG------------------------AATAATGGY  KV |
| Phl p 5.0102 | ---------------------------ADLGYGPATPAAPAAGYTPATPAAPAG-----A  DAAGKATTEEQKLIEKINAGFKAALAG-AGVQPADKYRTFVATFGPAS--NKAFAEGLSG  EPKGAAE--SSSKAALTSKLDAAYKLAYKTAEGATPEAKYDAYVATLSEALRIIAGTLEV  HAVKPAAEEVKV--IPAGELQVIEKVDAAFKVAATAANAAPANDKFTVFEAAFNDEIKAS  TGGAYESYKFIPALEAAVKQAYAATVATAPEVKYTVFETALKKAITAMSEAQKAAKP---  ----------AAAATATATAAVGAATG------------------------AATAATGGY  KV |
| Phl p 5.0103 | --MAVHQYTVALFLAVALVAGPAGSYAADLGYGPATPAAPAAGYTPATPAAPAG-----A  EPAGKATTEEQKLIEKINAGFKAALAAAAGVPPADKYRTFVATFGAAS--NKAFAEGLSG  EPKGAAE--SSSKAALTSKLDAAYKLAYKTAEGATPEAKYDAYVATVSEALRIIAGTLEV  HAVKPAAEEVKV--IPAGELQVIEKVDAAFKVAATAANAAPANDKFTVFEAAFNDAIKAS  TGGAYESYKFIPALEAAVKQAYAATVATAPEVKYTVFETALKKAITAMSEAQKAAKP---  ----------AAAATATATAAVGAATG------------------------AATAATGGY  KV |
| Phl p 5.0104 | ---------------------------ADLGYG-----------GPATPAAPAE-----A  APAGKATTEEQKLIEKINDGFKAALAAAAGVPPADKYKTFVATFGAAS--NKAFAEGLSA  EPKGAAE--SSSKGALTSKLEAAYKLAYKTSEGATPEAKYDAYVATLSEALRIIAGTLEV  HAVKPAAEEVKV--IPAGELQFIEKVDSALKVAATAANAAAANDKFTVFEAAFNHAIKAS  TGGAYESYKFIPALEAAVKQAYAATVATAPEVKYTVFETALKKAITAMSEAQKAAKP---  ----------ATEATATATAAVGAATG------------------------AATAATGGY  KV |
| Phl p 5.0105 | ---------------------------ADLGYG-----------GPATPAAPAE-----A  APAGKATTEEQKLIEKINDGFKAALAAAAGVPPADKYKTFVATFGAAS--NKAFAEGLSA  EPKGAAE--SSSKAALTSKLDAAYKLAYKTAEGATPEAEYDAYVATLSEALRIIAGTLEV  HAVKPAAEEVKV--IPAGELQVIEKVDSALKVAATAANAAPANDKFTVFEAAFNNAIKAS  TGGAYESYKFIPALEAAVKQAYAATVATAPEVKYTVFETALKKAITATSEAQKAAKP---  ----------ATEATATATAAVGAATG------------------------AATAATGGY  KV |
| Phl p 5.0106 | ---------------------------ADLGYG-----------GPATPAAPAE-----A  APAGKATTEEQKLIEKINDGFKAALAAAAGVPPADKYKTFVATFGAAS--NKAFAEGLSA  EPKGAAE--SSSKAALTSKLDAAYKLAYKTAEGATPEAKYDAYVATLSEALRIIAGTLEV  HAVKPAAEEVKV--IPAGELQVIEKVDSAFKVAATAANAAPANDKFTVFEAAFNNAIKAS  TGGAYESYKFIPALEAAVKQAYAATVATAPEVKYTVFETALKKAITAMSEAQKAAKP---  ----------ATEATATATAAVGAATG------------------------AATAATGGY  KV |
| Phl p 5.0107 | ---------------------------ADLGYG-----------GPATPAAPAE-----A  APAGKATTEEQKLIEKINDGFKAALAAAAGVPPADKYKTFVATFGAAS--NKAFAEGLSA  EPKGAAE--SSSKAALTSKLDAAYKLAYKTAEGATPEAKYDAYVATLSEALRIIAGTLEV  HAVKPAAEEVKV--IPAGELQVIEKVDSAFKVAATAANAAPANDKFTVFEAAFNNAIKAS  TGGAYESYKFIPALEAAVKQAYAATVATAPEVKYTVFETALKKAITAMSEAQKAAKP---  ----------AAAATATATSAVGAATG------------------------ATTAAAGGY  KV |
| Phl p 5.0108 | ---------------------------ADLGYG-----------GPATPAAPAE-----A  APAGKATTEEQKLIEKINDGFKAALAAAAGVPPADKYKTFVATFGAAS--NKAFAEGLSA  EPKGAAE--SSSKAALTSKLDAAYKLAYKTAEGATPEAKYDAYVATLSEALRIIAGTLEV  HAVKPAAEEVKV--IPAGELQVIEKVDSAFKVAATAANAAPANDKFTVFEAAFNNAIKAS  TGGAYESYKFIPALEAAVKQAYAATVATAPEVKYTVFETALKKAFTAMSEAQKAAKP---  ----------ATEATATATAAVGAATG------------------------AATAATGGY  KV |
| Phl p 5.0109 | ---------------------------ADLGYGPATPAAPAAGYT---PAAPAG-----A  EPAGKATTEEQKLIEKINAGFKAALAAAAGVPPADKYRTFVATFGAAS--NKAFAEGLSG  EPKGAAE--SSSKAALTSKLDAAYKLAYKTAEGATPEAKYDAYVATLSEALRIIAGTLEV  HAVKPAAEEVKV--IPAGELQVIEKVDAAFKVAATAANAAPANDKFTVFEAAFNNAIKAS  TGGAYESYKFIPALEAAVKQAYAATVATAPEVKYTVFETALKKAITAMSEAQKAAKP---  ----------AAAATATATSAVGAATG------------------------AATAATGGY  KV |
| Phl p 5.0201 | --------------AAAAVPRRGPRGGPGRS------YTADAGYAPATPAAAGA------  AAGKAT-TEEQKLIEDINVGFKAAVAAAASVPAADKFKTFEAAFTSSS-----------K  AAAAK-------APGLVPKLDAAYSVAYKAAVGATPEAKFDSFVASLTEALRVIAGALEV  HAVKPVTEEPGMAKIPAGELQIIDKIDAAFKVAATAAATAPADDKFTVFEAAFNKAIKES  TGGAYDTYKCIPSLEAAVKQAYAATVAAAPQVKYAVFEAALTKAITAMSEVQKVSQP---  ----------ATGAATVAAGAATTAAGAAS--------------------GAATVAAGGY  KV |
| Phl p 5.0202 | -----------------AVPRRGPRGGPGRS------YAADAGYAPATPAAAGA------  EAGKAT-TEEQKLIEDINVGFKAAVAAAASVPAGDKFKTFEAAFTSSS-----------K  AATAK-------APGLVPKLDAAYSVAYKAAVGATPEAKFDSFVASLTEALRVIAGALEV  HAVKPVTEEPGMAKIPAGELQIIDKIDAAFKVAATAAATAPADDKFTVFEAAFNKAIKES  TGGAYDTYKCIPSLEAAVKQAYAATVAAAPQVKYAVFEAALTKAITAMSEVQKVSQP---  ----------ATGAATVAAGAATTATGAAS--------------------GAATVAAGGY  KV |
| Phl p 5.0203 | ---SVKRSNGSAEVHRGAVPRRGPRGGPGRS------YAADAGYAPATPAAAGA------  EAGKAT-TEEQKLIEDINVGFKAAVAAAASVPAADKFKTFEAAFTSSS-----------K  AATAK-------APGLVPKLDAAYSVAYKAAVGATPEAKFDSFVASLTEALRVIAGALEV  HAVKPVTEEPGMAKIPAGELQIIDKIDAAFKVAATAAATAPADDKFTVFEAAFNKAIKES  TGGAYDTYKCIPSLEAAVKQAYAATVAAAPQVKYAVFEAALTKAITAMSEVQKVSQP---  ----------ATGAATVAAGAATTAAGAAS--------------------GAATVAAGGY  KV |
| Phl p 5.0204 | ---------------------------------------ADAGYAPATPAAAGA------  AAGKAT-TEEQKLIEDINVGFKAAVAAAASVPAADKFKTFEAAFTSSS-----------K  AATAK-------APGLVPKLDAAYSVAYKAAVGATPEAKFDSFVASLTEALRVIAGALEV  HAVKPVTEEPGMAKIPAGELQIIDKIDAAFKVAATAAATAPADDKFTVFEAAFNKAIKES  TGGAYDTYKCIPSLEAAVKQAYAATVAAAPQVKYAVFEAALTKAITAMSEVQKVSQP---  ----------ATGAATVAAGAATTAAGAAS--------------------GAATVAAGGY  KV |
| Phl p 5.0205 | ---------------------------------------ADAGYAPATPAAAGA------  EAGKAT-TEEQKLIEDINVGFNAAVAAAASVPAADKFKTFEAAFTSSS-----------K  AATAK-------APGLVPKLDAAYSVAYKAAVGATPEAKFDSLVASLTEALRVIAGALEV  HAVKPVTEEPGMAKIPAGELQIIDKIDAAFKVAATAAATAPADDKFTVFEAAFNKAIKES  TGGAYDTYKCIPSLEAAVKQPYAATVAAAPQVKYAVFEAALTKAITAMSEVQKVSQP---  ----------ATGAATVAAGAATTAAGAAS--------------------GAATVAAGGY  KV |
| Phl p 5.0206 | --------MAVQKYTVALFLAVALVAGPAAS------YAADAGYAPATPAAAGA------  EAGKAT-TEEQKLIEDINVGFKAAVAAAASVPAADKFKTFEAAFTSSS-----------K  AATAK-------APGLVPKLDAAYSVAYKAAVGATPEAKFDSFVASLTEALRVIAGALEV  HAVKPVTEDPAWPKIPAGELQIIDKIDAAFKVAATAAATAPADDKFTVFEAAFNKAIKES  TGGAYDTYKCIPSLEAAVKQAYAATVAAAPQVKYAVFEAALTKAITAMSEVQKVSQP---  ----------ATGAATVAAGAATTATGAAS--------------------GAATVAAGGY  KV |
| Phl p 5.0207 | --------MAVQKYTVALFLAVALVAGPAAS------YAADAGYAPATPAAAGA------  EAGKAT-TEEQKLIEDINVGFKAAVAAAASVPAADKFKTFEAAFTSSS-----------K  AATAK-------APGLVPKLDAAYSVSYKAAVGATPEAKFDSFVASLTEALRVIAGALEV  HAVKPVTEEPGMAKIPAGELQIIDKIDAAFKVAATAAATAPAD---TVFEAAFNKAIKES  TGGAYDTYKCIPSLEAAVKQAYAATVAAAPQVKYAVFEAALTKAITAMSEVQKVSQP---  ----------ATGAATVAAGAATTAAGAAS--------------------GAATVAAGGY  KV |
| Phl p 6.0101 | --------------MVAMFLAVAVVLG---------------------LATSPT------  AEGGKATTEEQKLIEDVNASFRAAMATTANVPPADKYKTFEAAFTVSS-----------K  RNLAD---AVSKAPQLVPKLDEVYNAAYNAADHAAPEDKYEAFVLHFSEALRIIAGTPEV  HAVKPGA-----------------------------------------------------  ------------------------------------------------------------  ------------------------------------------------------------  -- |
| Phl p 6.0102 | --------MAAHKFMVAMFLAVAVVLG---------------------LATSPT------  AEGGKATTEEQKLIEDINASFRAAMATTANVPPADKYKTFEAAFTVSS-----------K  RNLAD---AVSKAPQLVPKLDEVYNAAYNAADHAAPEDKYEAFVLHFSEALHIIAGTPEV  HAVKPGA-----------------------------------------------------  ------------------------------------------------------------  ------------------------------------------------------------  -- |

| Lol p 5.0101 | --MAVQKHTVALFLAVALVAGPAASYAADAGYAPATPATPAAPATAATPATPATPATPAA  VPSGKATTEEQKLIEKINAGFKAAVAAAAVVPPADKYKTFVETFGTAT--NKAFVEGLAS  ---GYAD--QS-KNQLTSKLDAALKLAYEAAQGATPEAKYDAYVATLTEALRVIAGTLEV  HAVKPAAEEVKVGAIPAAEVQLIDKVDAAYRTAATAANAAPANDKFTVFENTFNNAIKVS  LGAAYDSYKFIPTLVAAVKQAYAAKQATAPEVKYTVSETALKKAVTAMSEAEKEATP---  ----------AAAATATPTPAAATATATPAAAYATATPAAATATATPAAATATPAAAGGY  KV |
| --- | --- |
| Lol p 5.0102 | --------MAVQKYTVALFLAVALVAGPAASYAADAGYTPAAAATPATPAATPA------  AAGGKATTDEQKLLEDVNAGFKAAVAAAANAPPADKFKIFEAAFSESS-----------K  GLLAT---SAAKAPGLIPKLDTAYDVAYKAAE-ATPEAKYDAFVTALTEALRVIAGALEV  HAVKPATEEVLAAKIPTGELQIVDKIDAAFKIAATAANAAPTNDKFTVFESAFNKALNEC  TGGAYETYKFIPSLEAAVKQAYAATVAAAPEVKYAVFEAALTKAITAMTQAQKAGKPAAA  AATAAATVATAAATAAAVLPPPLLVVQSLISLLIYY------------------------  -- |
| Hol l 5.0101 | -----------------------------------------ADAGYTPAAPAAA------  GAGGKATTDEQKLLEDVNAGFKTAVAAAANVPPADKYKTFEAAFTASS-----------K  ASIA---AAATKAPGLIPQLNAATNTAYAAAQGATPEAKYDAFVTTLTEALRVIAGALEV  HAVKPATEEVGAAKIPAGELQIVDKIDAAFRIAATAANAAPVNDKFTVFEGAFNKAIKES  TGGAYEAYKFIPSLETAVKQAYAATVATAPEVKYTVFETALKKAITAMSEAQKEAKP---  ----------VAAATGAATAAAGVAAGAAT------------------------AAAGGY  KV |
| Hol l 5.0201 | ------------------------------------------------------------  ----------QKLLEDVNASFKAAVAAAAKVPPADKYKTFLRAFTVLD-----------R  GSTEQSKAEETKMPELSSKLVDAYMAAFKASTGGTQEAKYDAFVTTLTEALRVIAGALEV  HAVKPATEEVPAAKIPAGDLQVVDKIDASFKIAATAANAAPANDKFTVFETAFNKALKES  TGGAYESYKFIPSLEAAVKQAYASTVAAAPEVKYAVFEAALTKAITAMSQAQKVAQP---  ----------AAAATGAAT----VAAGAAT------------------------TAAGGY  KV |
| Hor v 5.0101 | MANSGREHSAVPRRRNLVALVPRHGCYAEFSLYVCVGNINAPFPVFNRTTFIAN-----A  GIEAELEPHFLLLLFTFSSSSSFFTLLKTMIHFTDRSDNKNKAMMRGREFRKAFAEVLKG  AATGQIAGQSSSMAKLSSSLELSYKLAYDKAQGATPEAKYDAYVATLTESLRVISGTLEV  HSVKPAAEEVKG--VPAGELKAIDQVDAAFRTAATAADAAPANDKFTVFESLQQGPSRKP  RGGAYESYKFIPALEAAVKQAYAATVAAAPEVKFTVFQTALSKAINAMTQAGKVAKP---  ----------AAAA----TATATVAAG-------------------------AAATAGNY  KV |
| Pha a 5.0101 | --------MAVQKYTMALFLAVALVAGPAAP----------TPPTPRTPPLLPP------  PRARDKATLTSRSVEDINAASRR--PWWASVPPADKFKTFADHVLCVP-----------N  ADVTS---AATKAPQLKAKLDAAYRVAYEAAEGSTPEAKYDAFIAALTEALRVIAGAFEV  HAVKPATEEVVAD--PVGELQIVDKIDAAFKIAATAANSAPANDKFTVFEGAFNKAIKES  TAGAYETYKFIPSLEAAVKQAYGATVARAPEVKYAVFEAGLTKAITAMSEAQKVAKPPLS  PQPPQVLPLAAGGAATVAAASDVRVCRSHGTLQDACLLRCRGGCQPVVWRGGSHRARGGY  KV |
| Poa p 5.0101 | --------MAVQKYTVALFLTVALVAGPAAS------YAADAGYAPATPAAAGA------  AAGKITPTQEQKLMEDINVGFKAAVAAAAGAPPADKFKTFQAAFSASV-----------E  ASAAKL--NAAQAPGFVSHVAATSDATYKAAVGATPEAKFDSFVAAFTEALRIIAGVLKV  HAVKPITEETGAAKIPAGEQQIIDKIDAAFKVAATAANAAPANDKFTVFEAAFNNAIKES  TGGAYDTYKSIPSLEAAVKQAYAATIAAAPEVKFAVFKAALTKAITAMAEVQKVSKP---  ----------VAGAATVAAGAATAATGAATGAAGAA-------------TGAATVSAGGY  KV |
| Sec-c-5.0101 | --------MAVQQYT--------------VALFLAVALVAGPAVSYG-TYAPAA-----P  GTQPKATTPEQKLMENINNGFKAAVEAAAAVAPADKYKTFQTTFIKGS--NKAFADVLTA  AASGQIPAQSDSMARLSTSLESSYKLAYDSAEGATPETKYDTYVASLTESLRVISGAFEV  HAVKPASEEVKG--VPAPQLKVVDQIDAAYRTAATAANAAPTNDKFNVFESSFNKAIKEN  TGGAYASYTFVPALESAVKQAYAATVASAPEVKYAVFQAALSKAINAMVEAEKDAKP---  ----------AAAAAATATATATVGAA-------------------------AGAAAGGY  KA |
| **Grass pollen group 7 allergens** | |
| **Allergen** | **Aligned sequence** |
| Phl p 7.0101 | MADDMERIFKRFDTNGDGKISLSELTDALRTLGSTSADEVQRMMAEIDTDGDGFIDFNEF  ISFCNANPGLMKDVAKVF |

| **Grass pollen group 11 allergens** | |
| --- | --- |
| **Allergen** | **Aligned sequence** |
| Phl p 11.0101 | DKGPGFVVTGRVYCDPCRAGFETNVSHNVQGATVAVDCRPFNGGESKLKAEATTDGLGWY  KIEIDQDHQEEICEVVLAKSPDTTCSEIEEFRDRARVPLTSNNGIKQQGIRYANPIAFFR  KEPLKECGGILQAYDLRDAPETP |
| Lol p 11.0101 | DKGPGFVVTGRVYCDPCRAGFETNVSHNVEGATVAVDCRPFDGGESKLKAEATTDKDGWY  KIEIDQDHQEEICEVVLAKSPDKSCSEIEEFRDRARVPLTSNXGIKQQGIRYANPIAFFR  KEPLKECGGILQAY--------- |
| **Grass pollen group 12 allergens** | |
| **Allergen** | **Aligned sequence** |
| Phl p 12.0101 | MSWQTYVDEHLMCEIEGHHLASAAILGHDGTVWAQSADFPQFKPEEITGIMKDFDEPGHL  APTGMFVAGAKYMVIQGEPGRVIRGKKGAGGITIKKTGQALVVGIYDEPMTPGQCNMVVE  RLGDYLVEQGM |
| Phl p 12.0102 | MSWQTYVDEHLMCEIEGHHLASAAILGHDGTVWAQSADFPQFKPEEITGIMKDFDEPGHL  APTGMFVAGAKYMVIQGEPGAVIRGKKGAGGITIKKTGQALVVGIYDEPMTPGQCNMVVE  RLGDYLVEQGM |
| Phl p 12.0103 | MSWQTYVDEHLMCEIEGHHLASAAIFGHDGTVWAQSADFPQFKPEEITGIMKDLDEPGHL  APTGMFVAAAKYMVIQGEPGAVIRGKKGAGGITIKKTGQALVVGIYDEPMTPGQCNMVVE  RLGDYLVEQGM |
| Hor v 12.0101 | MSWQTYVDDHLCCEIDGQHLTSAAILGHDGRVWVQSPNFPQFKPEEIAGIIKDFDEPGHL  APTGLFLGGTKYMVIQGEPGVVIRGKKGTGGITIKKTGMPLILGIYDEPMTPGQCNLVVE  RLGDYLVEQGF |
| **Grass pollen group 13 allergens** | |
| **Allergen** | **Aligned sequence** |
| Phl p 13.0101 | GKKEEKKEEKKESGDAASGADGTYDITKLGAKPDGKTDCTKEVEEAWASACGGTGKNTIV  IPKGDFLTGPLNFTGPCKGDSVTIKLDGNLLSSNDLAKYKANWIEIMRIKKLTITGKGTL  DGQGKAVWGKNSCAKNYNCKILPNTLVLDFCDDALIEGITLLNAKFFHMNIYECKGVTVK  DVTITAPGDSPNTDGIHIGDSSKVTITDTTIGTGDDCISIGPGSTGLNITGVTCGPGHGI  SVGSLGRYKDEKDVTDITVKNCVLKKSTNGLRIKSYEDAKSPLTASKLTYENVKMEDVGY  PIIIDQKYCPNKICTSKGDSARVTVKDVTFRNITGTSSTPEAVSLLCSDKQPCNGVTMND  VKIEYSGTNNKTMAVCTNAKVTAKGVSEANTCAA |
| **Acidic ribosomal protein P1 allergens** | |
| **Allergen** | **Aligned sequence** |
| Alt a 12.0101 | -MSTSELATSYAALILADDGVDITADKLQSLIKAAKIEEVEPIWTTLFAKALEGKDVKDL  LLNVGSGGGAAPLPEALLLR----WRAADAAPAAEEKKEEEKEESDEDMGFGLFD |
| Cla h 12.0101 | -MSAAELASSYAALILADEGLEITADKLQALISAAKVPEIEPIWTSLFAKALEGKDVKDL  LLNVGSGGGAAPAAGGAAAG----GAAAVLDAPAEEKAEEEKEESDDDMGFGLFD |
| Pen b 26.0101 | -MSTAELAVSYAALILADDGIEVSADKIQTILGAAKVQEVEPIWATIFAKALEGKDIKEI  LTNVGSAGPATAGAPAAAG------AAAPAEEKKEEK-EEEKEESDEDMGFGLFD |
| Pen cr 26.0101 | -MSTAELACSYAALILADDGIEISADKIQTLISAANVQEVEPIWASIFARALEGKDIKEL  LTNVGSAGPASAAPAGAAG------AAAPAEEKAEEK-EEEKEESDEDMGFGLFD |
| Human 60S acidic ribosomal protein P1 | MASVSELACIYSALILHDDEVTVTEDKINALIKAAGVN-VEPFWPGLFAKALANVNIGSL  ICNVGAGGPAPAAGAAPAGGPAPSTAAAPAEEKKVEAKKEESEESDDDMGFGLFD |

| **Acidic ribosomal protein P2 allergens** | |
| --- | --- |
| **Allergen** | **Aligned sequence** |
| Alt a 5.0101 | MKHLAAYLLLGLGGNTSPSAADVKAVLESVGIEADSDRLDKLISELEGKDINELIASGSE  KLASVPSGGAGGAAASGGAAAAGGSAQAEAAPEAA--KEEEKEESDEDMGFGLFD |
| Asp f 8.0101 | MKYLAAFLLLALAGNTSPSSEDVKAVLSSVGIDADEERLNKLIAELEGKDLQELIAEGST  KLASVPSGGAAAAAPAAAGAAAGG--AAAPAAKEK--NEEEKEESDEDMGFGLFD |
| Cla h 5.0101 | MKYLAAFLLLGLAGNSSPSAEDIKTVLSSVGIDADEERLSSLLKELEGKDINELISSGSE  KLASVPSGGAGAA-SAGGAAAAGG--AAEAAPEAER-AEEEKEESDDDMGFGLFD |
| Fus c 1.0101 | MKHLAAYLLLGLGGNTSPSAADVKAVLTSVGIDADEDRLNKLISELEGKDIQQLIAEGSE  KLASVPSGGAGG--ASGGAAAAGG--AAEEAKEEE--KEEEKEESDEDMGFGLFD |
| Pru du 5.0101 | MKVVAAYLLAVLGGNTTPSAEDLKDILGSVGAETDDDRIQLLLSEVKGKDITELIASGRE  KLASVPSGGGAVAVAAPGAGAGAA--APAAAEPKKEEKVEEKEDTDDDMGFSLFD |
| Human 60S acidic ribosomal protein P2 | ﻿MRYVASYLLAALGGNSSPSAKDIKKILDSVGIEADDDRLNKVISELNGKNIEDVIAQGIG  KLASVPAGGAVAVSAAPGSAAPAAGSAPAAAEEKKDEKKEESEESDDDMGFGLFD |
