# Supplementary material for: Linear Epitope Binding Patterns of Grass Pollen-Specific Antibodies in Allergy and in Response to Allergen-Specific Immunotherapy
Source: Front Allergy. 2022 Mar 31;3:859126. doi: 10.3389/falgy.2022.859126 (PMC9234942; doi:10.3389/falgy.2022.859126)
Supplement: Supplementary file 1 [file Data_Sheet_1.ZIP › Supplementary Figure 1.pdf]

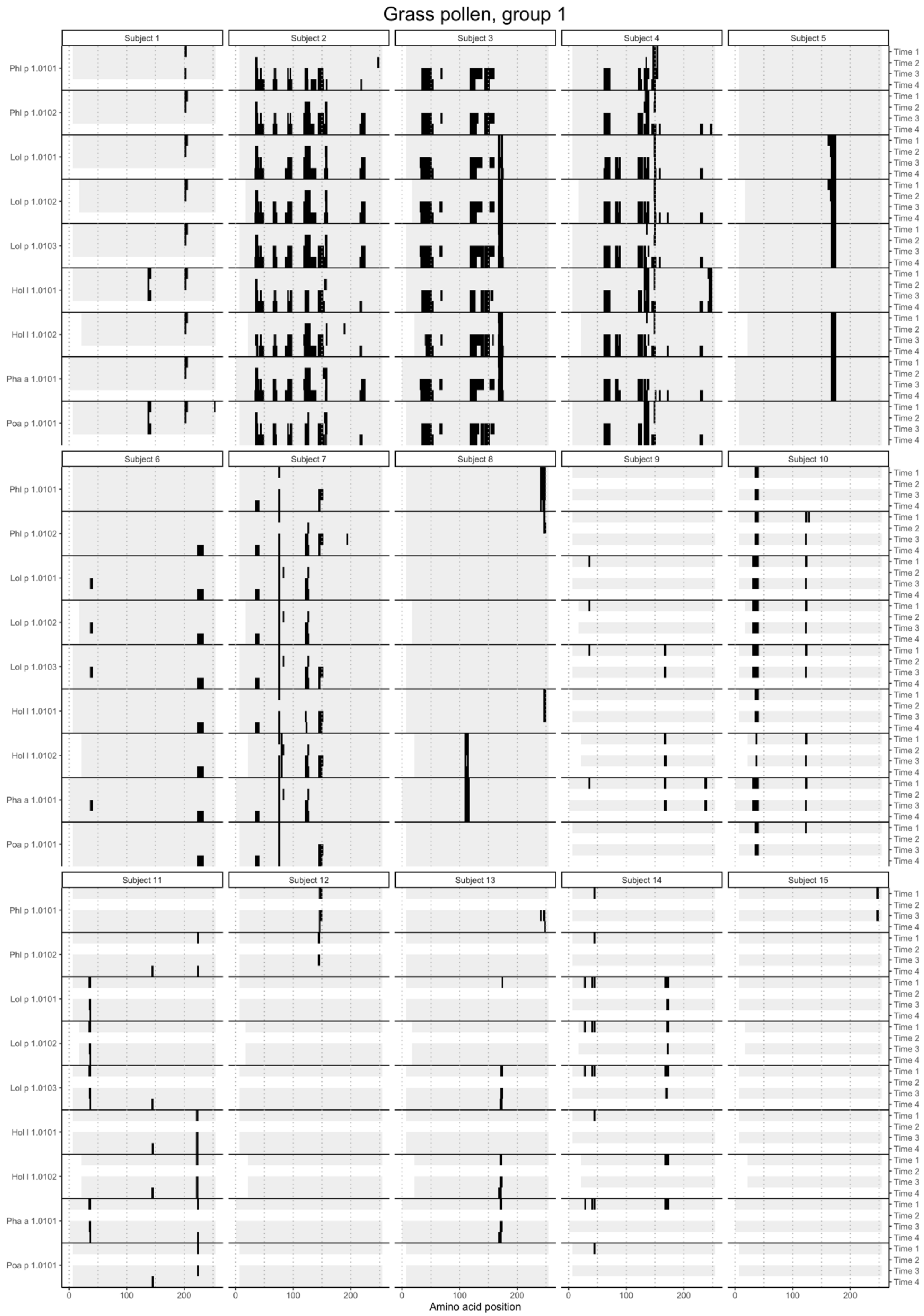

Grass pollen, group 1
Subject 1
Subject 2
Subject 3
Subject 4
Subject 5
Subject 6
Subject 7
Subject 8
Subject 9
Subject 10
Subject 11
Subject 12
Subject 13
Subject 14
Subject 15
Phl p 1.0101
Phl p 1.0102
Lol p 1.0101
Lol p 1.0102
Lol p 1.0103
Hol l 1.0101
Hol l 1.0102
Pha a 1.0101
Poa p 1.0101
Time 1
Time 2
Time 3
Time 4
0
100
200
Amino acid position

Grass pollen, group 2 and 3

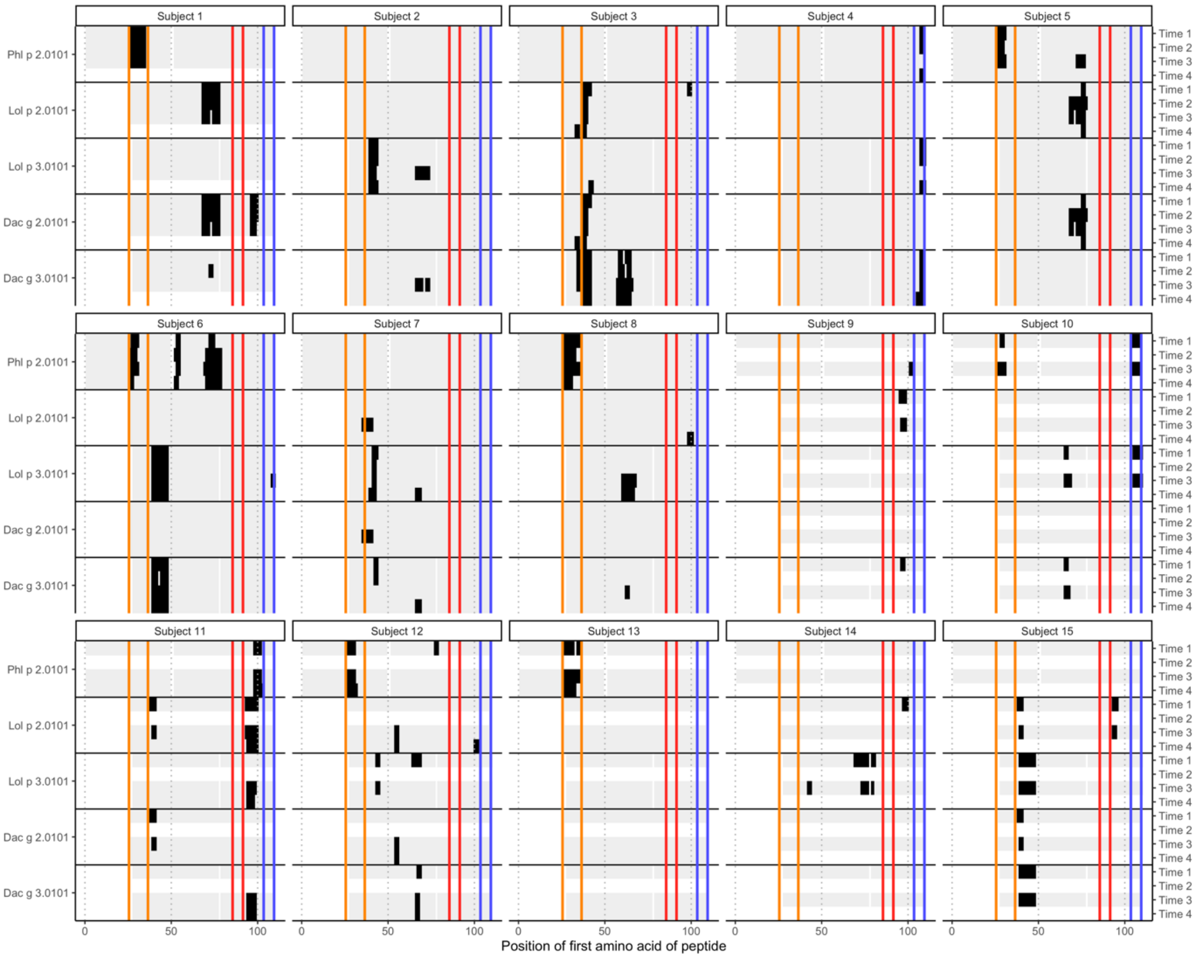

Grass pollen, group 4

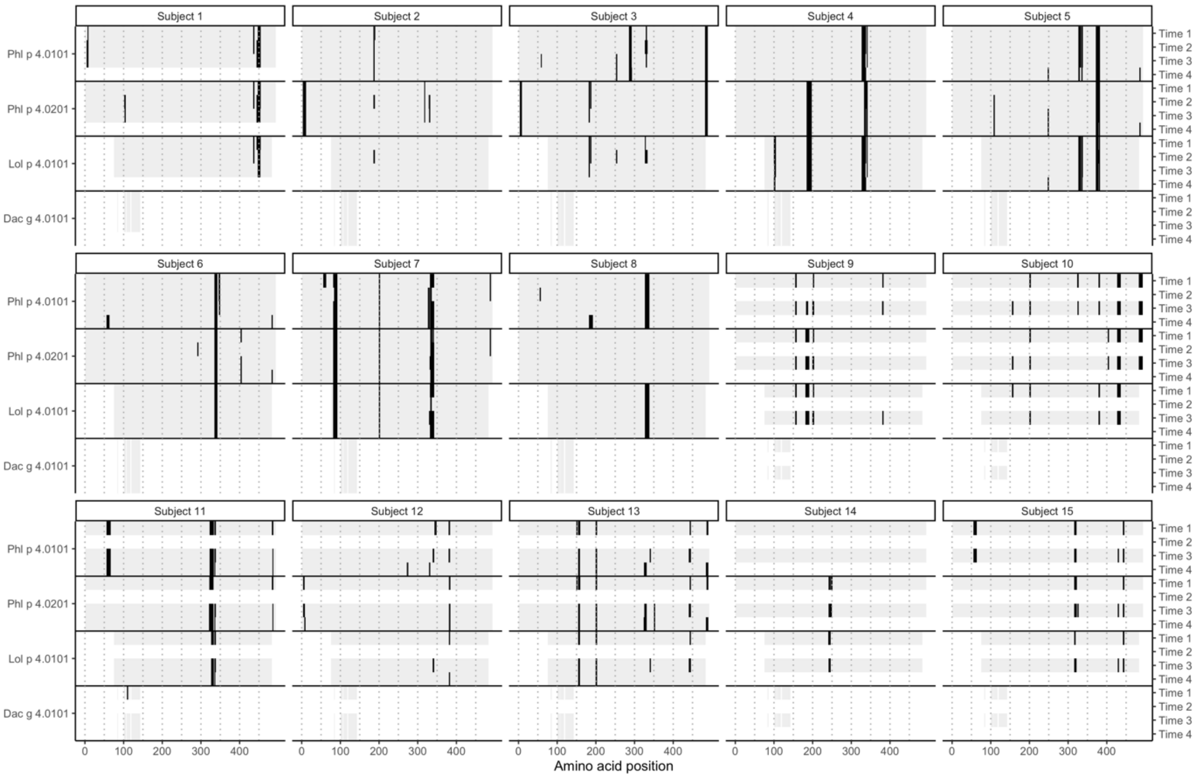

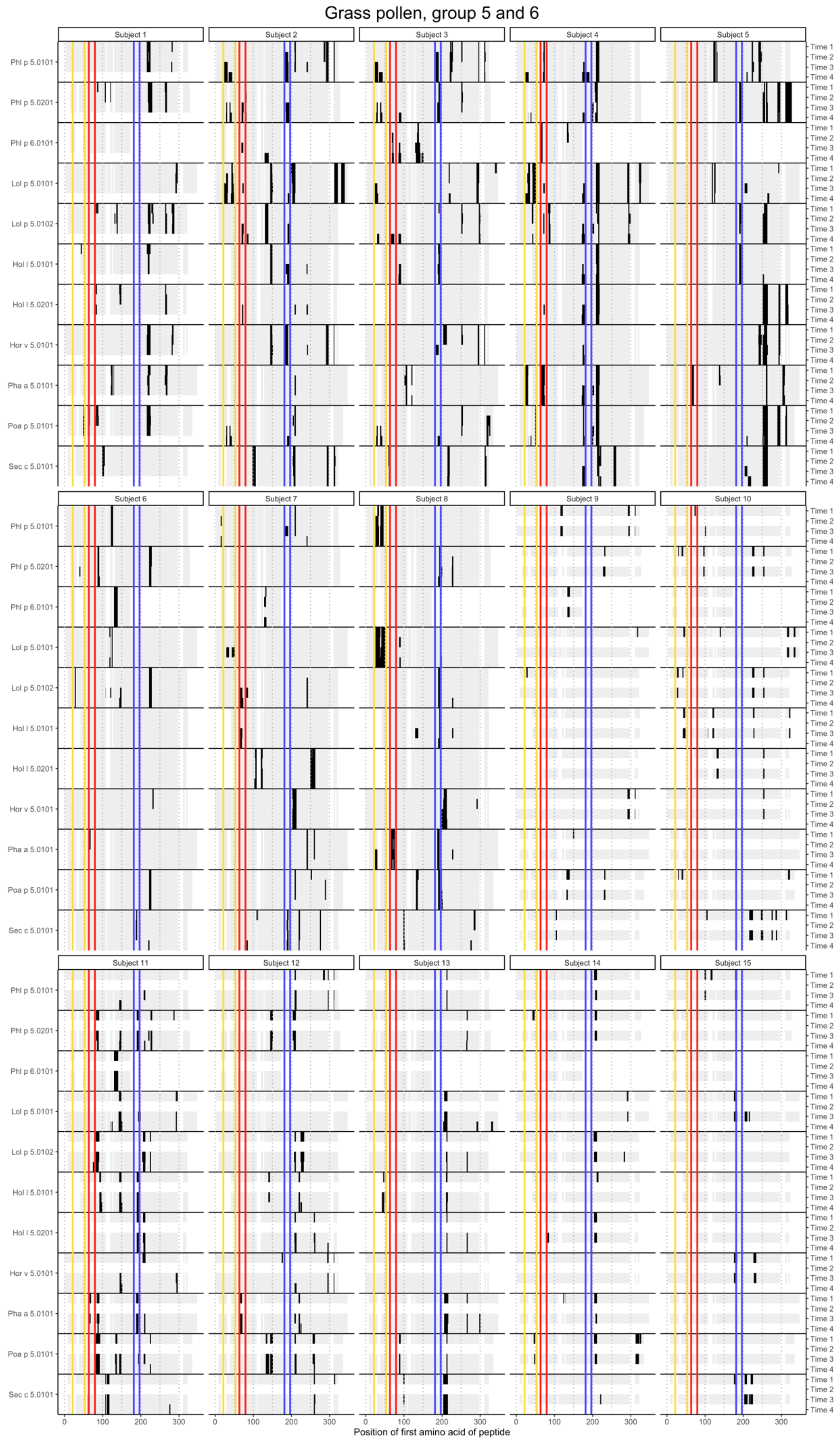

Grass pollen, group 5 and 6
Subject 1
Subject 2
Subject 3
Subject 4
Subject 5
Subject 6
Subject 7
Subject 8
Subject 9
Subject 10
Subject 11
Subject 12
Subject 13
Subject 14
Subject 15
Phl p 5.0101
Phl p 5.0201
Phl p 6.0101
Lol p 5.0101
Lol p 5.0102
Hol l 5.0101
Hol l 5.0201
Hor v 5.0101
Pha a 5.0101
Poa p 5.0101
Sec c 5.0101
Time 1
Time 2
Time 3
Time 4
0
100
200
300
Position of first amino acid of peptide

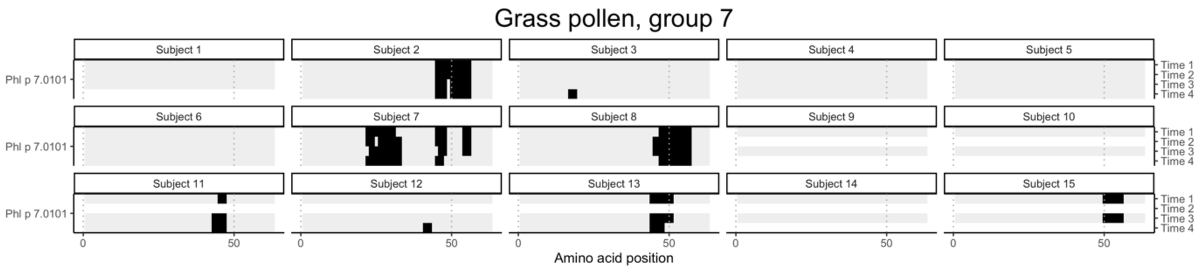
Grass pollen, group 7
Subject 1
Subject 2
Subject 3
Subject 4
Subject 5
Subject 6
Subject 7
Subject 8
Subject 9
Subject 10
Subject 11
Subject 12
Subject 13
Subject 14
Subject 15
Phl p 7.0101
Time 1
Time 2
Time 3
Time 4
0
50
Amino acid position

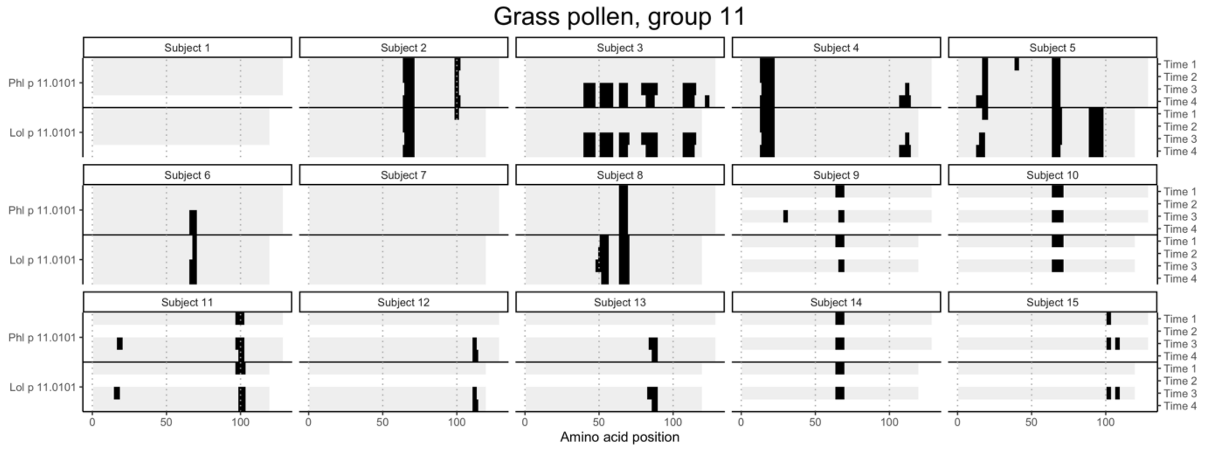
Grass pollen, group 11
Subject 1
Subject 2
Subject 3
Subject 4
Subject 5
Subject 6
Subject 7
Subject 8
Subject 9
Subject 10
Subject 11
Subject 12
Subject 13
Subject 14
Subject 15
Phl p 11.0101
Lol p 11.0101
Time 1
Time 2
Time 3
Time 4
0
50
100
Amino acid position

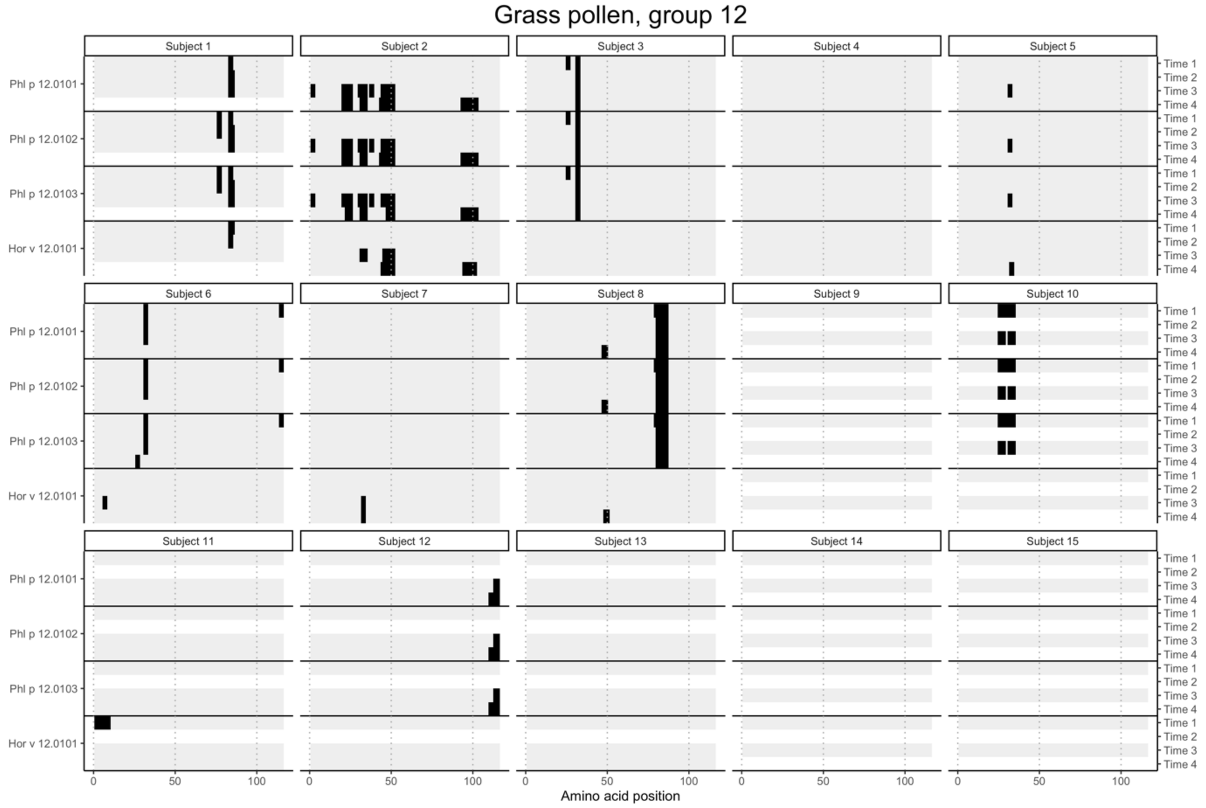
Grass pollen, group 12
Subject 1
Subject 2
Subject 3
Subject 4
Subject 5
Subject 6
Subject 7
Subject 8
Subject 9
Subject 10
Subject 11
Subject 12
Subject 13
Subject 14
Subject 15
Phl p 12.0101
Phl p 12.0102
Phl p 12.0103
Hor v 12.0101
Time 1
Time 2
Time 3
Time 4
0
50
100
Amino acid position

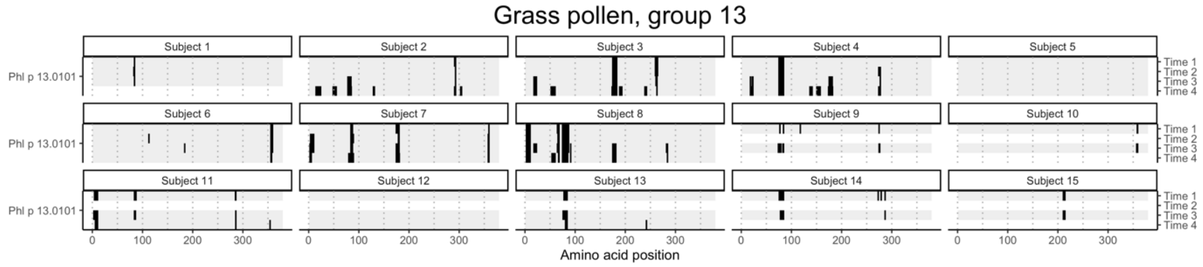

Supplementary Figure 1. Heatmap visualisation of IgG recognition of linear epitopes on grass pollen allergens, with reactive peptides in black and non-reactive peptides in grey. Epitope A (yellow), B (blue), and C (red) have been marked for group 5 and 6 grass pollen allergens and epitope D (orange), E (red), and F (blue) have been marked for group 2 and 3 grass pollen allergens.
