# Supplementary material for: Linear Epitope Binding Patterns of Grass Pollen-Specific Antibodies in Allergy and in Response to Allergen-Specific Immunotherapy
Source: Front Allergy. 2022 Mar 31;3:859126. doi: 10.3389/falgy.2022.859126 (PMC9234942; doi:10.3389/falgy.2022.859126)
Supplement: Supplementary file 1 [file Data_Sheet_1.ZIP › Supplementary Figure 6.pdf]

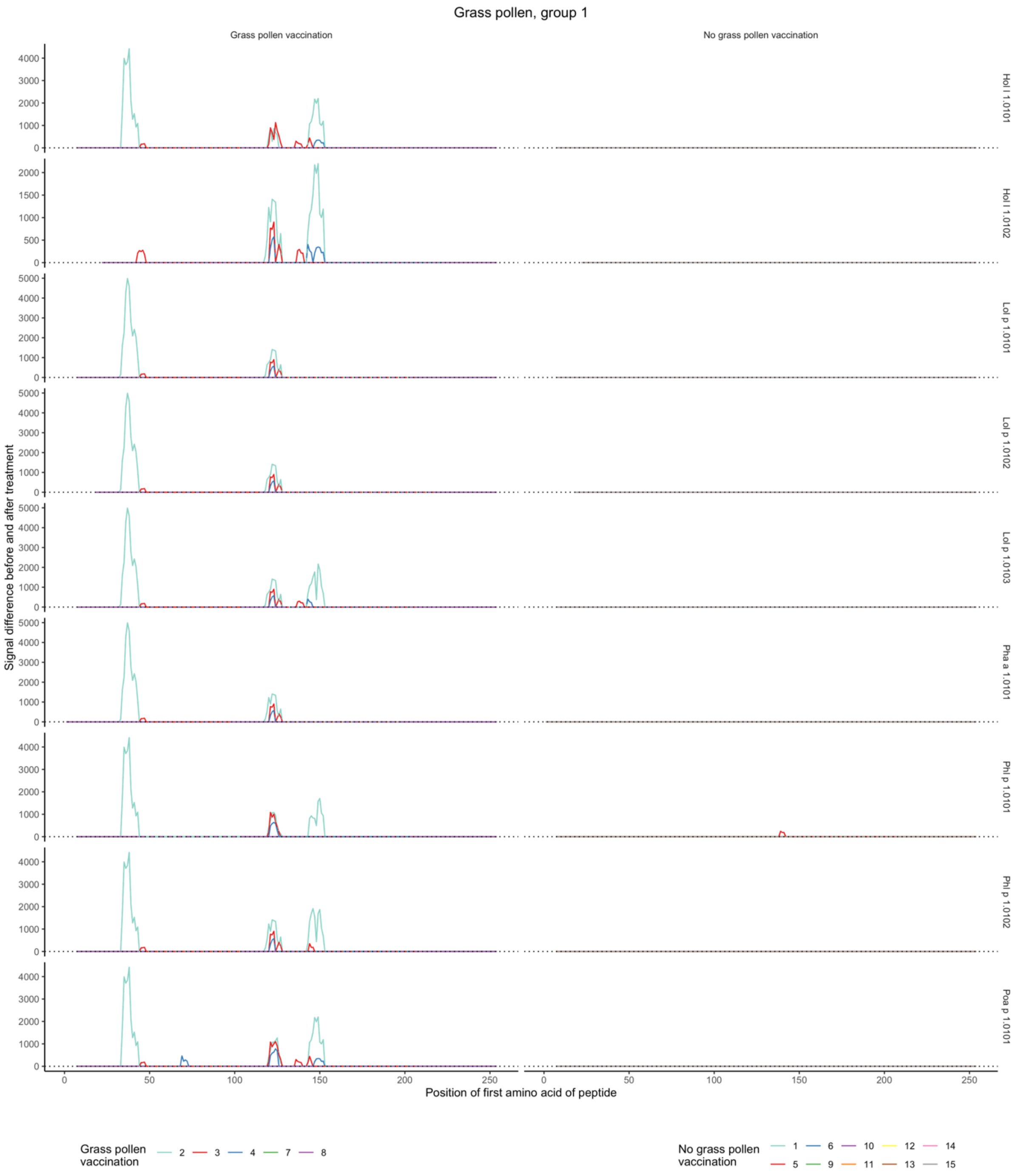

Mean number of peptides with
signal difference above 0:
14.2

Significance:
*

Mean number of peptides with
signal difference above 0:
0.3

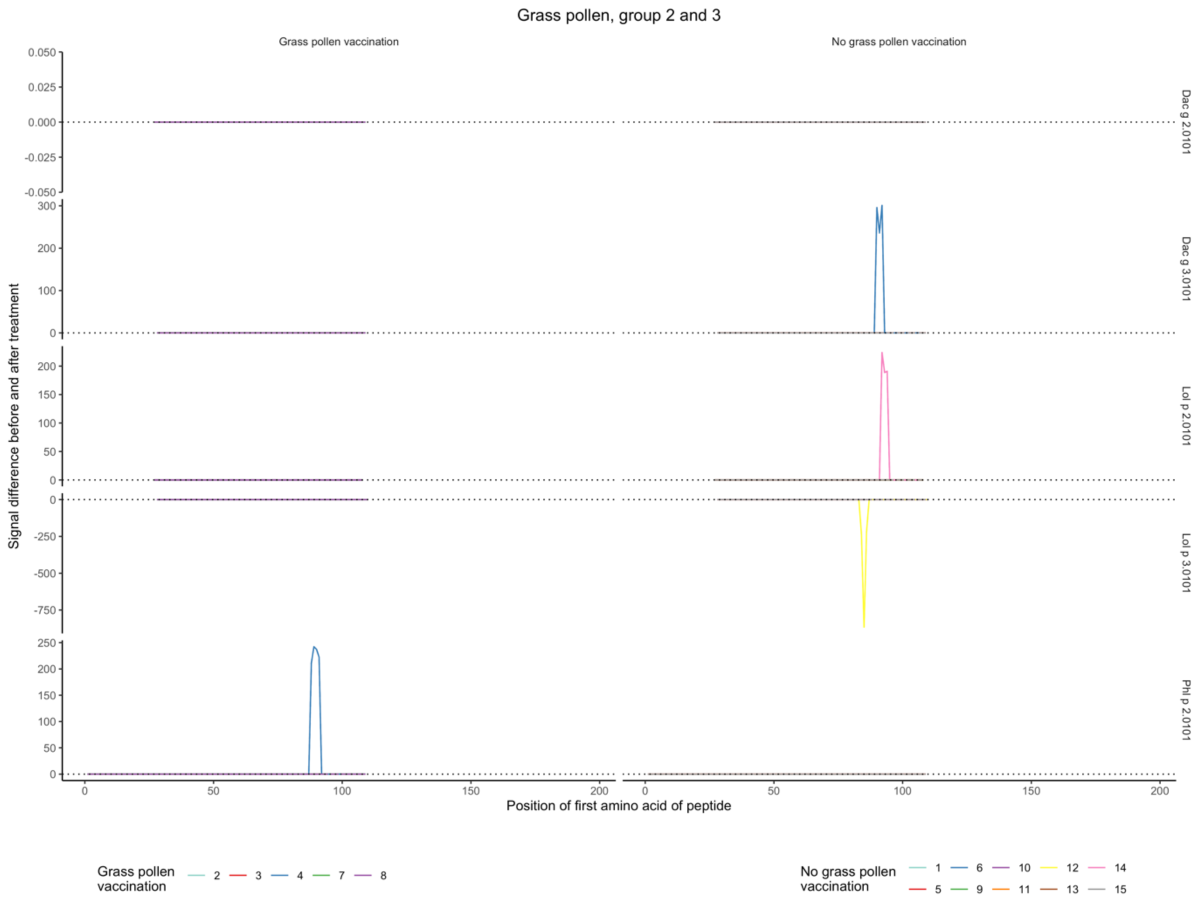

Mean number of peptides with signal difference above 0: 0.8

Significance: ns

Mean number of peptides with signal difference above 0: 0.6

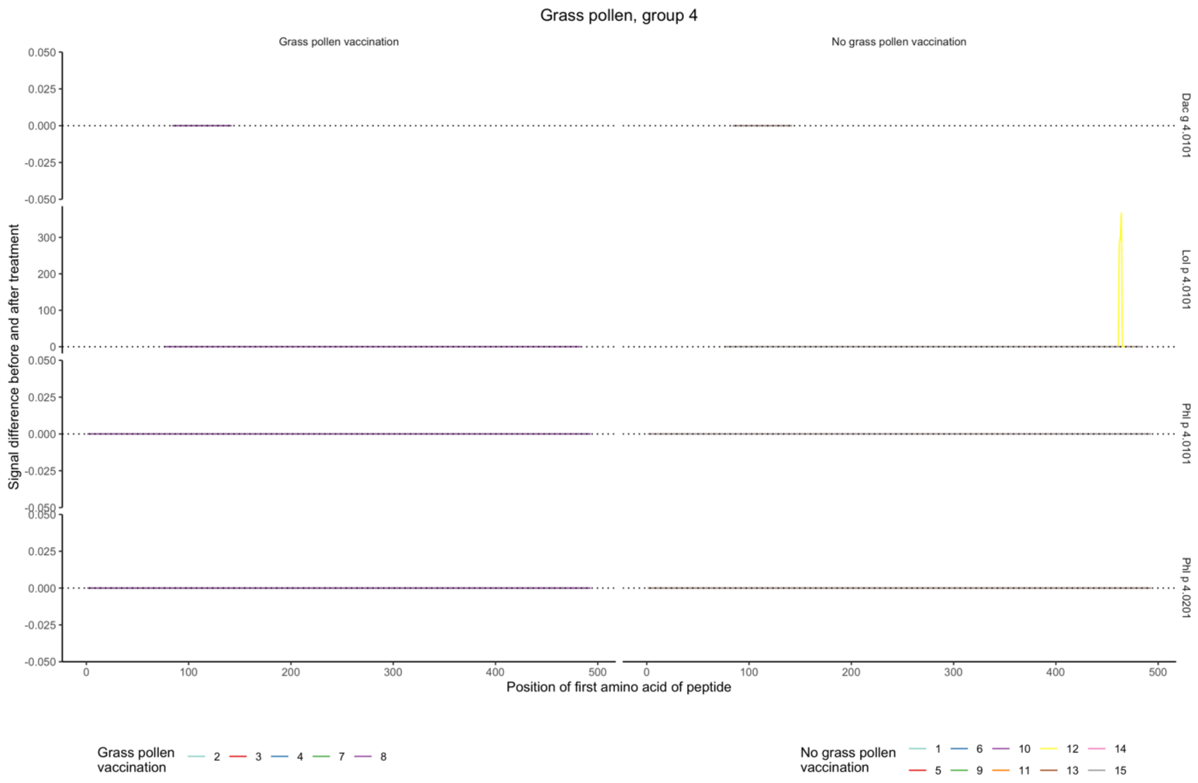

Mean number of peptides with signal difference above 0: 0

Significance: ns

Mean number of peptides with signal difference above 0: 0.3

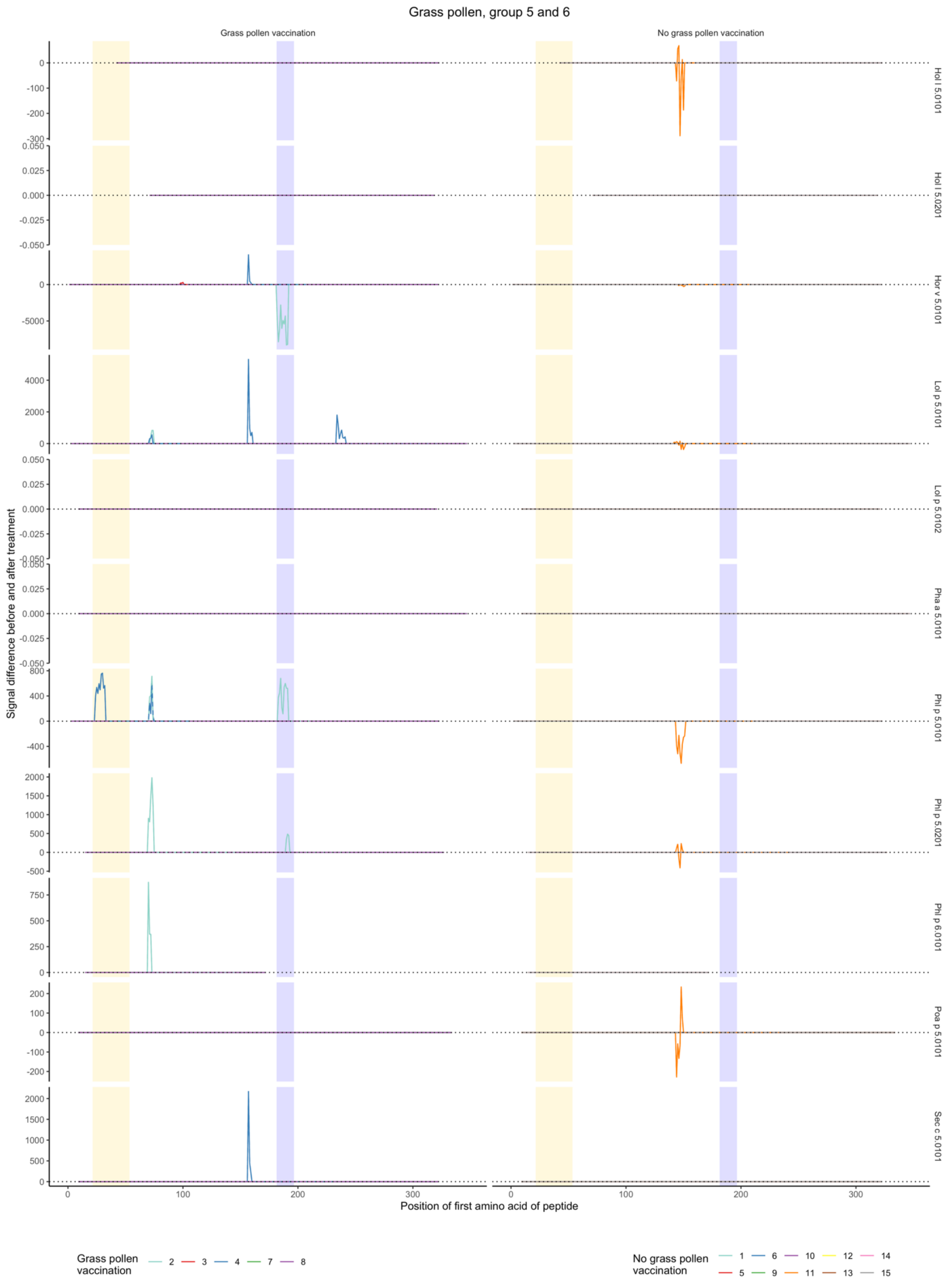

Mean number of peptides with signal difference above 0:
6.8

Significance:
*

Mean number of peptides with signal difference above 0:
0.2

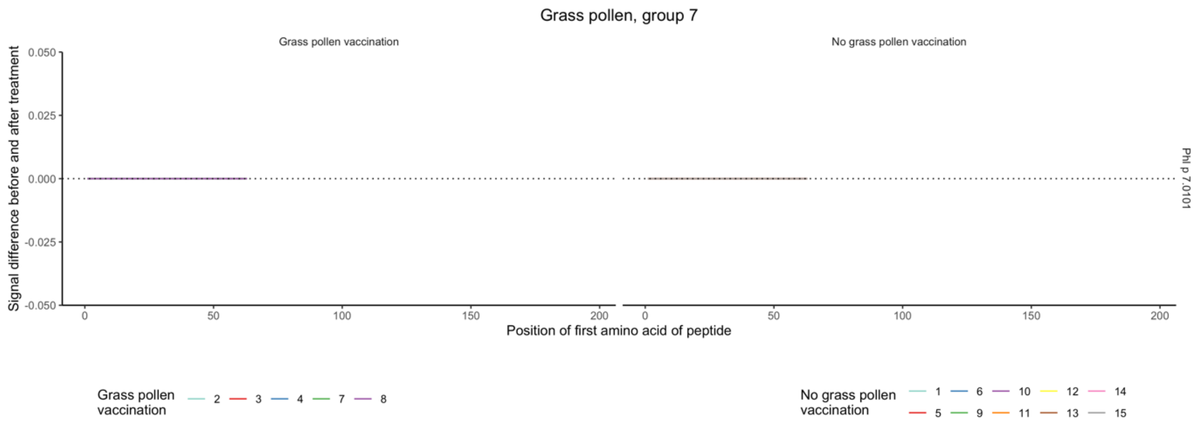

Mean number of peptides with signal difference above 0:
0

Significance:
ns

Mean number of peptides with signal difference above 0:
0

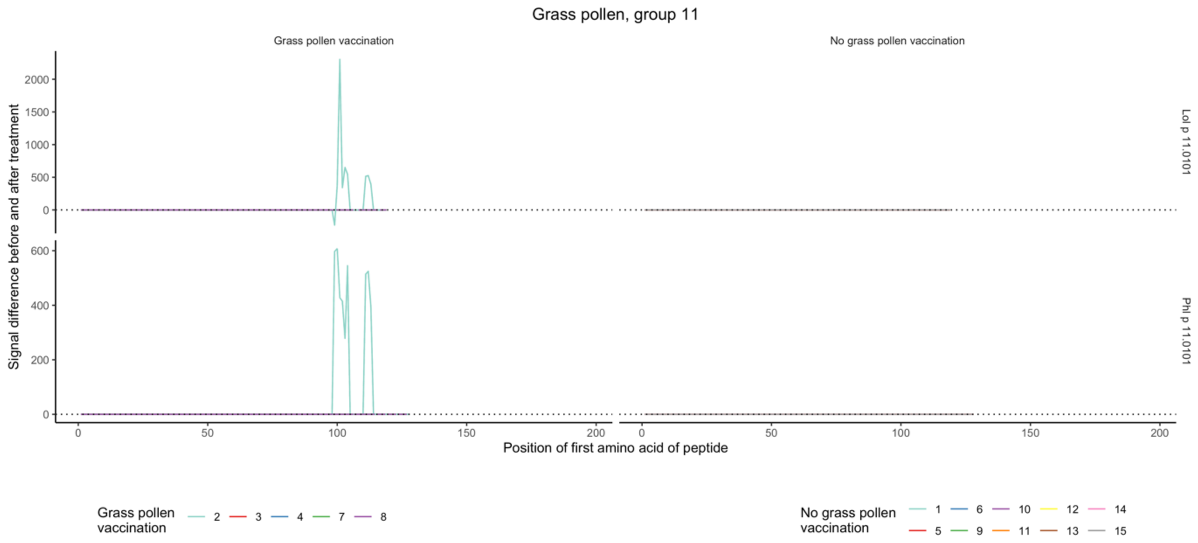

Mean number of peptides with signal difference above 0:
1.8

Significance:
ns

Mean number of peptides with signal difference above 0:
0

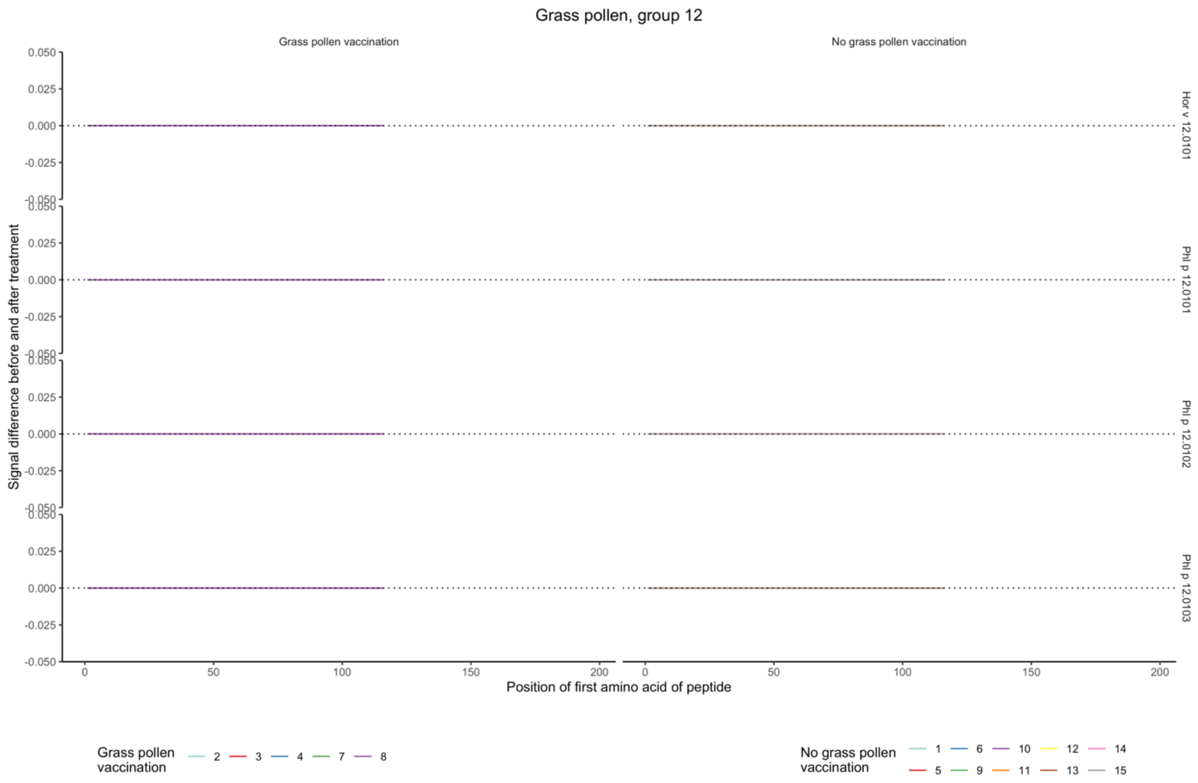

Mean number of peptides with signal difference above 0:
0

Significance:
ns

Mean number of peptides with signal difference above 0:
0

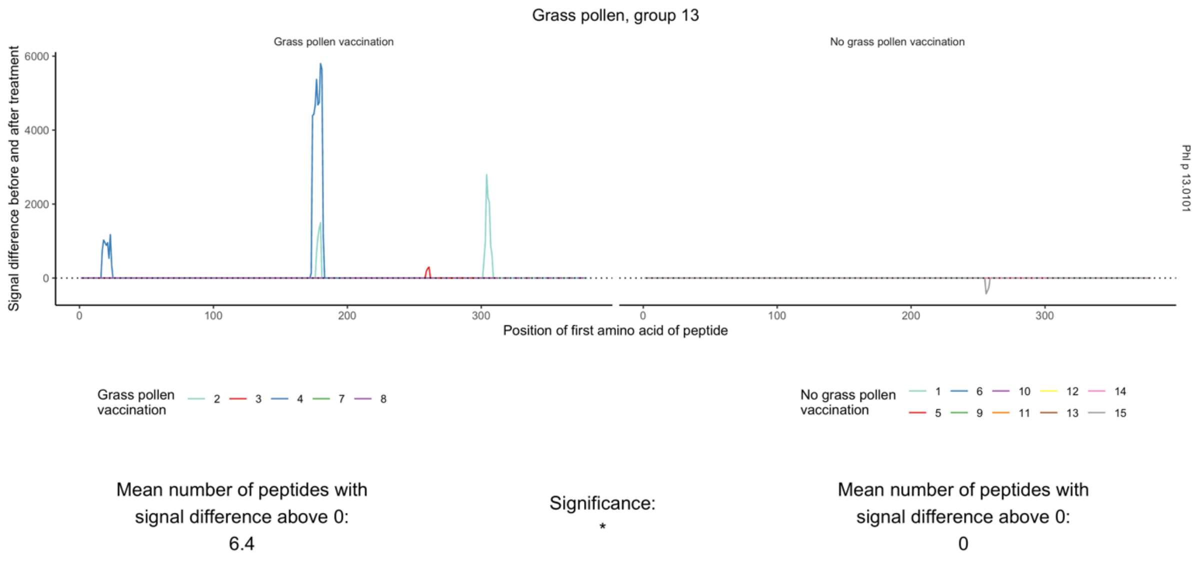

Supplementary Figure 6. Increase in IgE signal for each grass pollen allergen peptide after 3 years of AIT compared to at treatment initiation. For donors where no 3-years sample had been collected, the 1-year sample was used instead. Epitope A (yellow) and B (blue) have been marked for group 5 and 6 grass pollen allergens. Before comparison of samples, the background signal has been removed and signals for non-reactive peptides have been set to 0 in order to limit background noise. The mean number of peptides with an increased IgG signal over time is presented for donors which had or had not been subjected to AIT. Significant differences between these two groups were evaluated using Mann–Whitney U test. *: p value < 0.05, **: p value < 0.01, ns: no significant difference.
