# Supplementary material for: Linear Epitope Binding Patterns of Grass Pollen-Specific Antibodies in Allergy and in Response to Allergen-Specific Immunotherapy
Source: Front Allergy. 2022 Mar 31;3:859126. doi: 10.3389/falgy.2022.859126 (PMC9234942; doi:10.3389/falgy.2022.859126)
Supplement: Supplementary file 2 [file Data_Sheet_2.ZIP › Supplementary Figure 14.pdf]

Acidic ribosomal protein 1

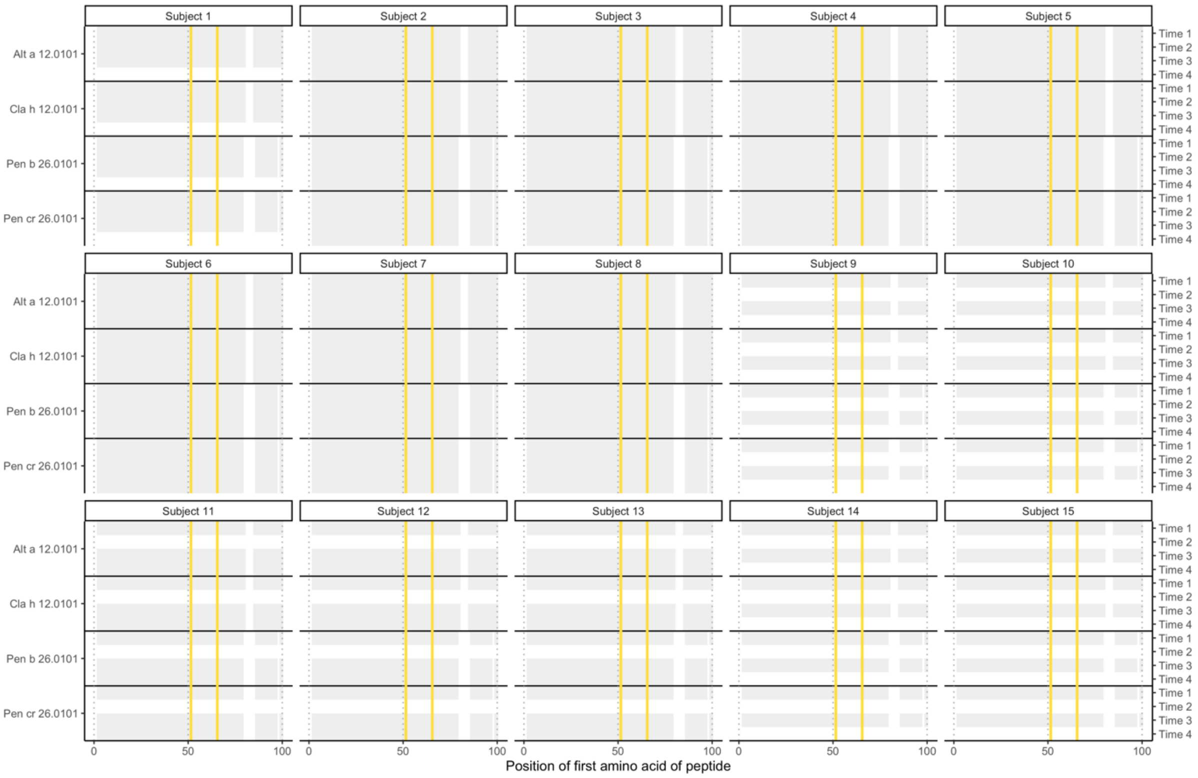

Acidic ribosomal protein 2

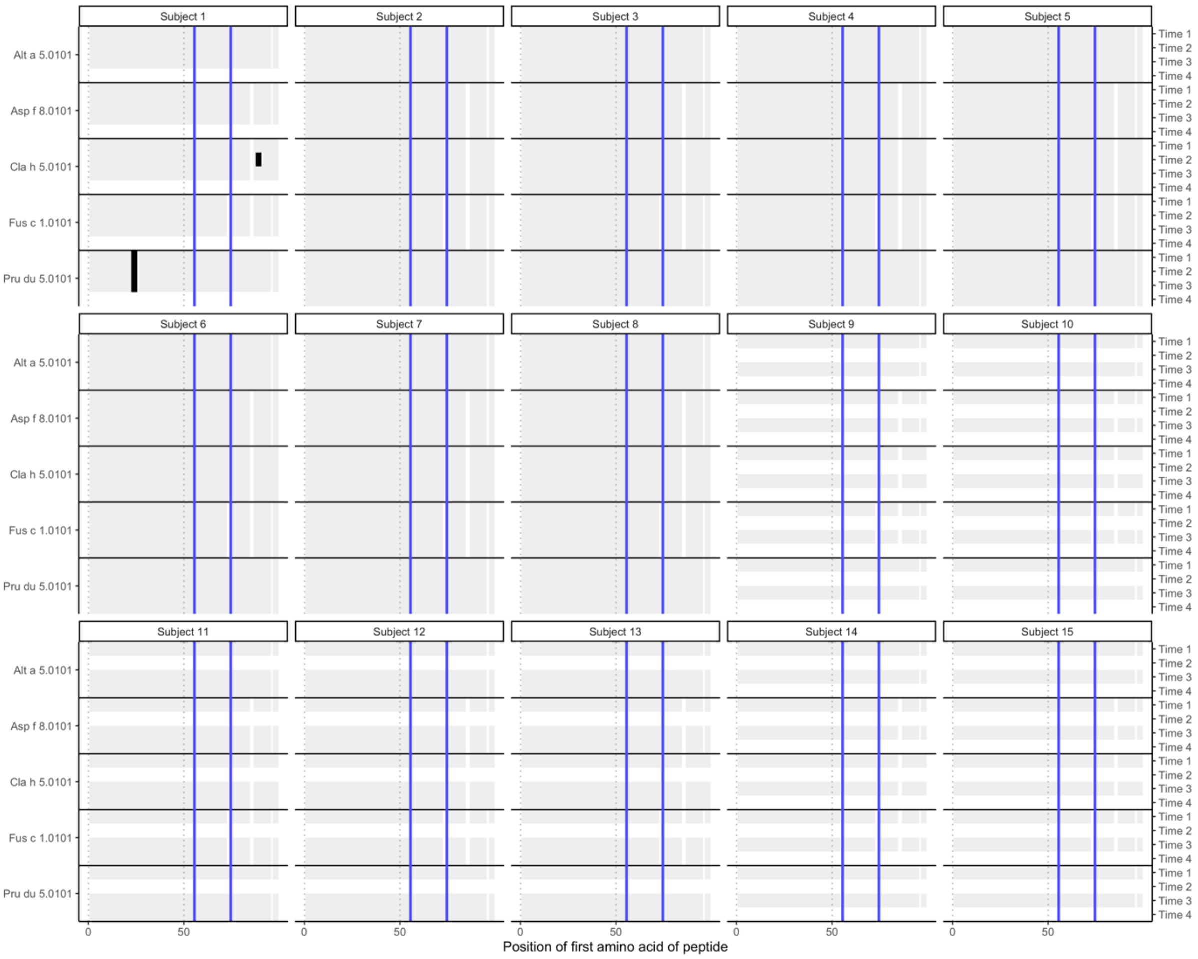

Supplementary Figure 14. Heatmap visualisation of IgG4 recognition of linear epitopes on acidic ribosomal protein 1 and 2 allergens, with reactive peptides in black and non-reactive peptides in grey. Epitope G (yellow) and H (blue) have been marked.
