# Supplementary material for: Linear Epitope Binding Patterns of Grass Pollen-Specific Antibodies in Allergy and in Response to Allergen-Specific Immunotherapy
Source: Front Allergy. 2022 Mar 31;3:859126. doi: 10.3389/falgy.2022.859126 (PMC9234942; doi:10.3389/falgy.2022.859126)
Supplement: Supplementary file 2 [file Data_Sheet_2.ZIP › Supplementary Figure 7.pdf]

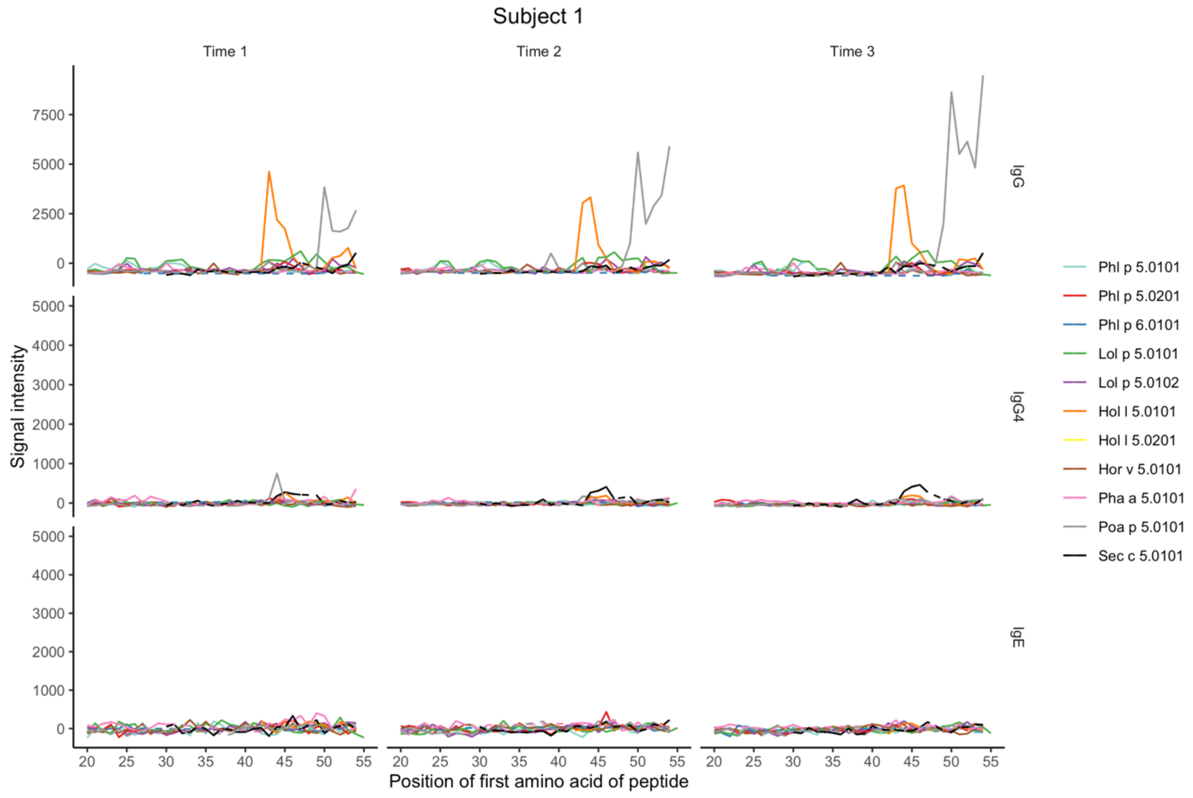

Average signal intensity for peptides starting at amino acid 21-53

	Time 0	Time 3	Significance
**IgG**	-244	-270	ns
**IgG4**	3	-14	ns
**IgE**	9	-22	ns

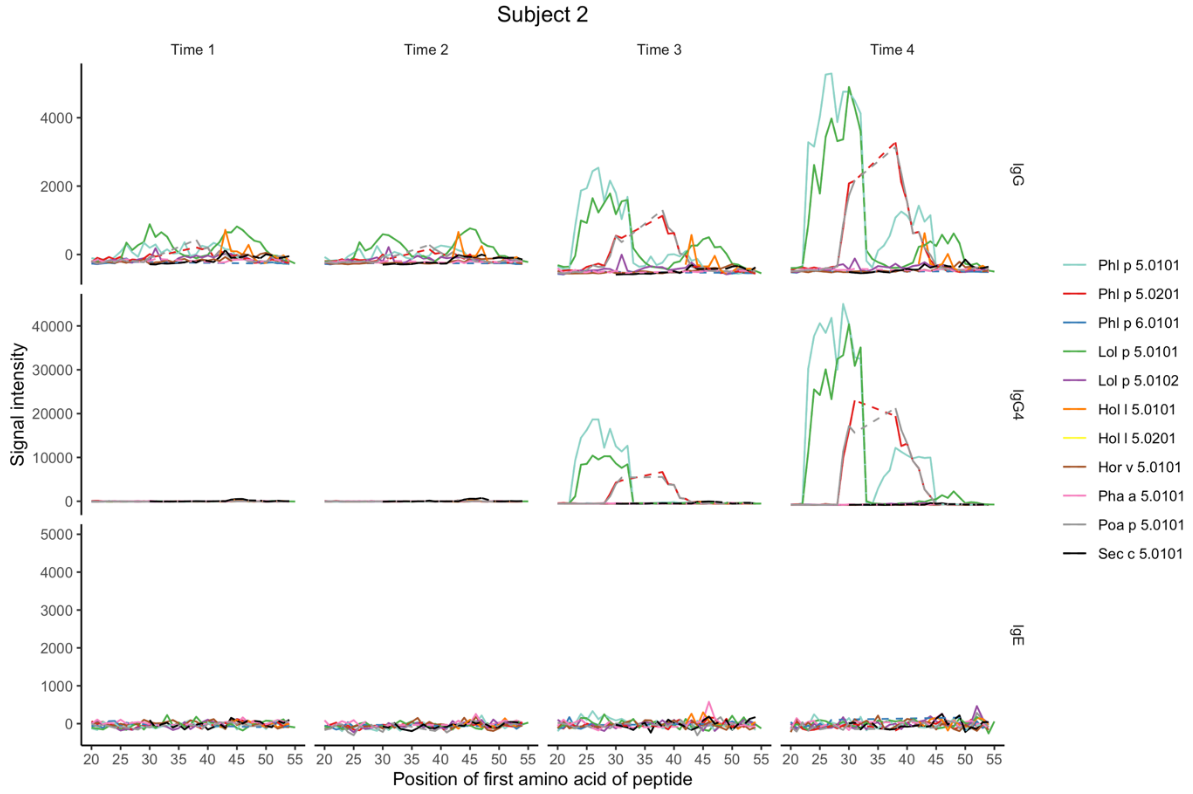

Average signal intensity for peptides starting at amino acid 21-53

	Time 0	Time 4	Significance
**IgG**	-59	152	ns
**IgG4**	-5	3495	**
**IgE**	-22	-20	ns

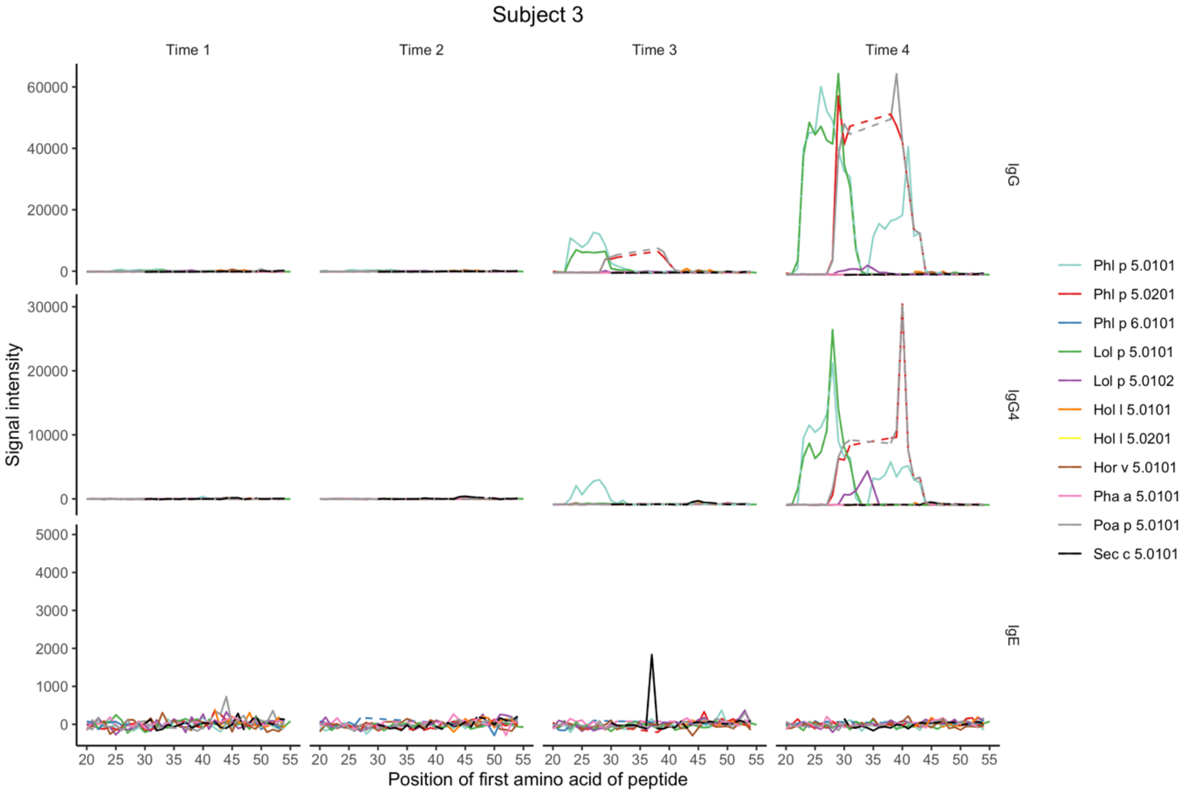

Average signal intensity for peptides
starting at amino acid 21-53

	Time 0	Time 4	Significance
**IgG**	-48	6039	**
**IgG4**	-6	1050	*
**IgE**	9	-3	ns

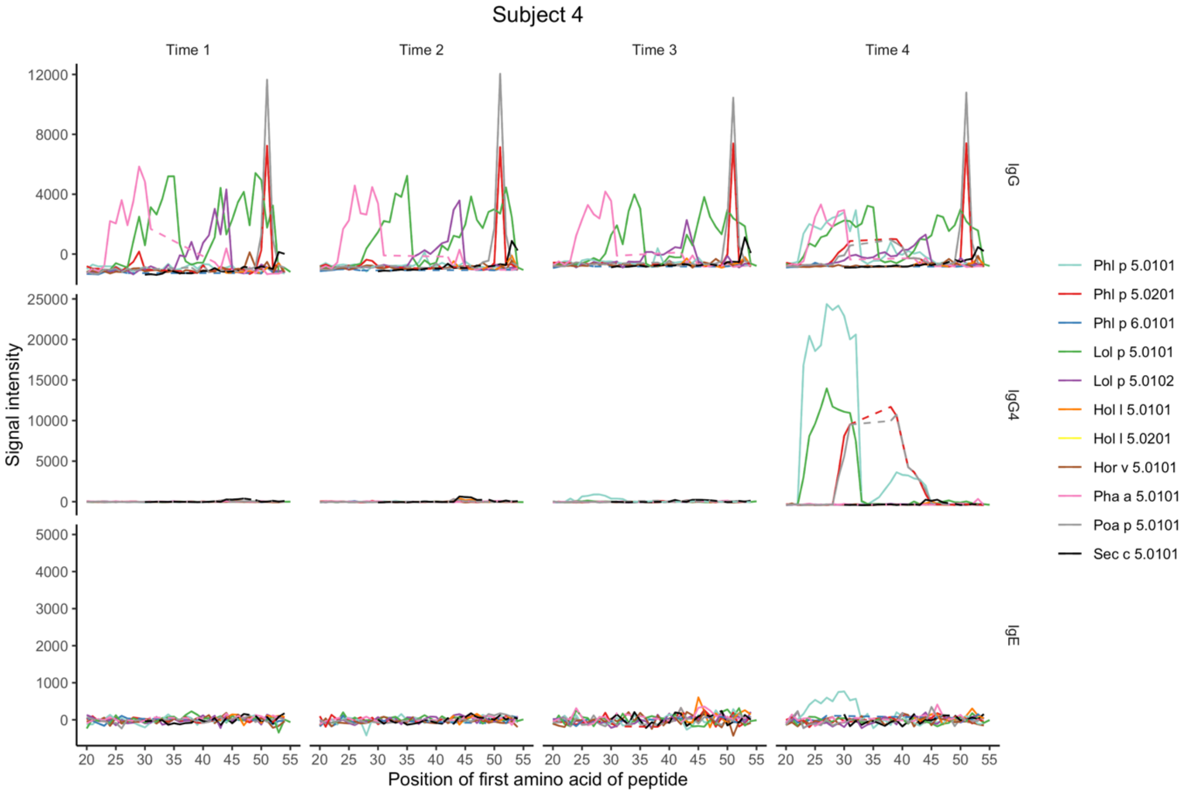

Average signal intensity for peptides starting at amino acid 21-53

	Time 0	Time 4	Significance
**IgG**	-395	-20	**
**IgG4**	-7	1600	**
**IgE**	-3	13	ns

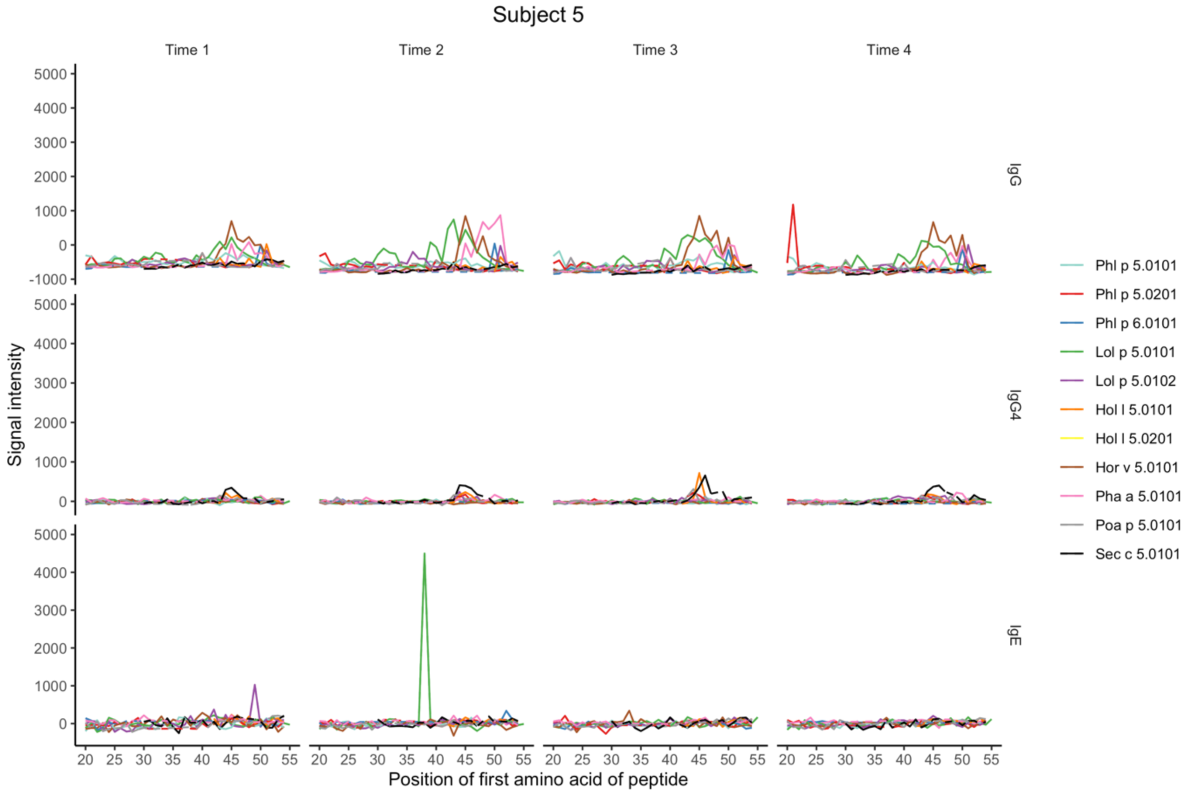

Average signal intensity for peptides starting at amino acid 21-53

	Time 0	Time 4	Significance
**IgG**	-501	-662	ns
**IgG4**	-1	-1	ns
**IgE**	10	-1	ns

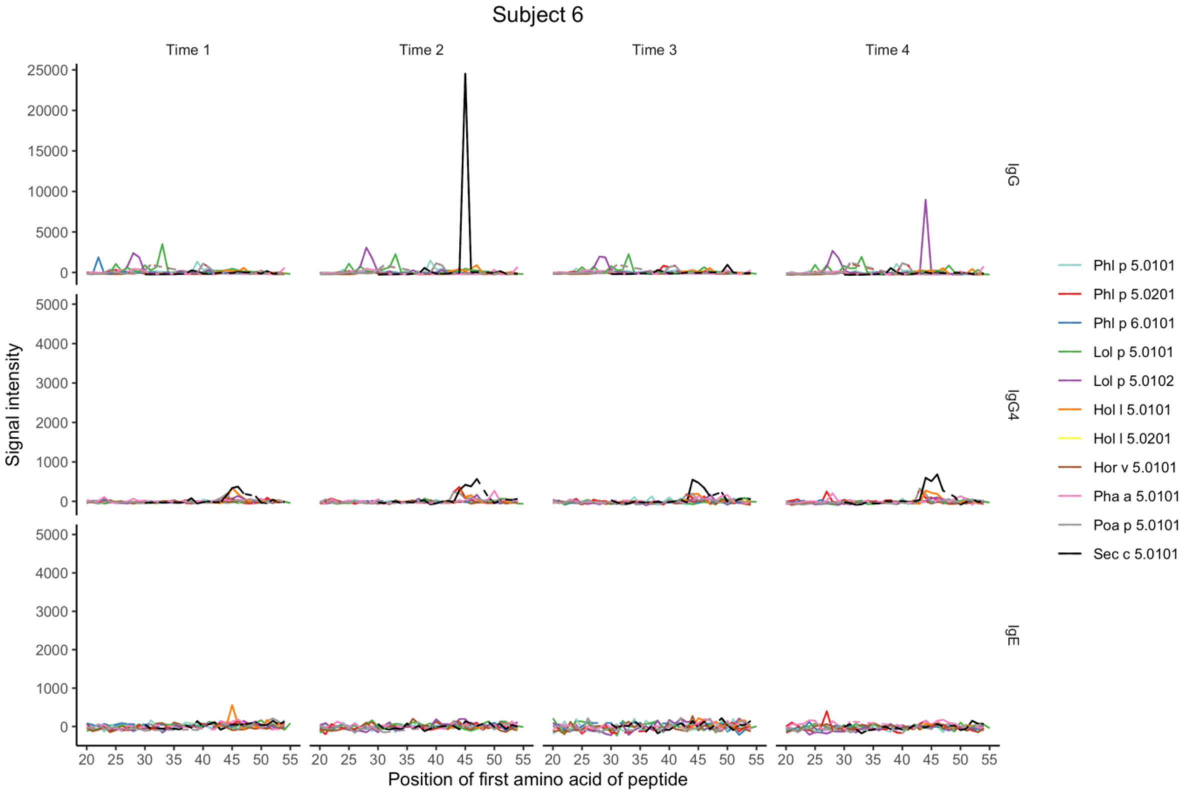

Average signal intensity for peptides starting at amino acid 21-53

	Time 0	Time 4	Significance
**IgG**	61	26	ns
**IgG4**	-3	-9	ns
**IgE**	8	-26	ns

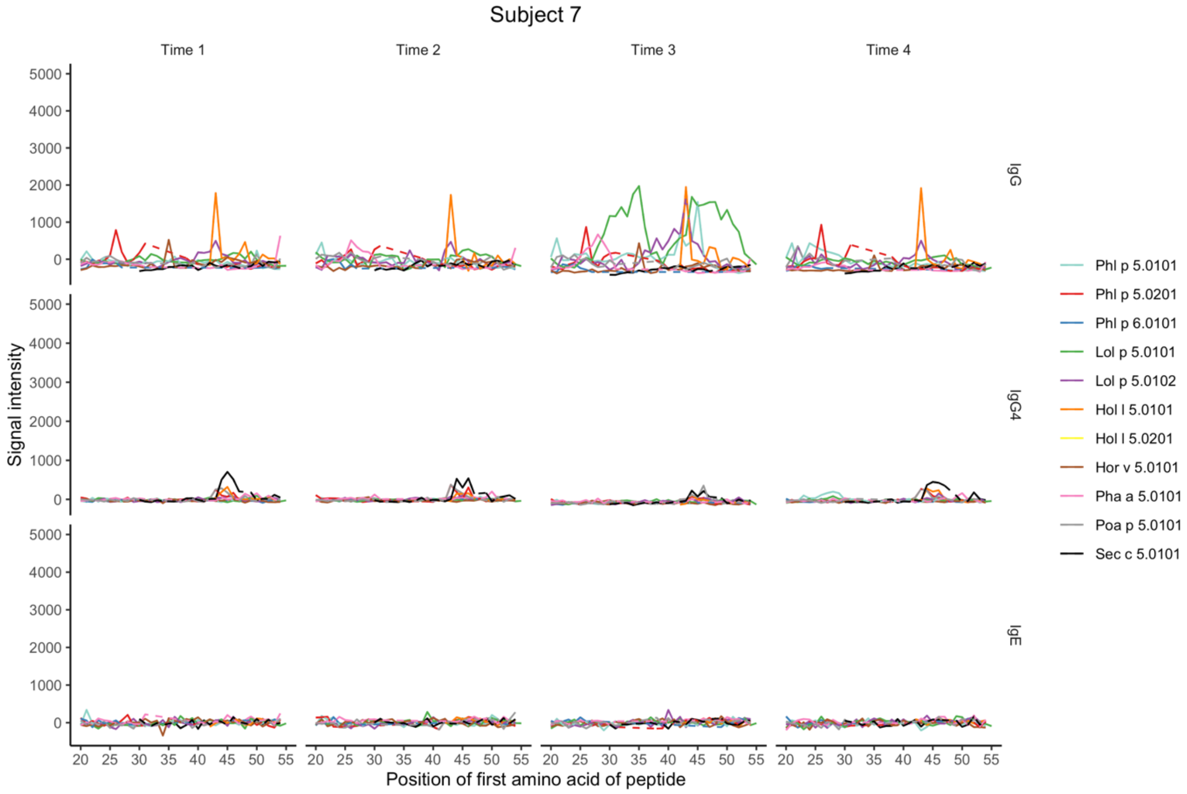

Average signal intensity for peptides starting at amino acid 21-53

	Time 0	Time 4	Significance
**IgG**	-82	-133	ns
**IgG4**	-1	-15	ns
**IgE**	-2	1	ns

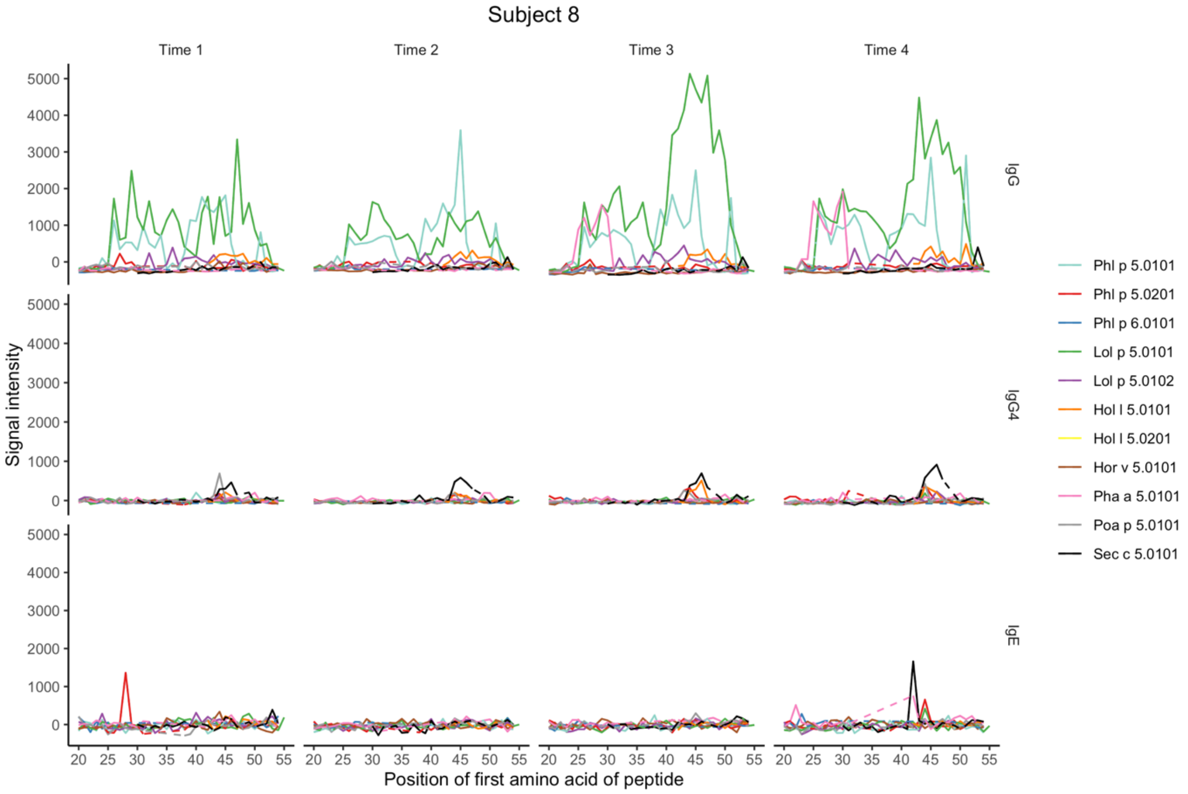

Average signal intensity for peptides starting at amino acid 21-53

	Time 0	Time 4	Significance
**IgG**	77	190	**
**IgG4**	-1	-11	ns
**IgE**	-8	-9	ns

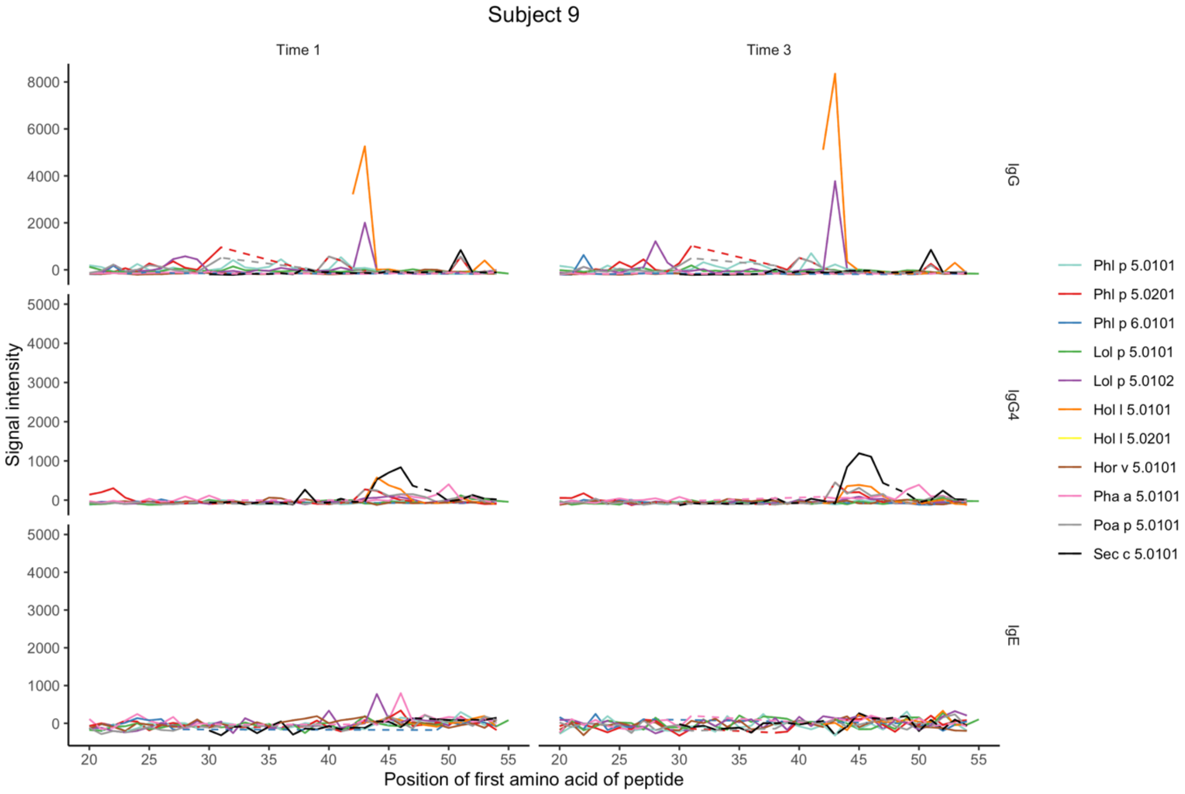

Average signal intensity for peptides
starting at amino acid 21-53

	Time 0	Time 3	Significance
**IgG**	6	20	ns
**IgG4**	-20	-18	ns
**IgE**	-25	-19	ns

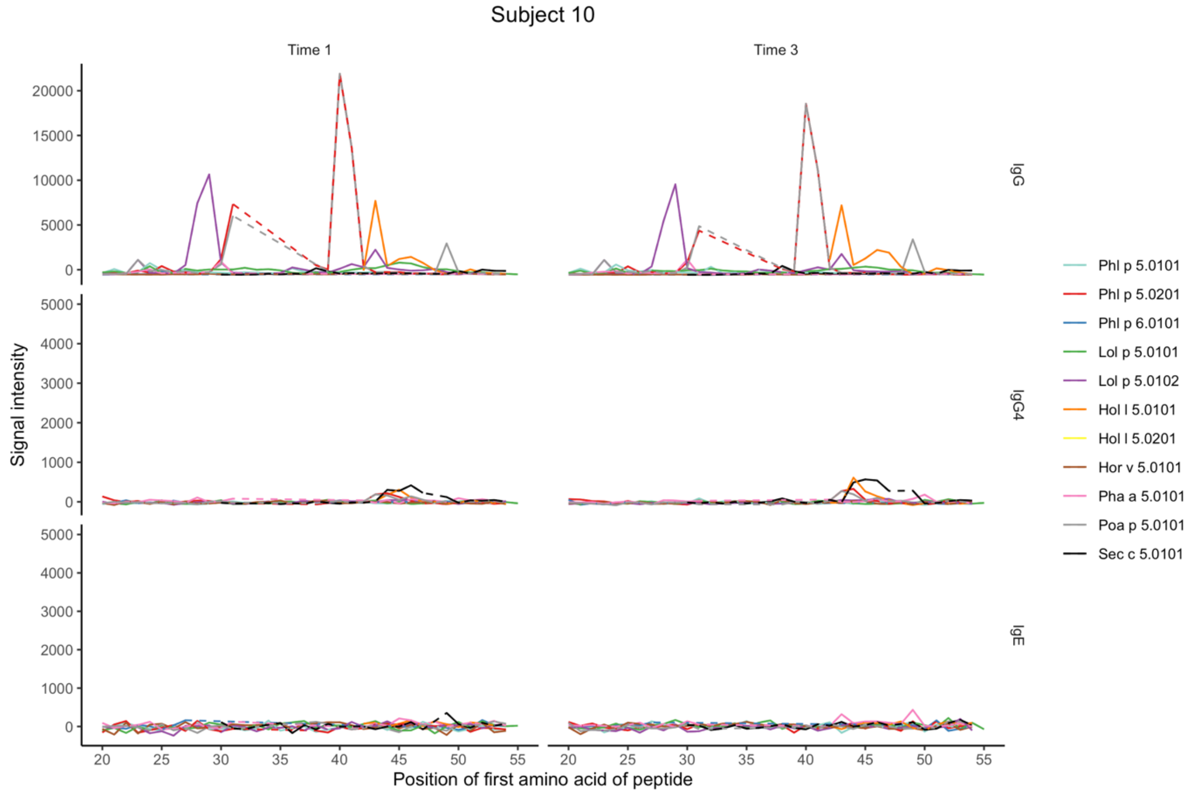

Average signal intensity for peptides
starting at amino acid 21-53

	Time 0	Time 3	Significance
**IgG**	257	142	ns
**IgG4**	-1	-2	ns
**IgE**	0	9	*

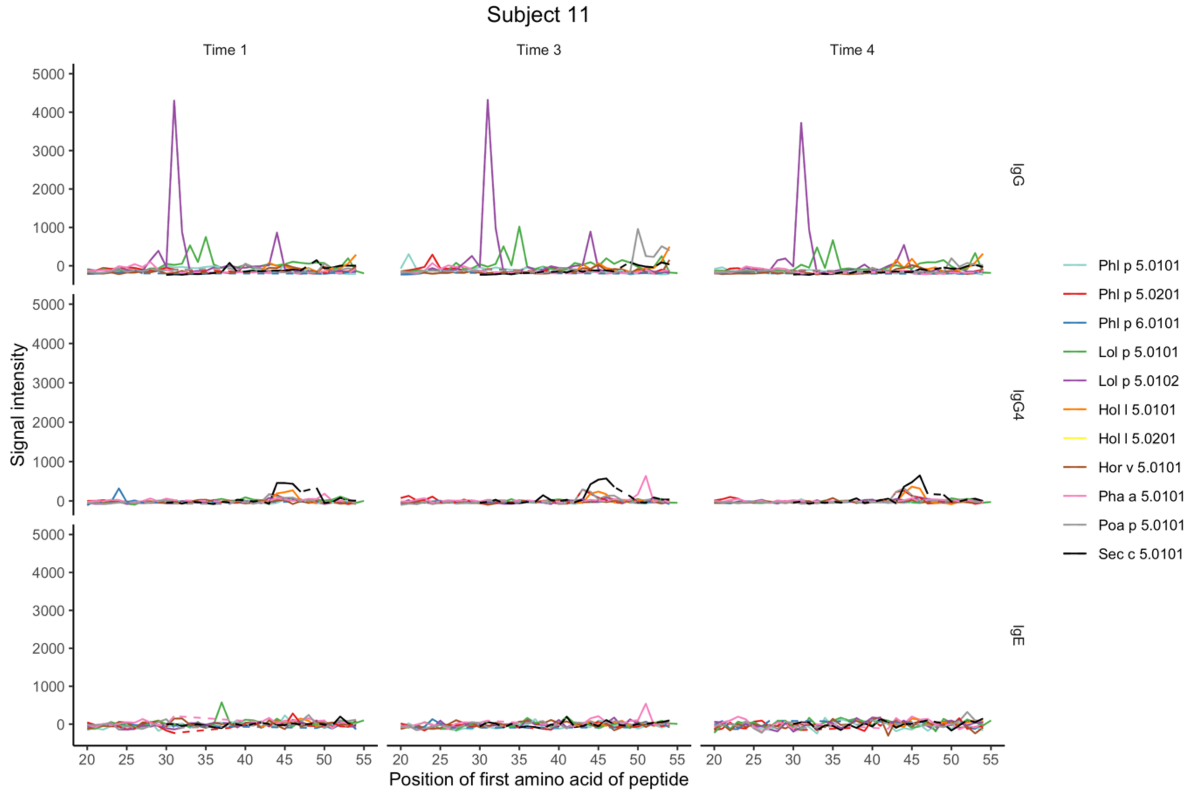

Average signal intensity for peptides starting at amino acid 21-53

	Time 0	Time 4	Significance
**IgG**	-66	-95	ns
**IgG4**	-2	-3	ns
**IgE**	-1	-3	ns

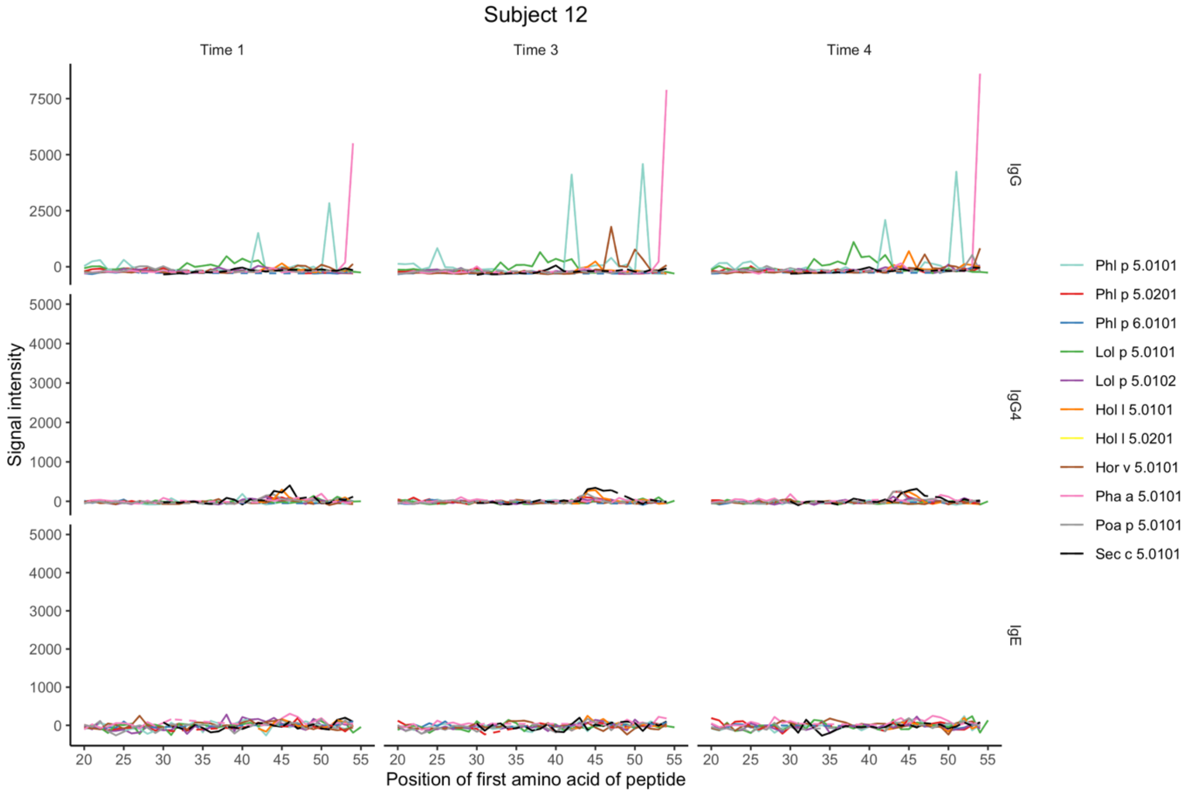

Average signal intensity for peptides
starting at amino acid 21-53

	Time 0	Time 4	Significance
**IgG**	-138	-104	**
**IgG4**	-3	-8	ns
**IgE**	-14	-16	ns

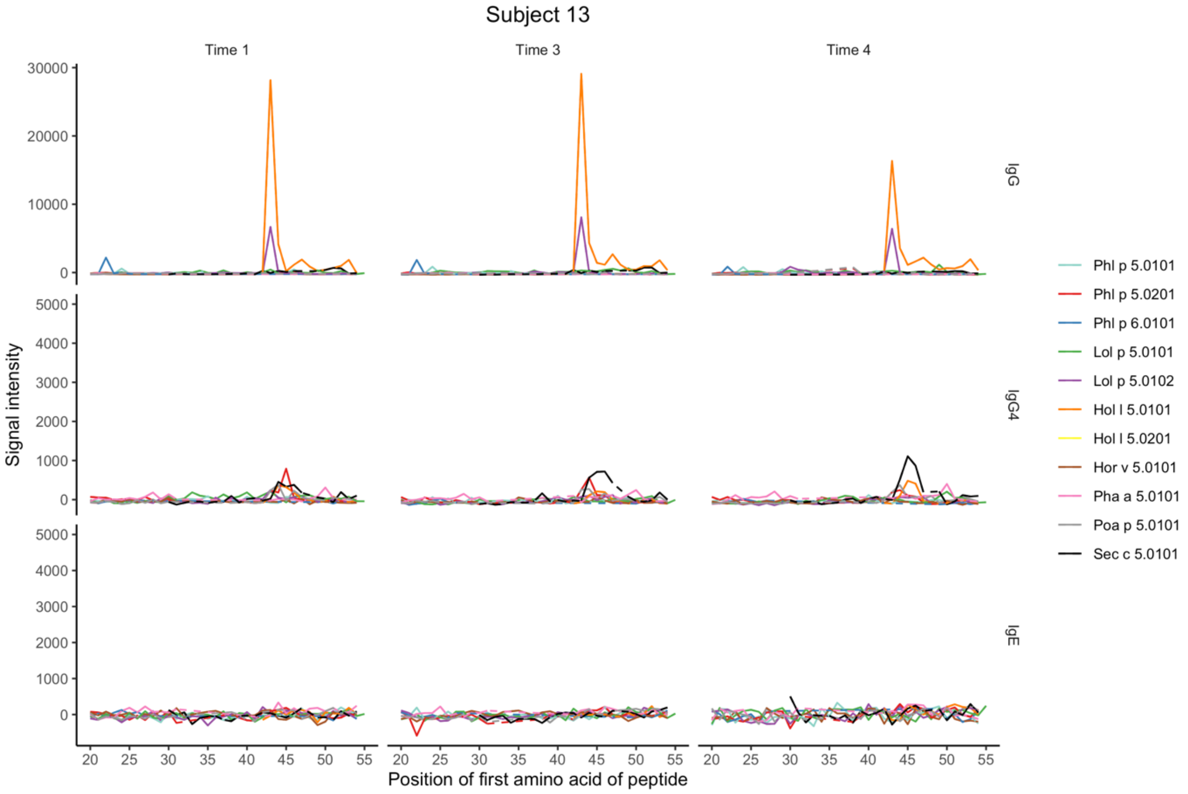

Average signal intensity for peptides
starting at amino acid 21-53

	Time 0	Time 4	Significance
**IgG**	53	28	ns
**IgG4**	1	-17	ns
**IgE**	-15	7	ns

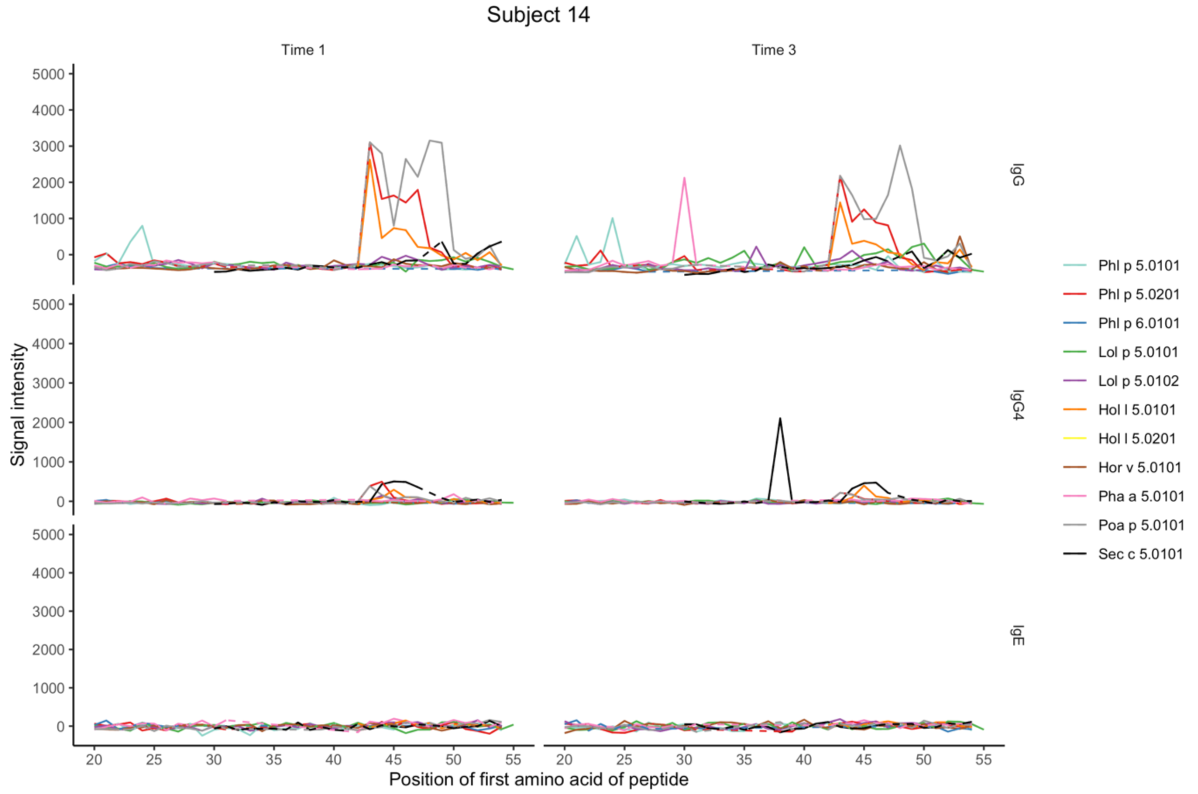

Average signal intensity for peptides starting at amino acid 21-53

	Time 0	Time 3	Significance
**IgG**	-160	-202	ns
**IgG4**	-7	3	ns
**IgE**	-10	-1	*

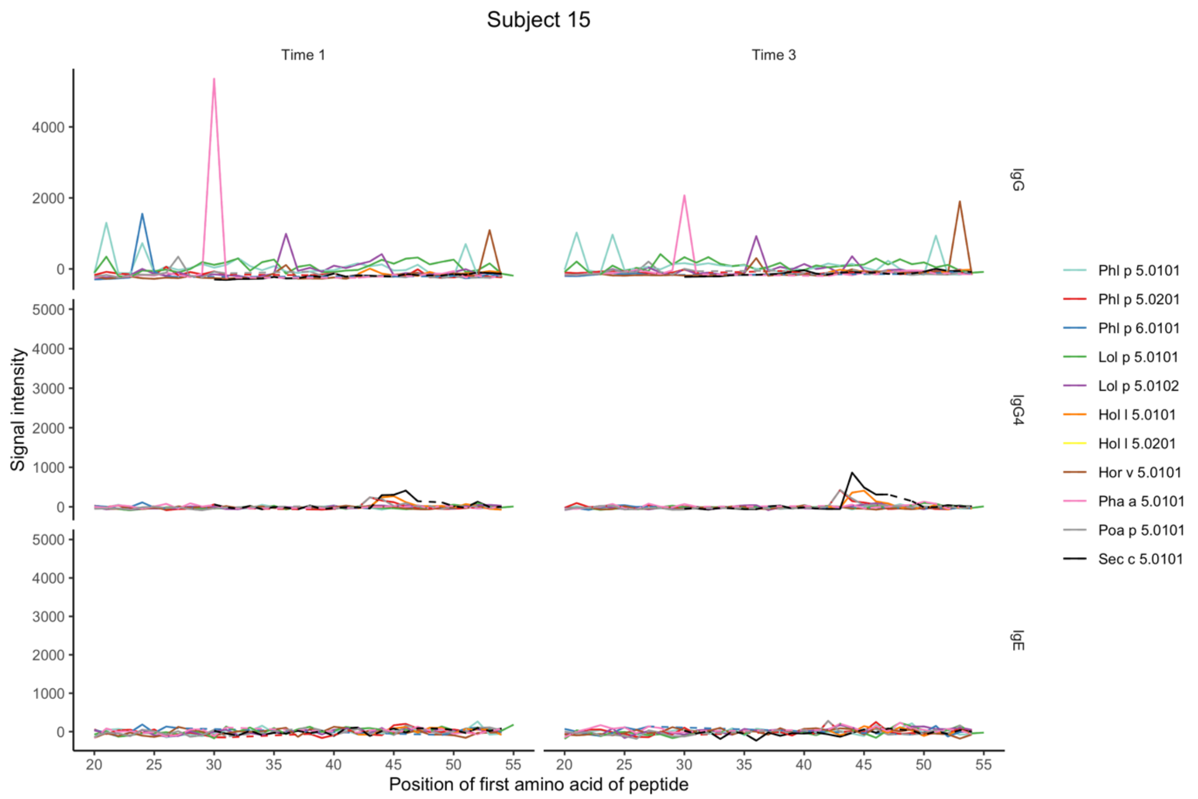

Average signal intensity for peptides
starting at amino acid 21-53

	Time 0	Time 3	Significance
**IgG**	-67	-28	**
**IgG4**	-6	0	ns
**IgE**	-3	0	ns

Supplementary Figure 7. IgG, IgG4, and IgE signal intensities for peptide 20-55 of group 5 and 6 grass pollen allergens. Samples had been collected at AIT treatment initiation (time 1), and 8 weeks (time 2), 1 year (time 3), and 3 years later (time 4). Donor 2, 3, 4, 7, and 8 were subjected to grass pollen AIT. Epitope A is represented by the peptides starting at position 21-53. The average signal intensities for peptides of epitope A are presented for the first sample (time 0) and the last sample (time 3 or 4) collected for each individual. Significant differences between the two samples were evaluated using one-sided Wilcoxon signed-rank test, to examine in which individuals the antibody response against epitope A increased during the investigated time period. *: p value < 0.05, **: p value < 0.01, ns: no significant difference.
