# Supplementary material for: Linear Epitope Binding Patterns of Grass Pollen-Specific Antibodies in Allergy and in Response to Allergen-Specific Immunotherapy
Source: Front Allergy. 2022 Mar 31;3:859126. doi: 10.3389/falgy.2022.859126 (PMC9234942; doi:10.3389/falgy.2022.859126)
Supplement: Supplementary file 2 [file Data_Sheet_2.ZIP › Supplementary Figure 8.pdf]

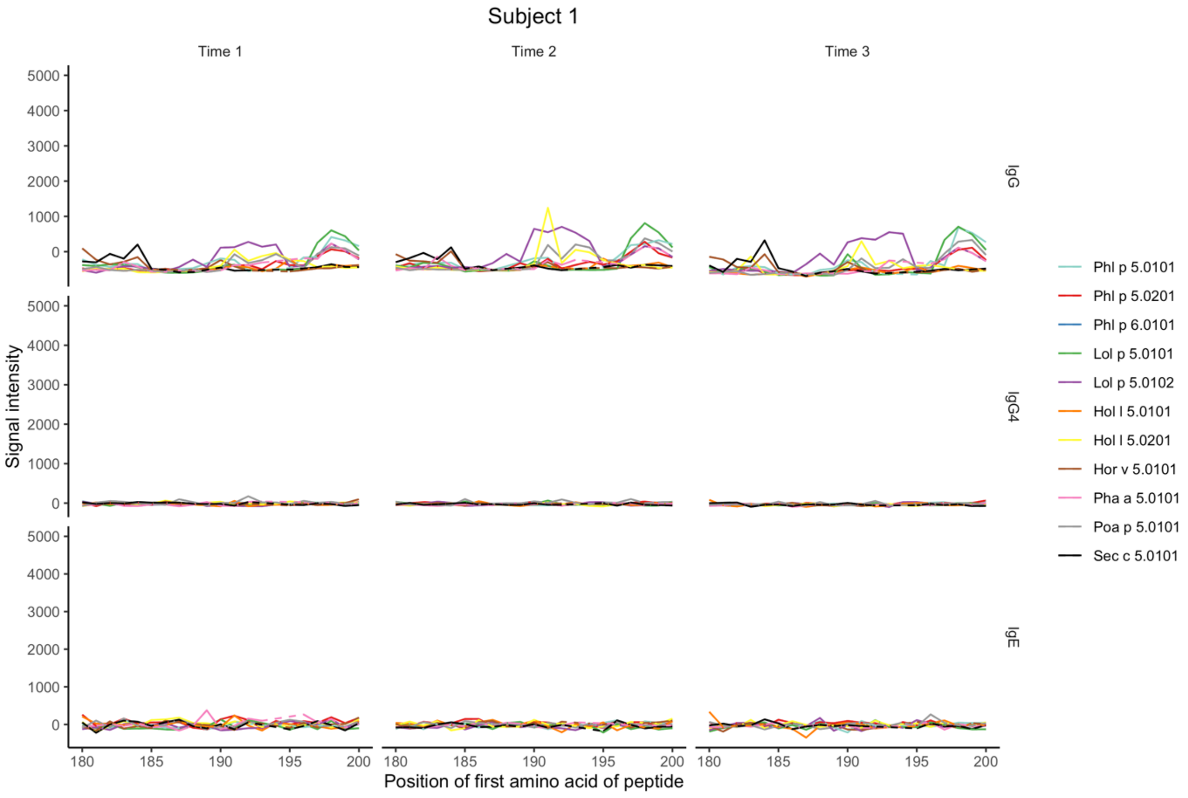

Average signal intensity for peptides starting at amino acid 182-196

	Time 0	Time 3	Significance
**IgG**	-404	-471	ns
**IgG4**	-16	-38	ns
**IgE**	-2	-21	ns

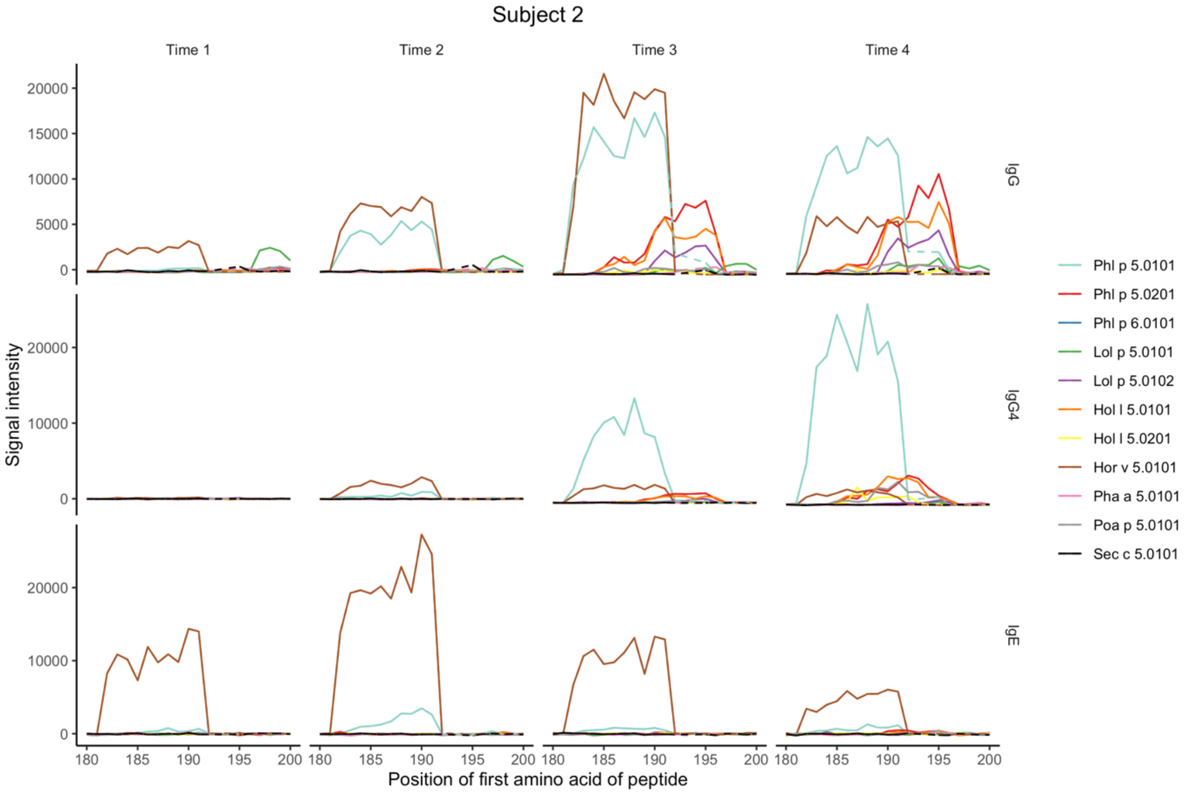

Average signal intensity for peptides starting at amino acid 182-196

	**Time 0**	**Time 4**	**Significance**
**IgG**	-19	1917	**
**IgG4**	-18	1108	**
**IgE**	705	369	ns

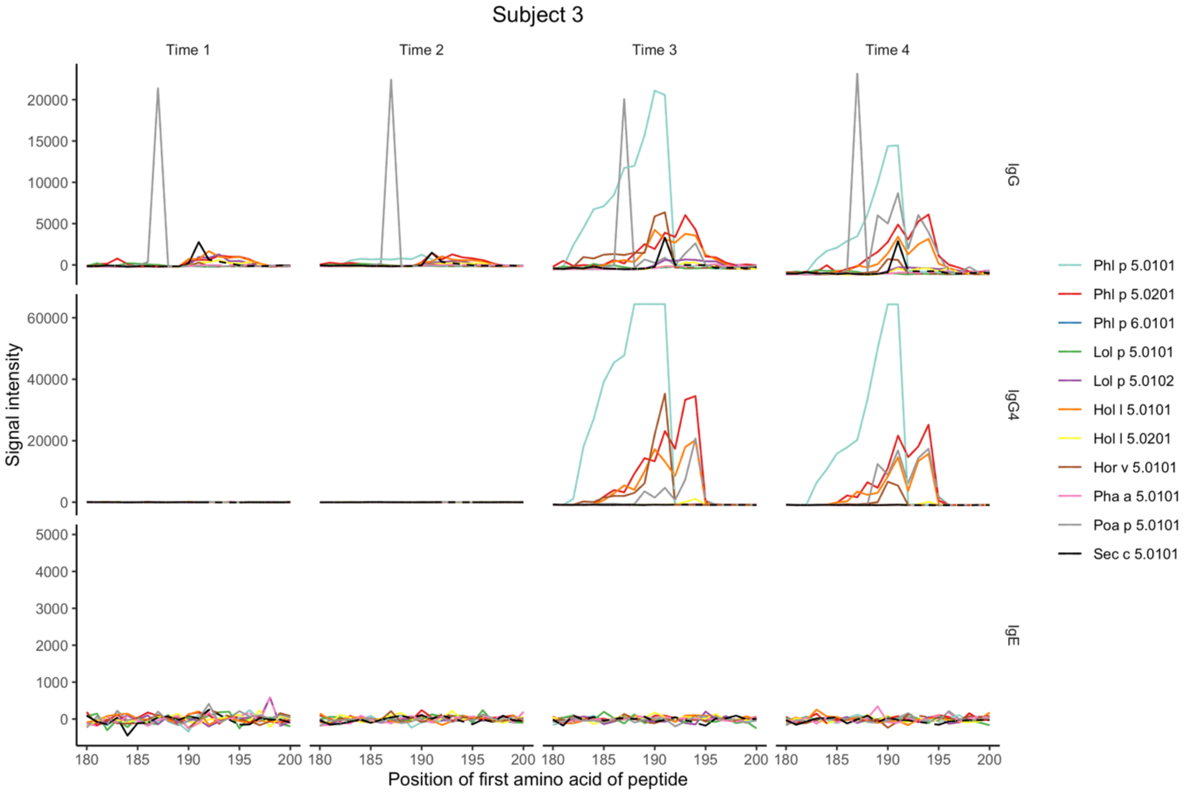

Average signal intensity for peptides starting at amino acid 182-196

	Time 0	Time 4	Significance
**IgG**	210	470	ns
**IgG4**	-19	3392	**
**IgE**	-3	-8	ns

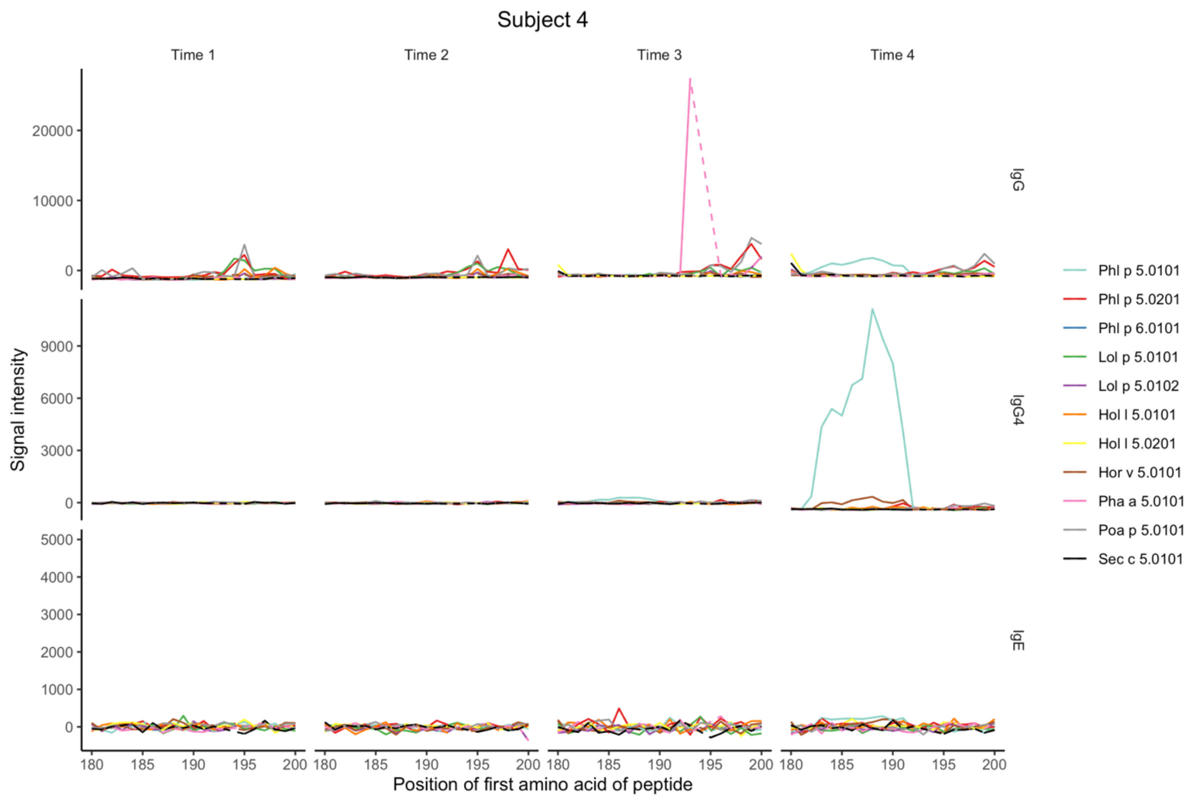

Average signal intensity for peptides starting at amino acid 182-196

	Time 0	Time 4	Significance
**IgG**	-915	-541	**
**IgG4**	-24	100	ns
**IgE**	5	10	ns

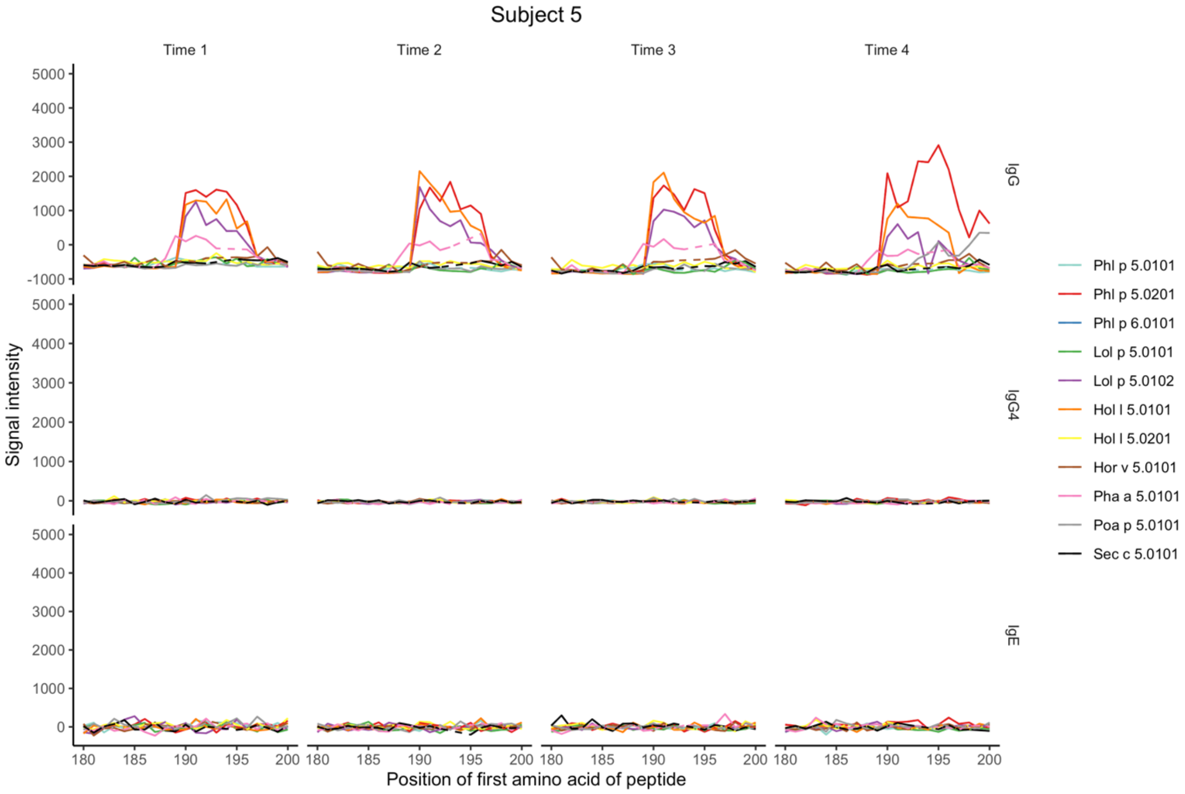

Average signal intensity for peptides
starting at amino acid 182-196

	Time 0	Time 4	Significance
**IgG**	-276	-424	ns
**IgG4**	-20	-26	ns
**IgE**	9	2	ns

Subject 6

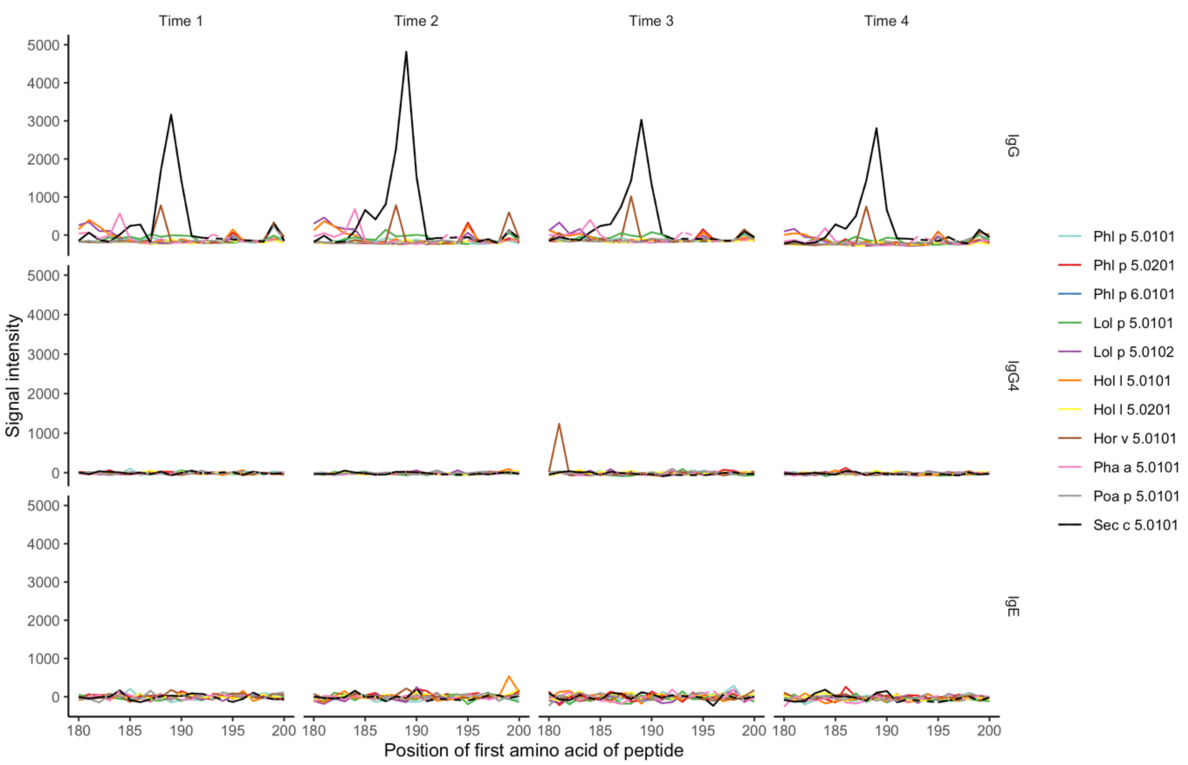

Average signal intensity for peptides starting at amino acid 182-196

	Time 0	Time 4	Significance
**IgG**	-82	-143	ns
**IgG4**	-12	-27	ns
**IgE**	-2	-35	ns

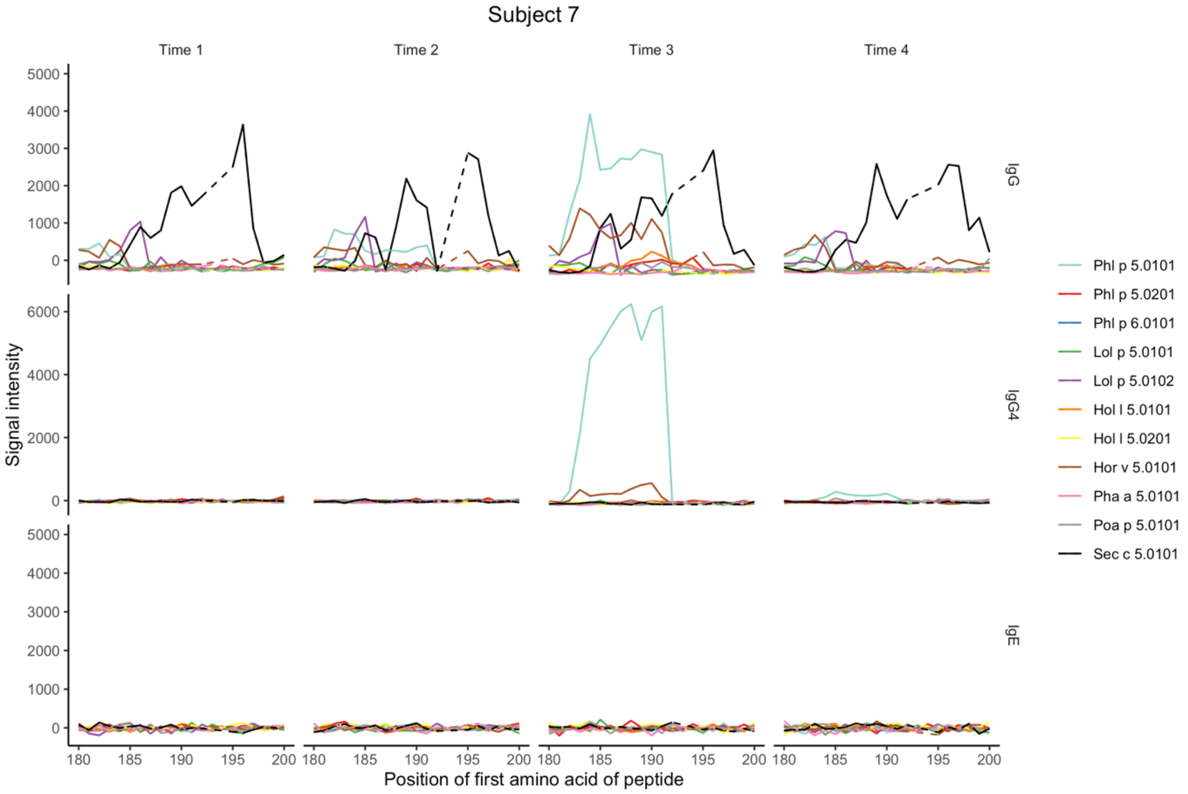

Average signal intensity for peptides starting at amino acid 182-196

	Time 0	Time 4	Significance
**IgG**	-45	-89	ns
**IgG4**	-24	-33	ns
**IgE**	-12	-6	ns

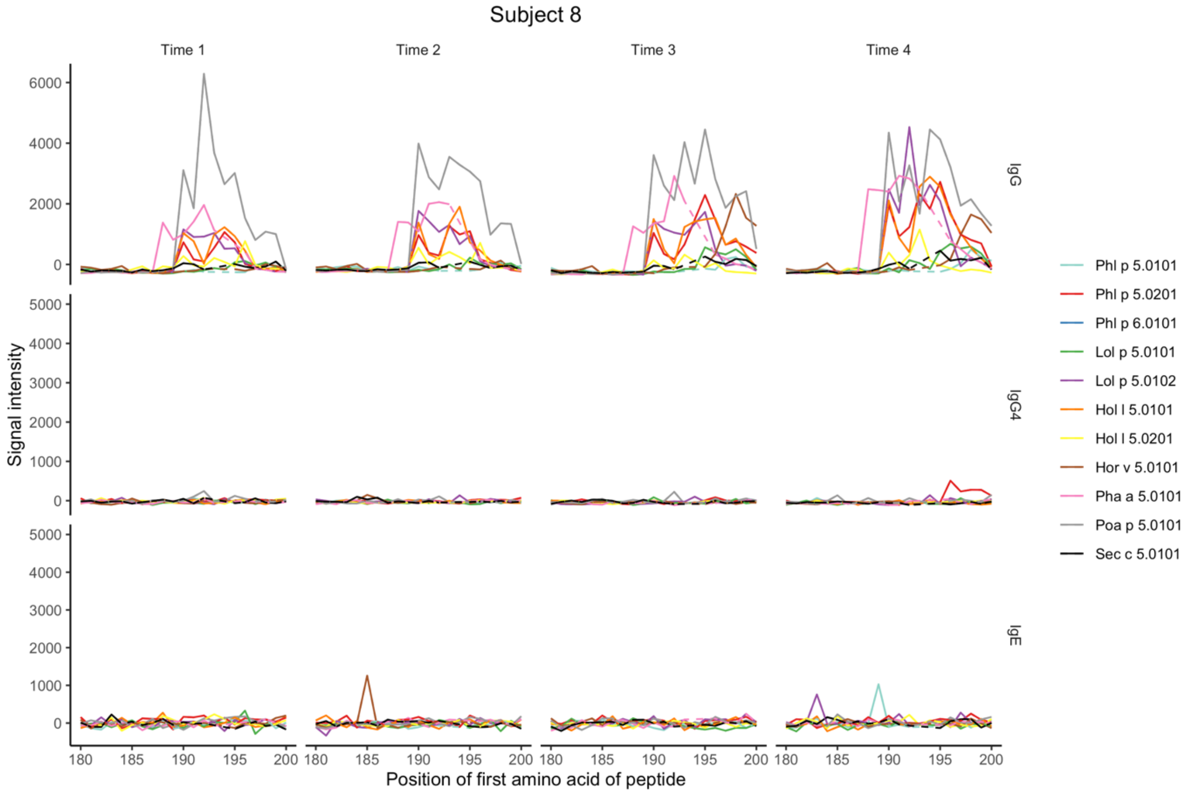

Average signal intensity for peptides starting at amino acid 182-196

	Time 0	Time 4	Significance
**IgG**	214	529	**
**IgG4**	-19	-41	ns
**IgE**	6	12	ns

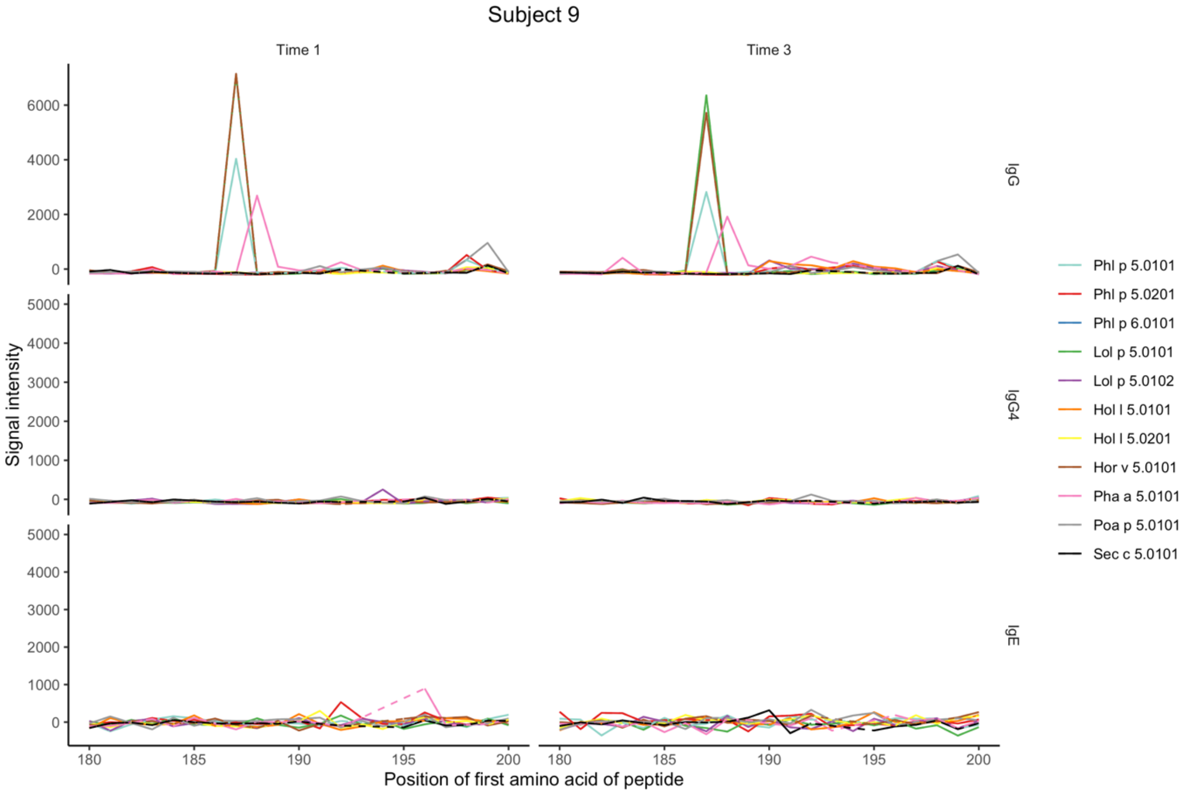

Average signal intensity for peptides starting at amino acid 182-196

	Time 0	Time 3	Significance
**IgG**	36	34	ns
**IgG4**	-57	-67	ns
**IgE**	-5	-6	ns

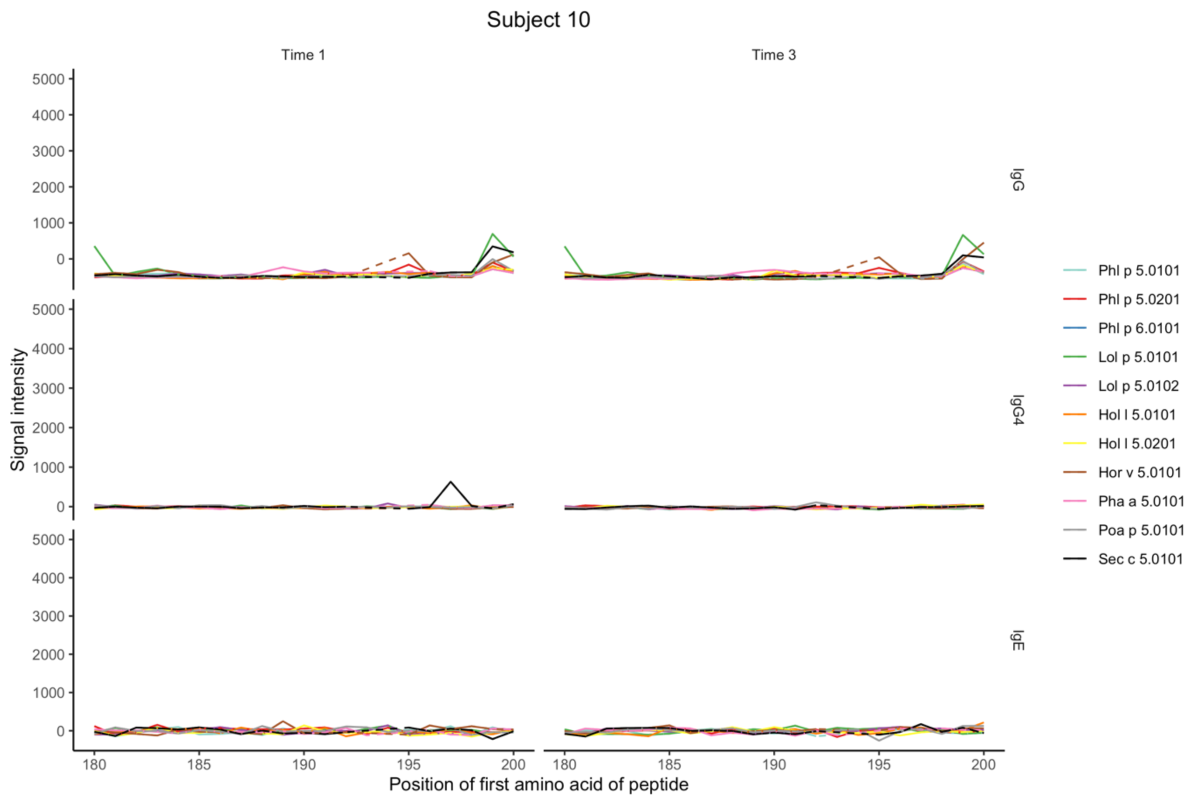

Average signal intensity for peptides starting at amino acid 182-196

	Time 0	Time 3	Significance
**IgG**	-461	-486	ns
**IgG4**	-19	-29	ns
**IgE**	-6	-8	ns

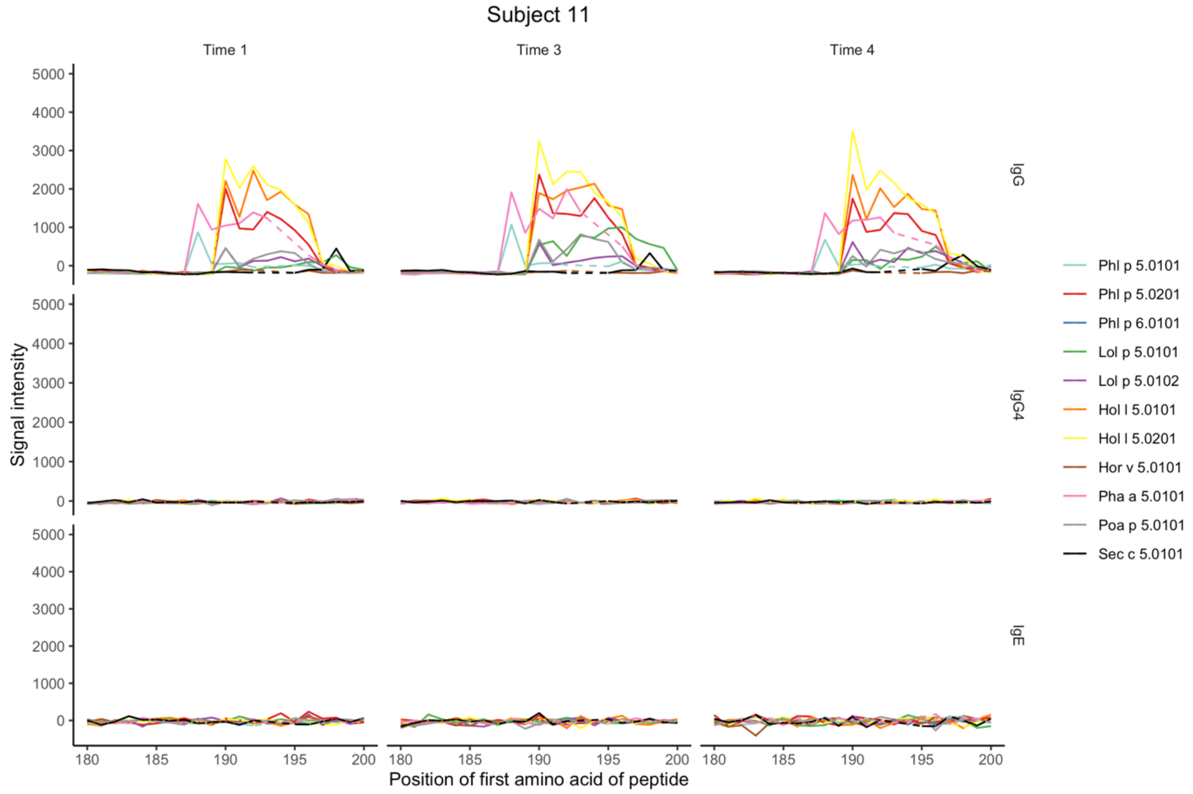

Average signal intensity for peptides starting at amino acid 182-196

	Time 0	Time 4	Significance
**IgG**	249	259	ns
**IgG4**	-30	-29	ns
**IgE**	-18	-20	ns

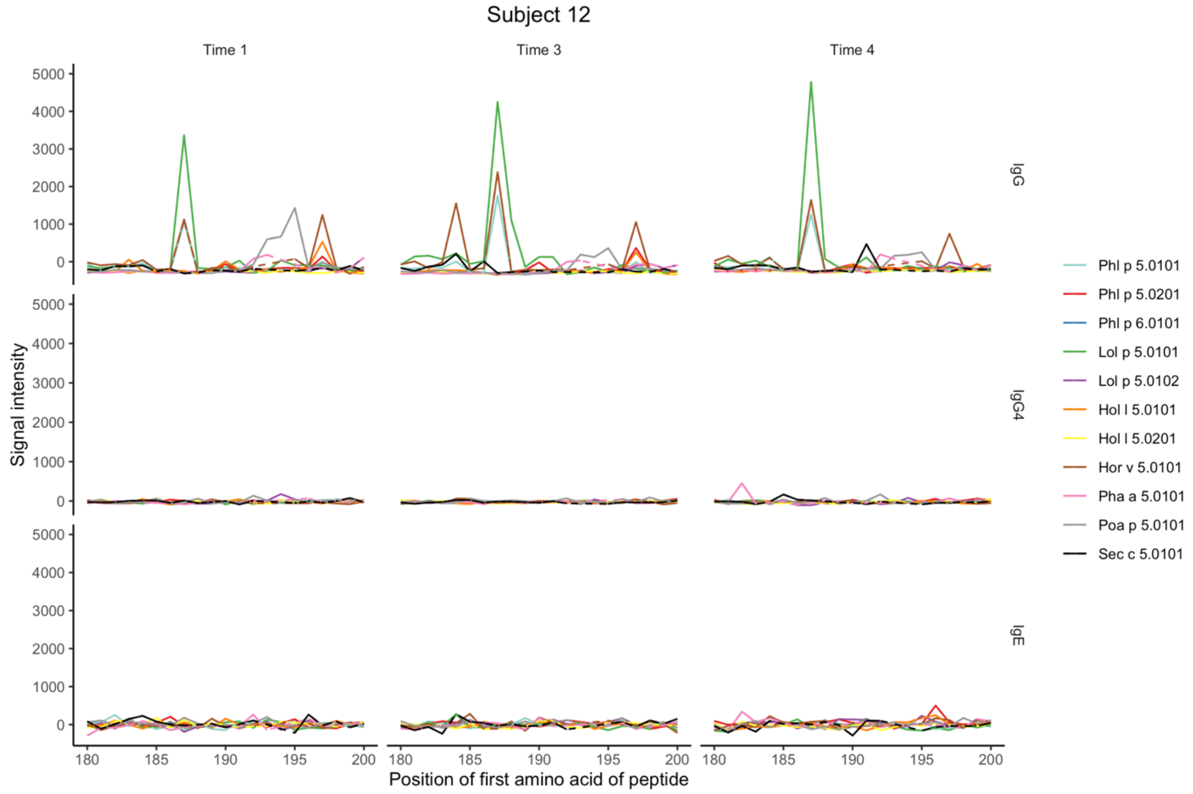

Average signal intensity for peptides starting at amino acid 182-196

	Time 0	Time 4	Significance
**IgG**	-137	-123	ns
**IgG4**	-20	-25	ns
**IgE**	5	5	ns

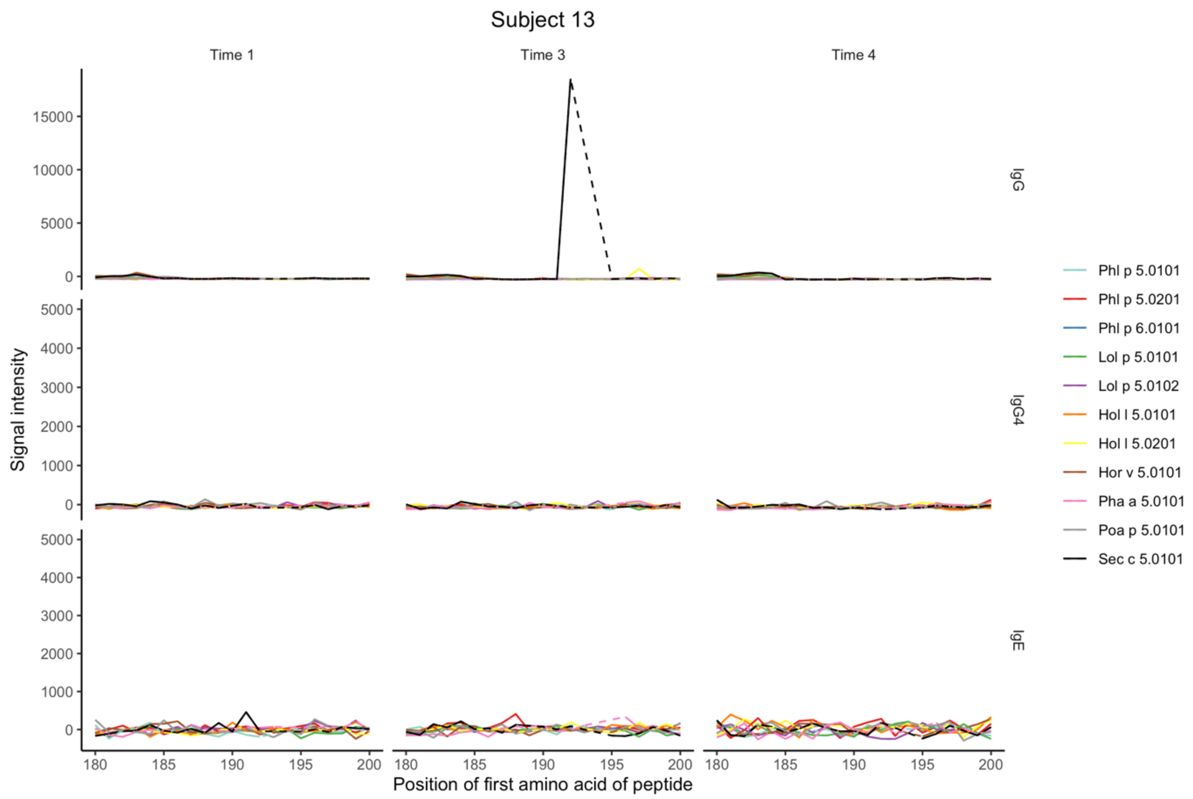

Average signal intensity for peptides starting at amino acid 182-196

	Time 0	Time 4	Significance
**IgG**	-200	-229	ns
**IgG4**	-42	-60	ns
**IgE**	-10	10	ns

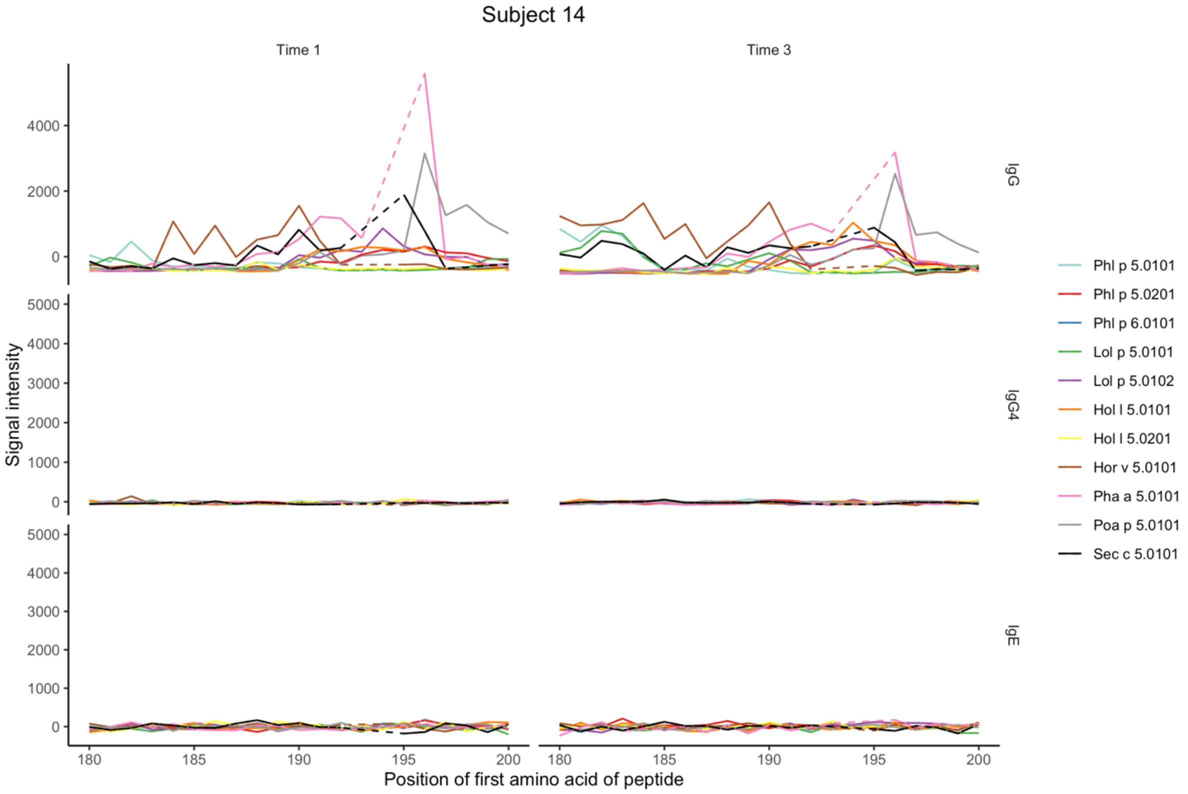

Average signal intensity for peptides starting at amino acid 182-196

	Time 0	Time 3	Significance
**IgG**	-34	-19	ns
**IgG4**	-32	-23	ns
**IgE**	-3	-5	ns

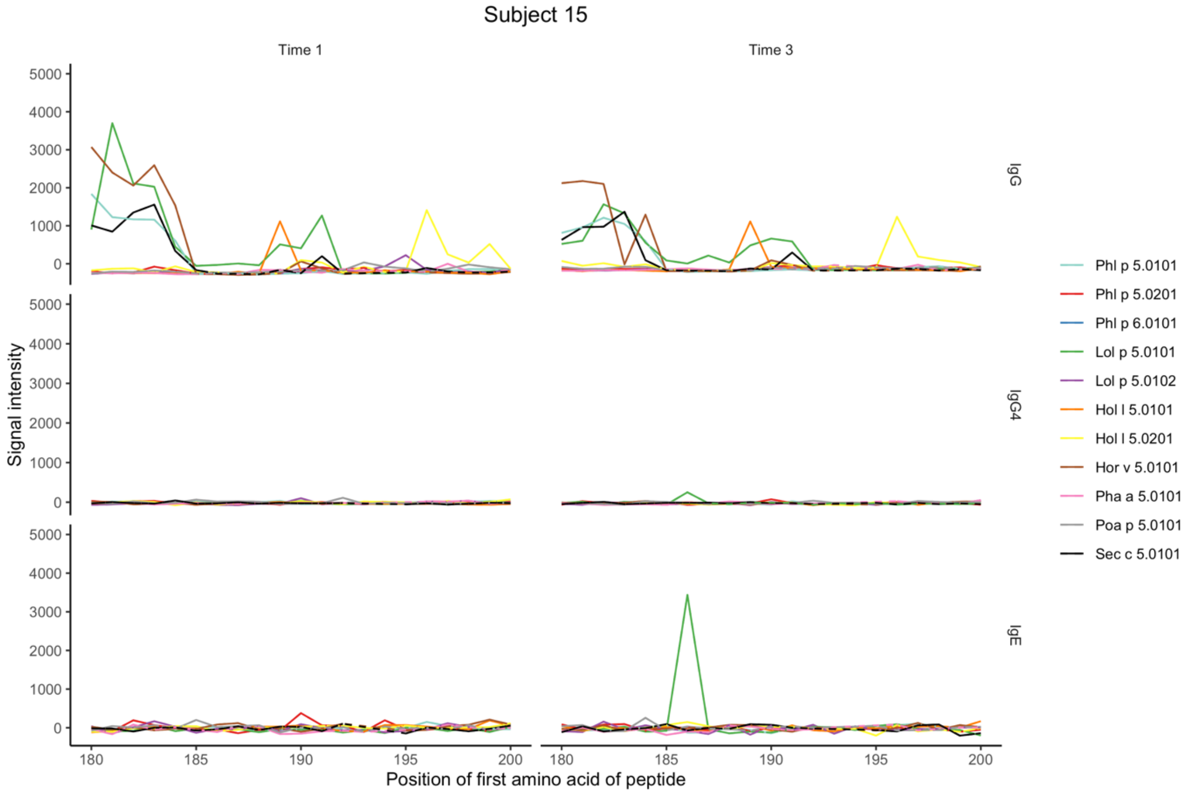

Average signal intensity for peptides
starting at amino acid 182-196

	Time 0	Time 3	Significance
**IgG**	-24	-6	*
**IgG4**	-20	-26	ns
**IgE**	-12	7	ns

Supplementary Figure 8. IgG, IgG4, and IgE signal intensities for peptide 180-200 of group 5 and 6 grass pollen allergens. Samples had been collected at AIT treatment initiation (time 1), and 8 weeks (time 2), 1 year (time 3), and 3 years later (time 4). Donor 2, 3, 4, 7, and 8 were subjected to grass pollen AIT. Epitope B is represented by the peptides starting at position 182-196. The average signal intensities for peptides of epitope A are presented for the first sample (time 0) and the last sample (time 3 or 4) collected for each individual. Significant differences between the two samples were evaluated using one-sided Wilcoxon signed-rank test, to examine in which individuals the antibody response against epitope B increased during the investigated time period. *: p value < 0.05, **: p value < 0.01, ns: no significant difference.
