# Supplementary material for: Linear Epitope Binding Patterns of Grass Pollen-Specific Antibodies in Allergy and in Response to Allergen-Specific Immunotherapy
Source: Front Allergy. 2022 Mar 31;3:859126. doi: 10.3389/falgy.2022.859126 (PMC9234942; doi:10.3389/falgy.2022.859126)
Supplement: Supplementary file 2 [file Data_Sheet_2.ZIP › Supplementary Figure 9.pdf]

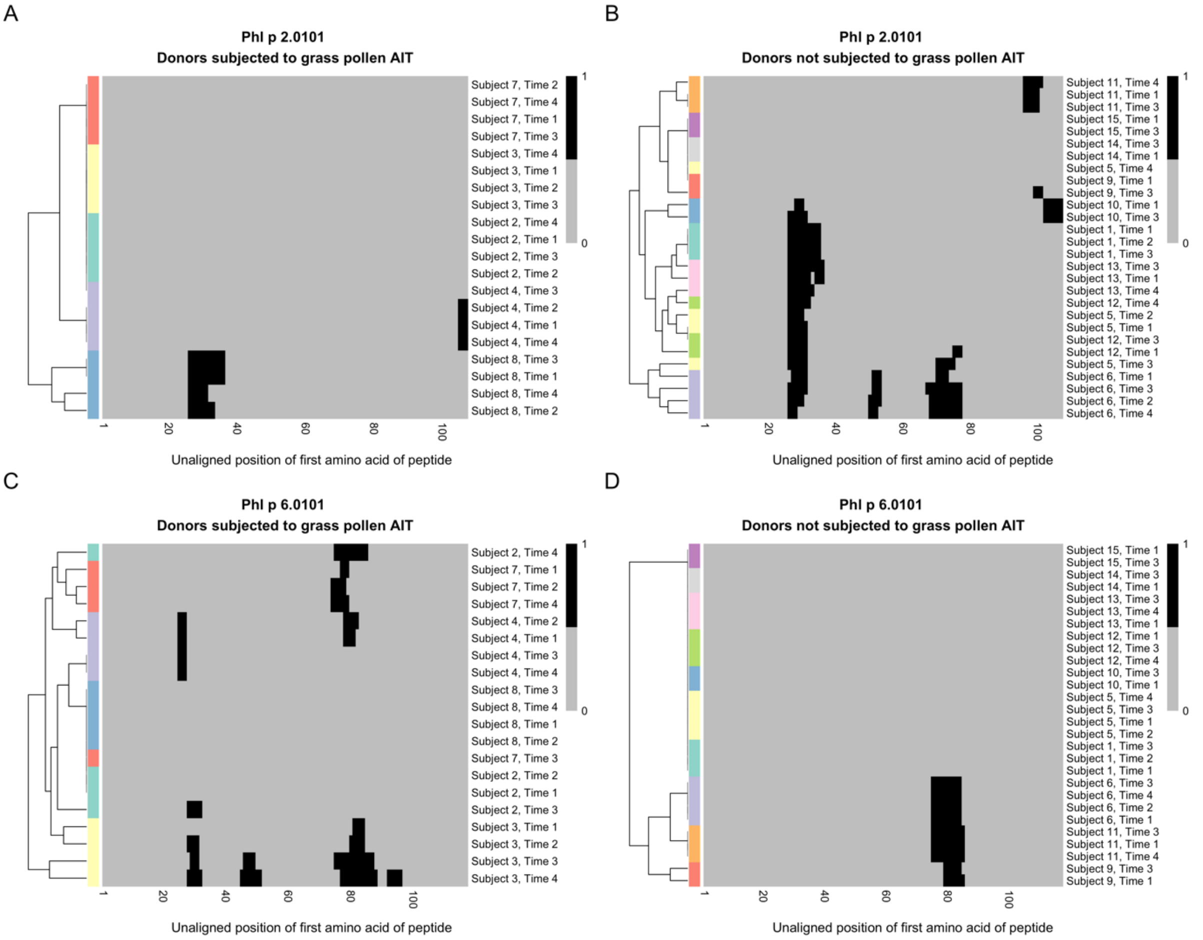

Supplementary Figure 9. Hierarchical clustering of IgG recognition of Phl p 2.0101 (**A-B**) and Phl p 6.0101 (**C-D**) peptides in serum samples. The samples had been collected at AIT initiation (time 1), and 8 weeks (time 2), 1 year (time 3), and 3 years later (time 4). Donor 2 (cyan), donor 3 (yellow), donor 4 (light purple), donor 7 (red), and donor 8 (blue) had been subjected to grass pollen AIT (**A, C**), while donor 1 (cyan), donor 5 (yellow), and donor 6 (light purple) had been subjected to AIT containing non-grass allergens. Donor 9 (red), donor 10 (blue), donor 11 (orange), donor 12 (green), donor 13 (pink), donor 14 (grey), and donor 15 (dark purple) had not been subjected to AIT at all (**B, D**). Clustering was done using euclidean distance and the R package 'pheatmap'.
