# Supplementary material for: Linear Epitope Binding Patterns of Grass Pollen-Specific Antibodies in Allergy and in Response to Allergen-Specific Immunotherapy
Source: Front Allergy. 2022 Mar 31;3:859126. doi: 10.3389/falgy.2022.859126 (PMC9234942; doi:10.3389/falgy.2022.859126)
Supplement: Supplementary file 2 [file Data_Sheet_2.ZIP › Supplementary Figure 10.pdf]

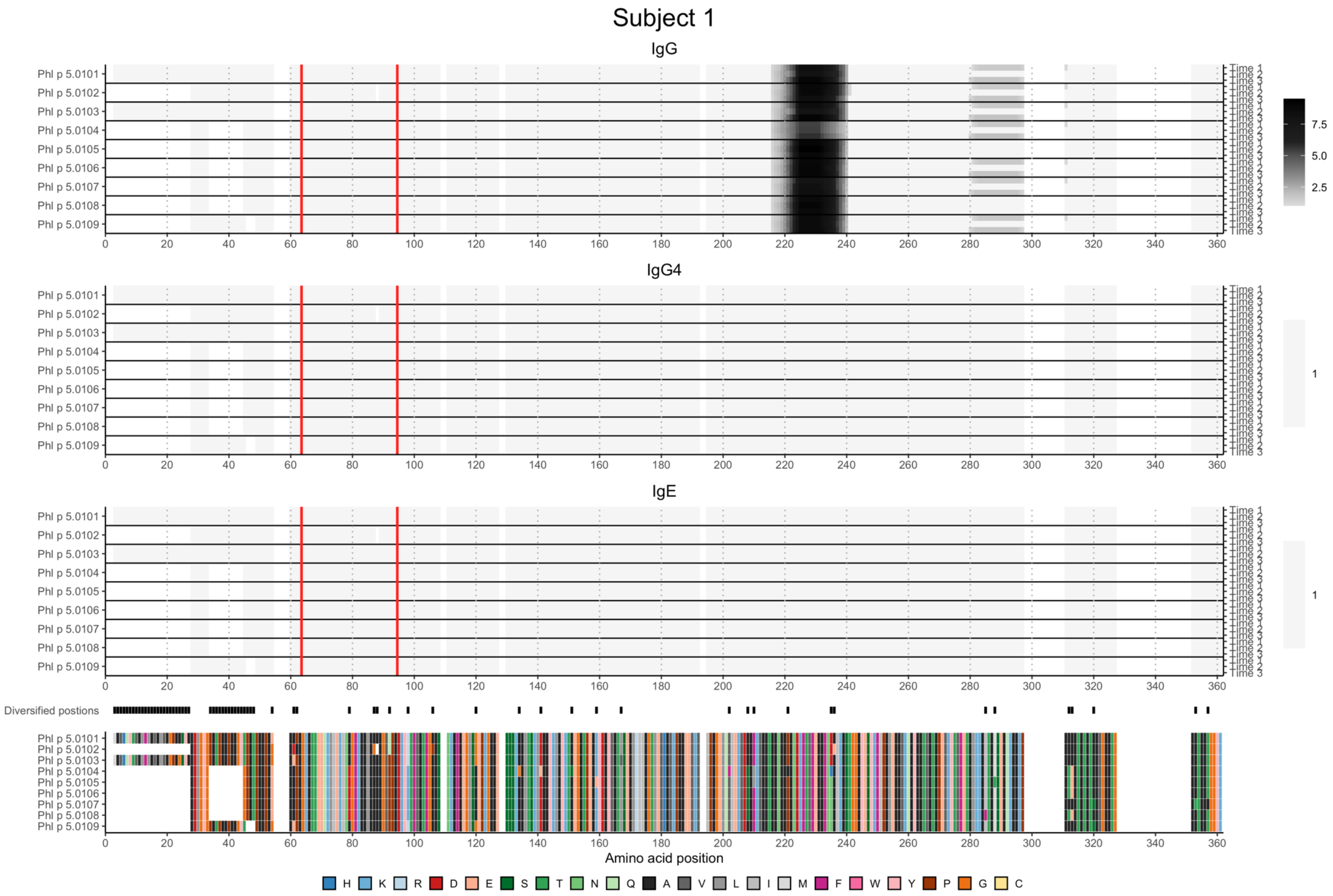

Subject 1
IgG
IgG4
IgE
Phl p 5.0101
Phl p 5.0102
Phl p 5.0103
Phl p 5.0104
Phl p 5.0105
Phl p 5.0106
Phl p 5.0107
Phl p 5.0108
Phl p 5.0109
Time 1
Time 2
Time 3
7.5
5.0
2.5
1
Diversified postions
Amino acid position
H K R D E S T N Q A V L I M F W Y P G C

Subject 2

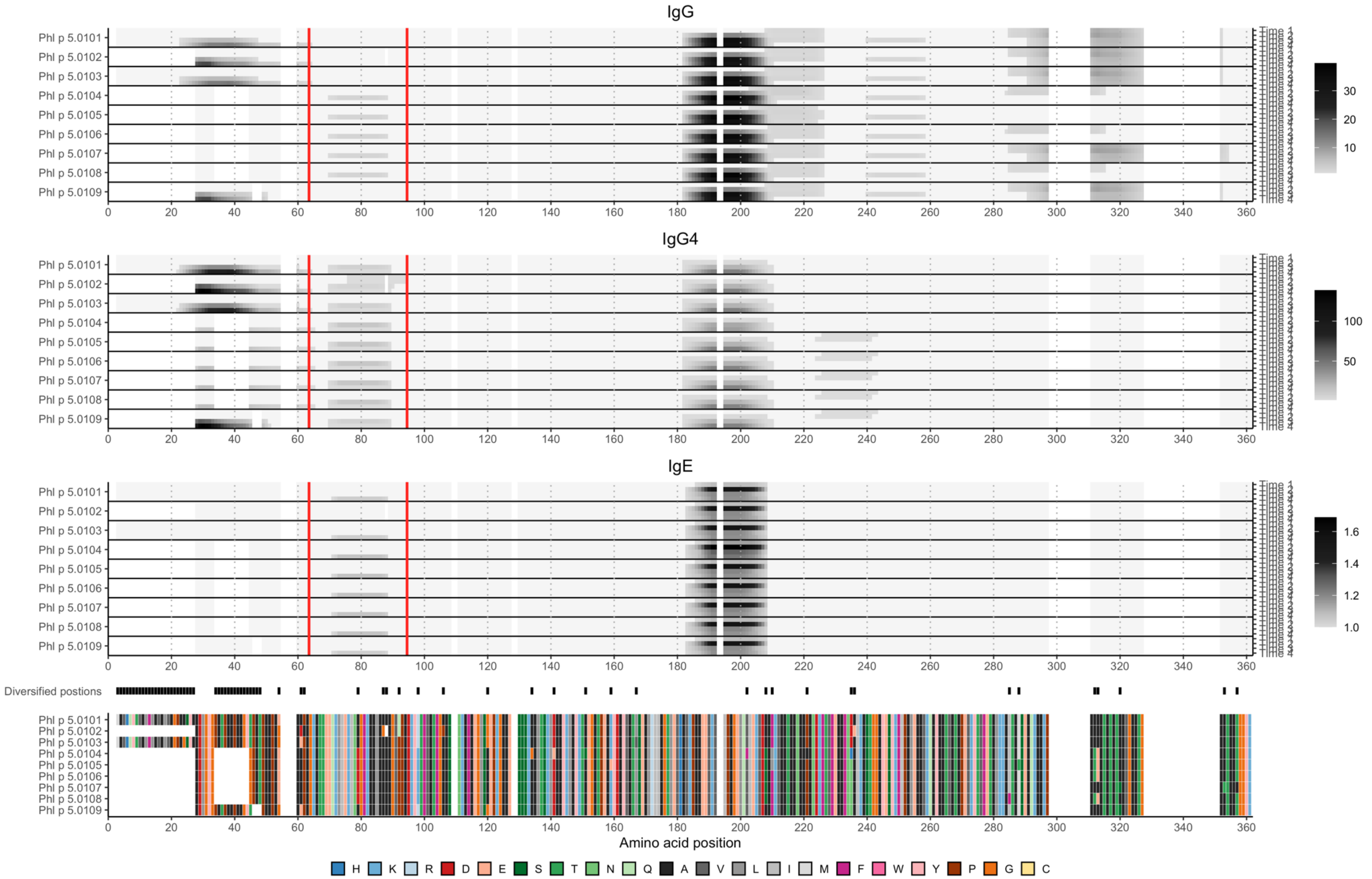

IgG
IgG4
IgE
Phl p 5.0101
Phl p 5.0102
Phl p 5.0103
Phl p 5.0104
Phl p 5.0105
Phl p 5.0106
Phl p 5.0107
Phl p 5.0108
Phl p 5.0109
0 20 40 60 80 100 120 140 160 180 200 220 240 260 280 300 320 340 360
30 20 10
100 50
1.6 1.4 1.2 1.0
Diversified postions
Amino acid position
H K R D E S T N Q A V L I M F W Y P G C

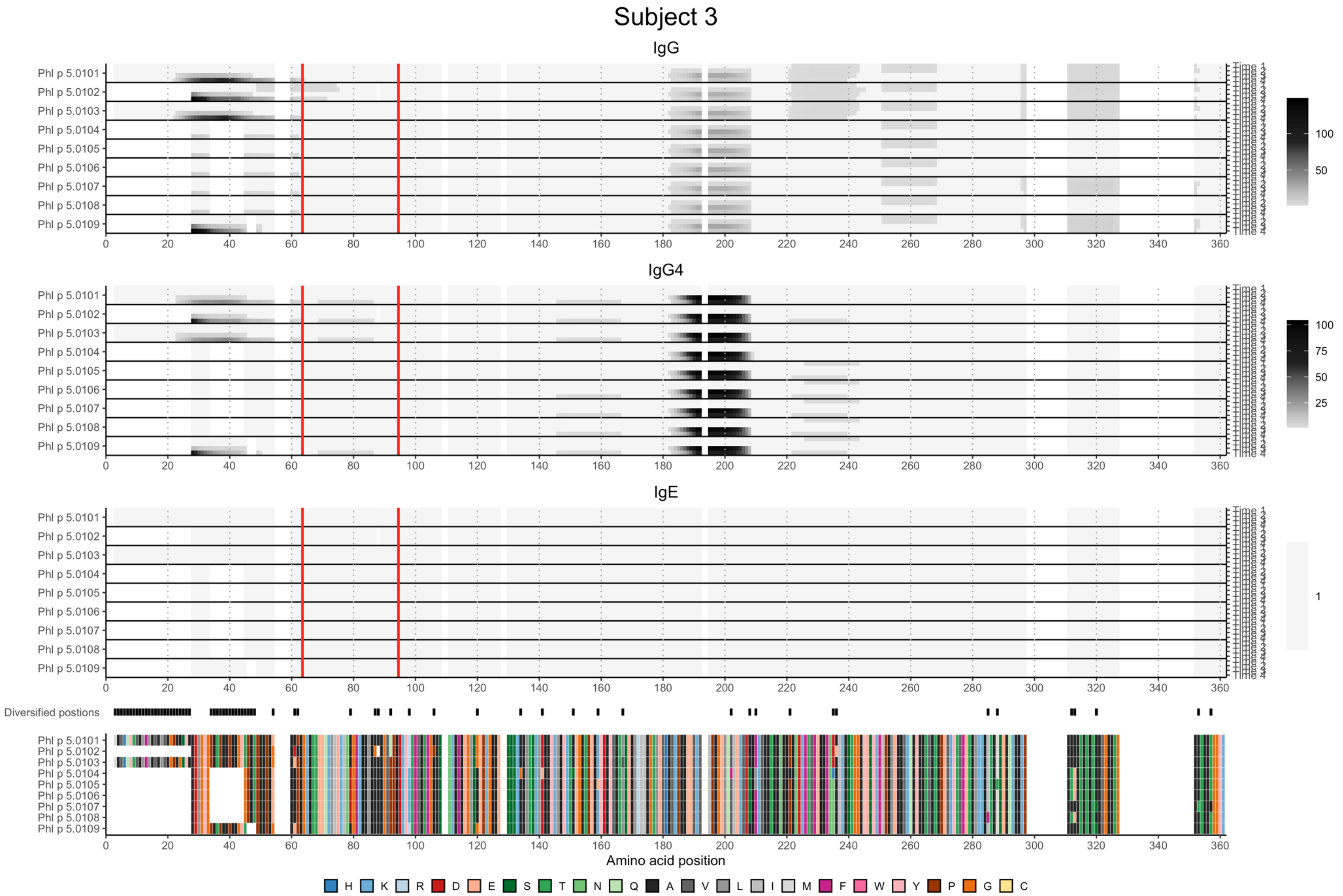

Subject 3
IgG
IgG4
IgE
Phl p 5.0101
Phl p 5.0102
Phl p 5.0103
Phl p 5.0104
Phl p 5.0105
Phl p 5.0106
Phl p 5.0107
Phl p 5.0108
Phl p 5.0109
0 20 40 60 80 100 120 140 160 180 200 220 240 260 280 300 320 340 360
100
50
100
75
50
25
1
Diversified postions
Amino acid position
H K R D E S T N Q A V L I M F W Y P G C

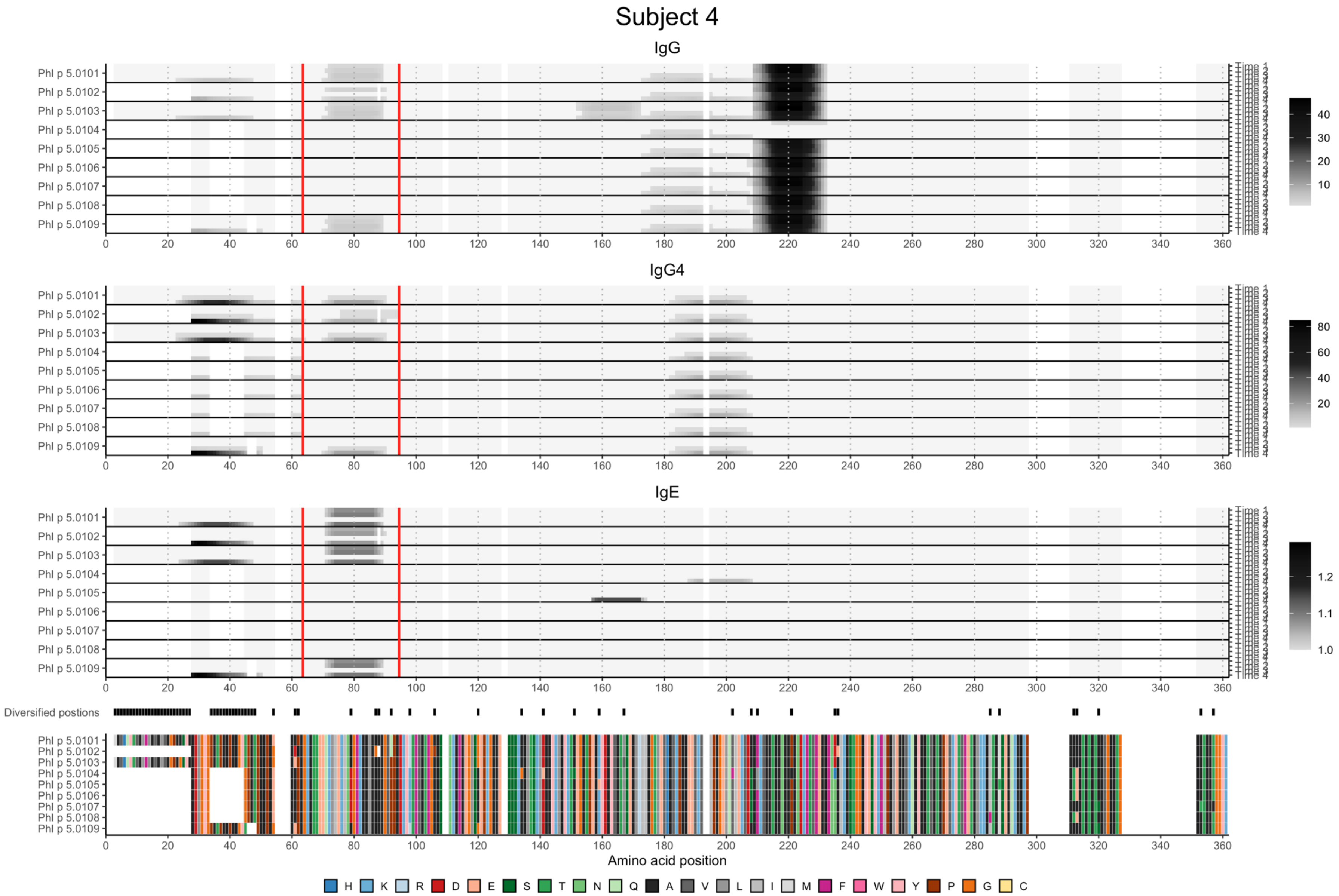
Subject 4
IgG
IgG4
IgE
Phl p 5.0101
Phl p 5.0102
Phl p 5.0103
Phl p 5.0104
Phl p 5.0105
Phl p 5.0106
Phl p 5.0107
Phl p 5.0108
Phl p 5.0109
Diversified postions
Amino acid position
H K R D E S T N Q A V L I M F W Y P G C

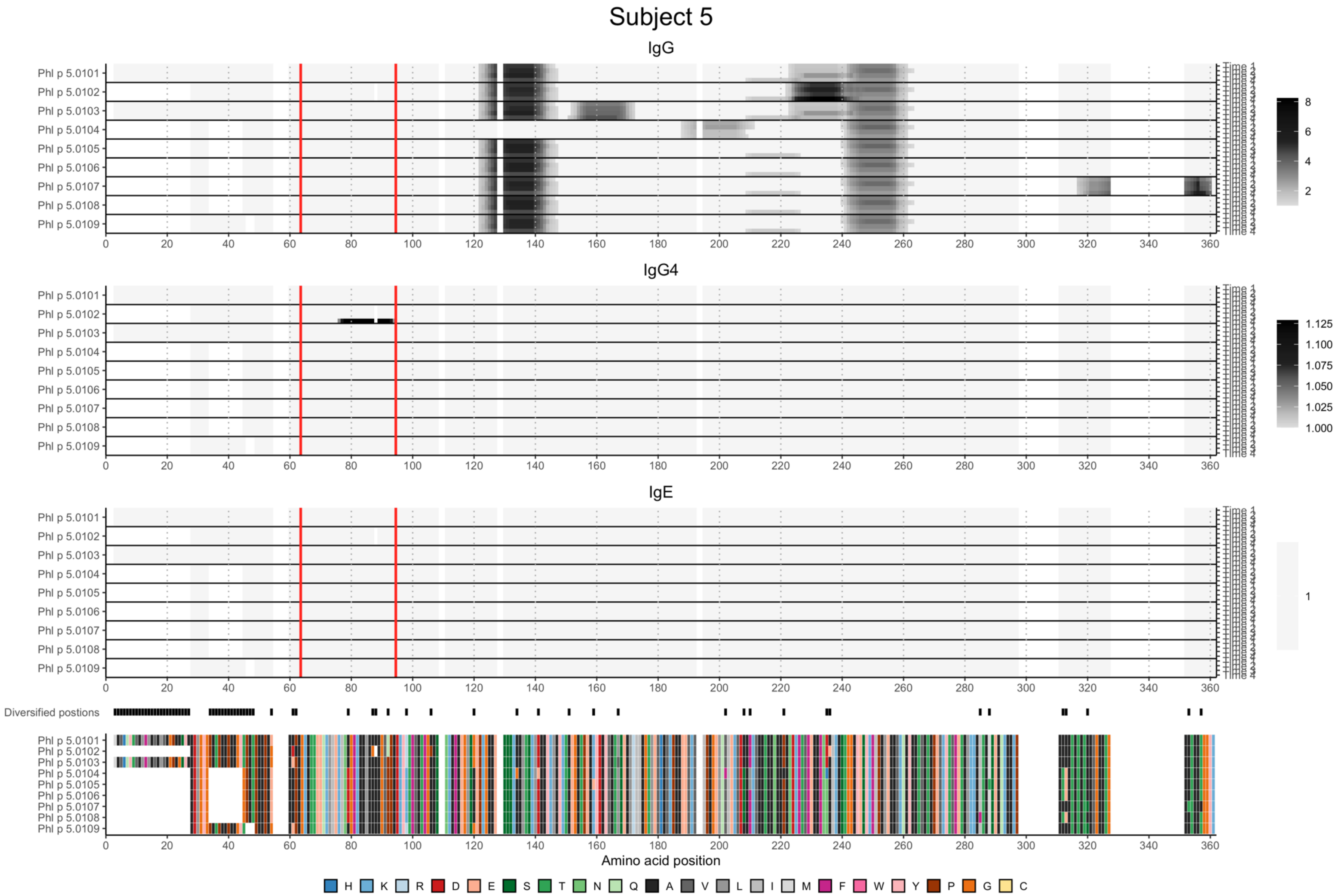

Subject 5
IgG
IgG4
IgE
Phl p 5.0101
Phl p 5.0102
Phl p 5.0103
Phl p 5.0104
Phl p 5.0105
Phl p 5.0106
Phl p 5.0107
Phl p 5.0108
Phl p 5.0109
Diversified postions
Amino acid position
0 20 40 60 80 100 120 140 160 180 200 220 240 260 280 300 320 340 360
2 4 6 8
1.000 1.025 1.050 1.075 1.100 1.125
1
H K R D E S T N Q A V L I M F W Y P G C

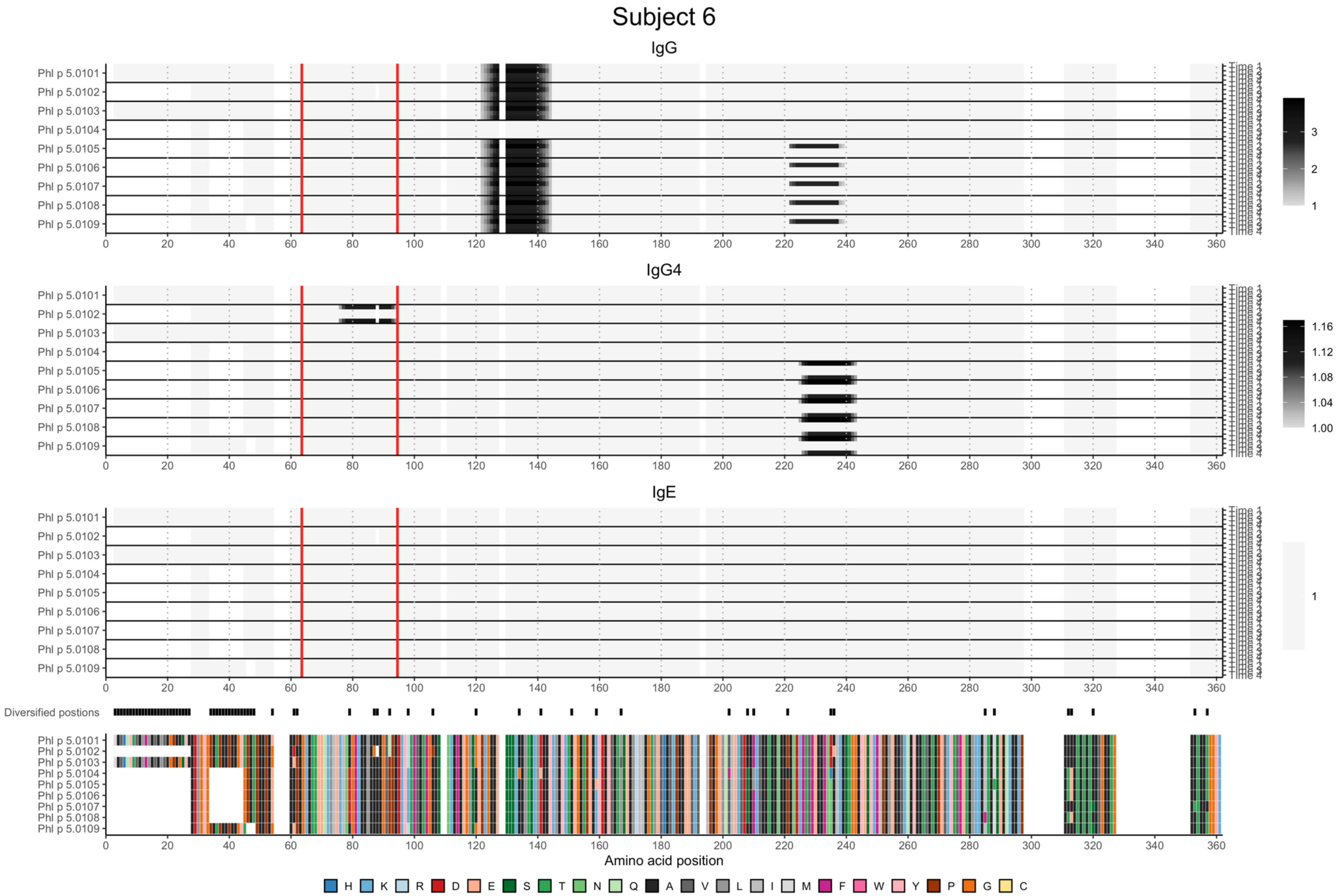

Subject 6
IgG
IgG4
IgE
Phl p 5.0101
Phl p 5.0102
Phl p 5.0103
Phl p 5.0104
Phl p 5.0105
Phl p 5.0106
Phl p 5.0107
Phl p 5.0108
Phl p 5.0109
Diversified postions
Amino acid position
H K R D E S T N Q A V L I M F W Y P G C

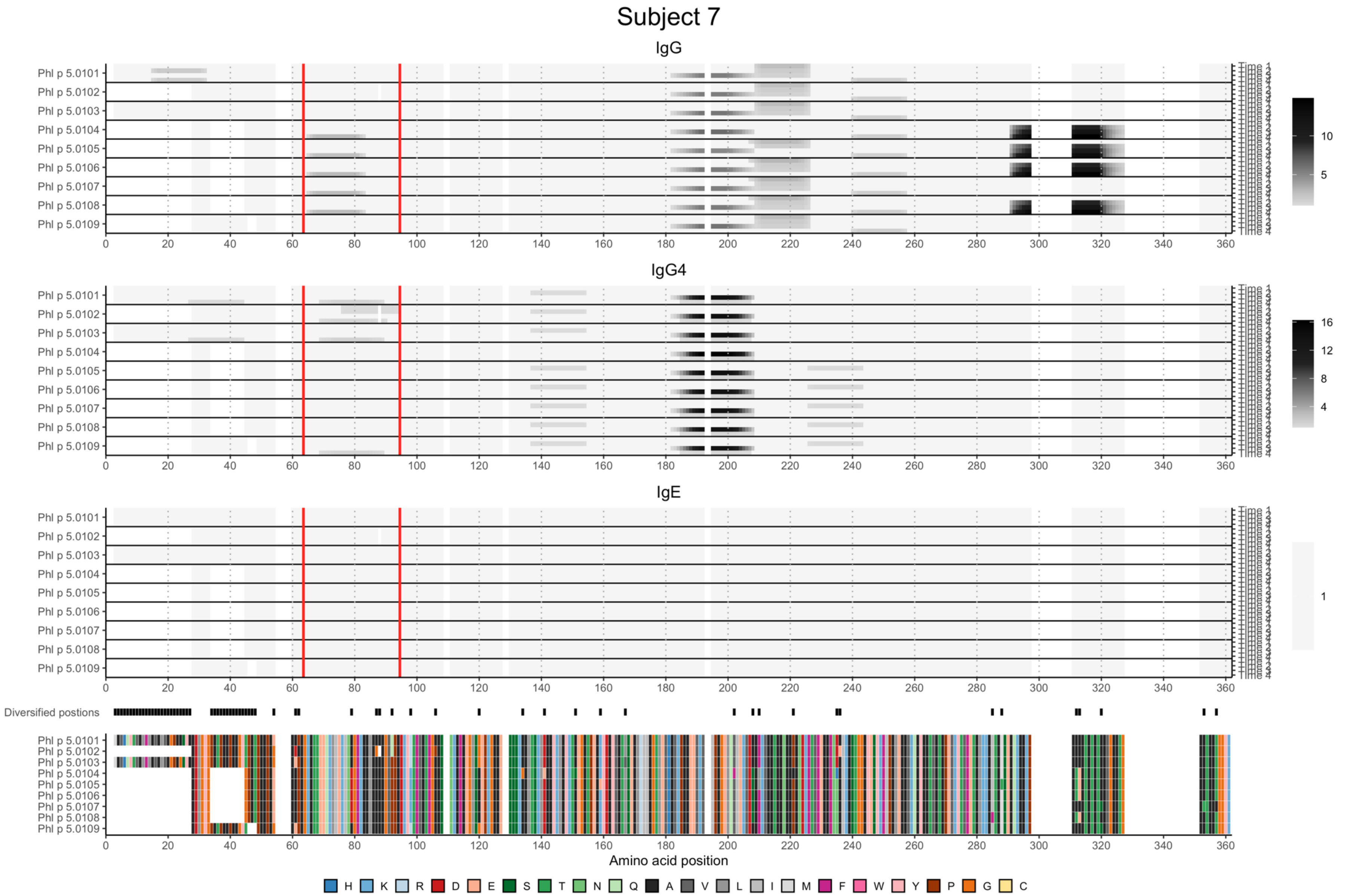
Subject 7
IgG
IgG4
IgE
Phl p 5.0101
Phl p 5.0102
Phl p 5.0103
Phl p 5.0104
Phl p 5.0105
Phl p 5.0106
Phl p 5.0107
Phl p 5.0108
Phl p 5.0109
0 20 40 60 80 100 120 140 160 180 200 220 240 260 280 300 320 340 360
10
5
16
12
8
4
1
Diversified postions
Amino acid position
H K R D E S T N Q A V L I M F W Y P G C

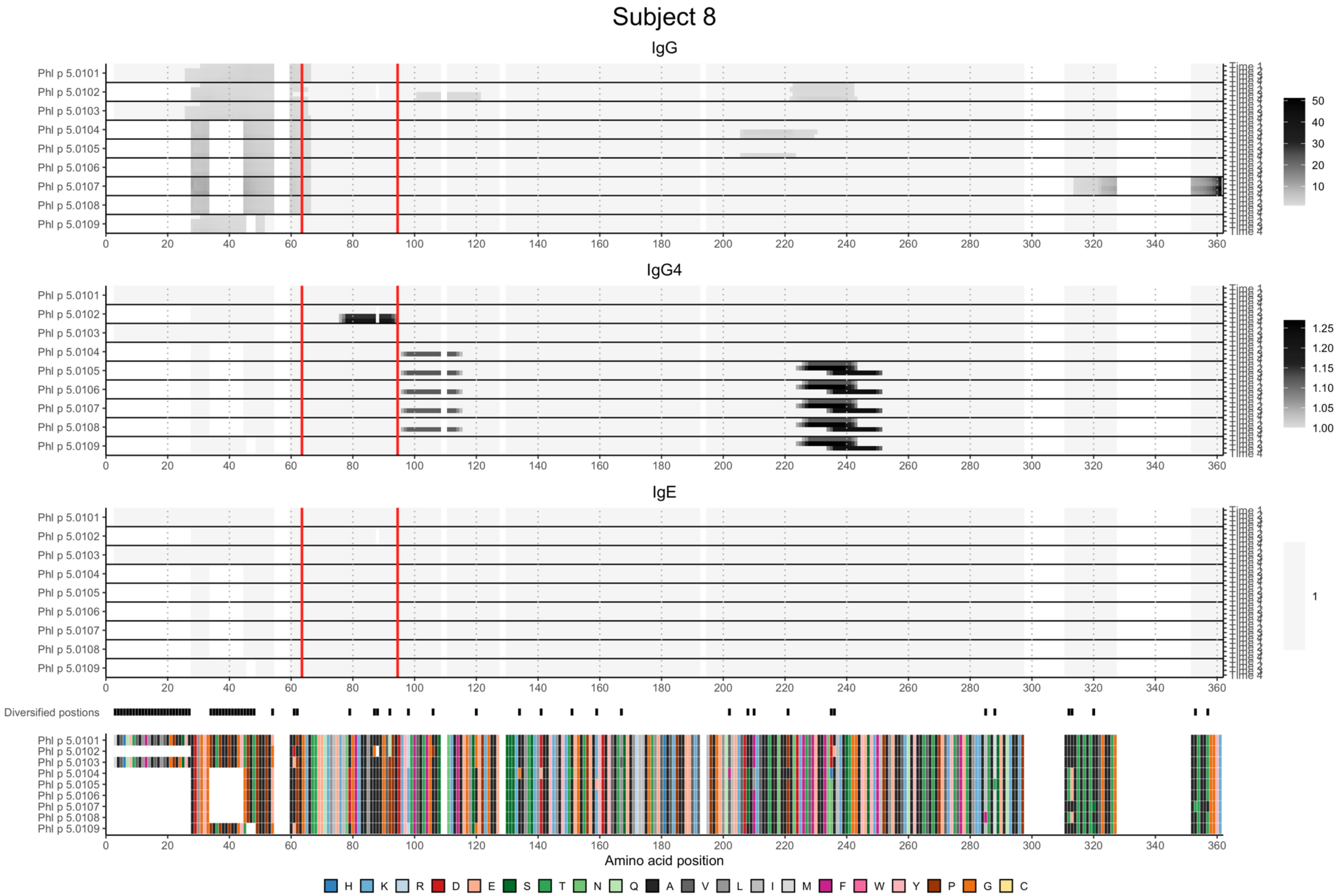

Subject 8
IgG
IgG4
IgE
Phl p 5.0101
Phl p 5.0102
Phl p 5.0103
Phl p 5.0104
Phl p 5.0105
Phl p 5.0106
Phl p 5.0107
Phl p 5.0108
Phl p 5.0109
0 20 40 60 80 100 120 140 160 180 200 220 240 260 280 300 320 340 360
50 40 30 20 10
1.25 1.20 1.15 1.10 1.05 1.00
1
Diversified postions
Amino acid position
H K R D E S T N Q A V L I M F W Y P G C

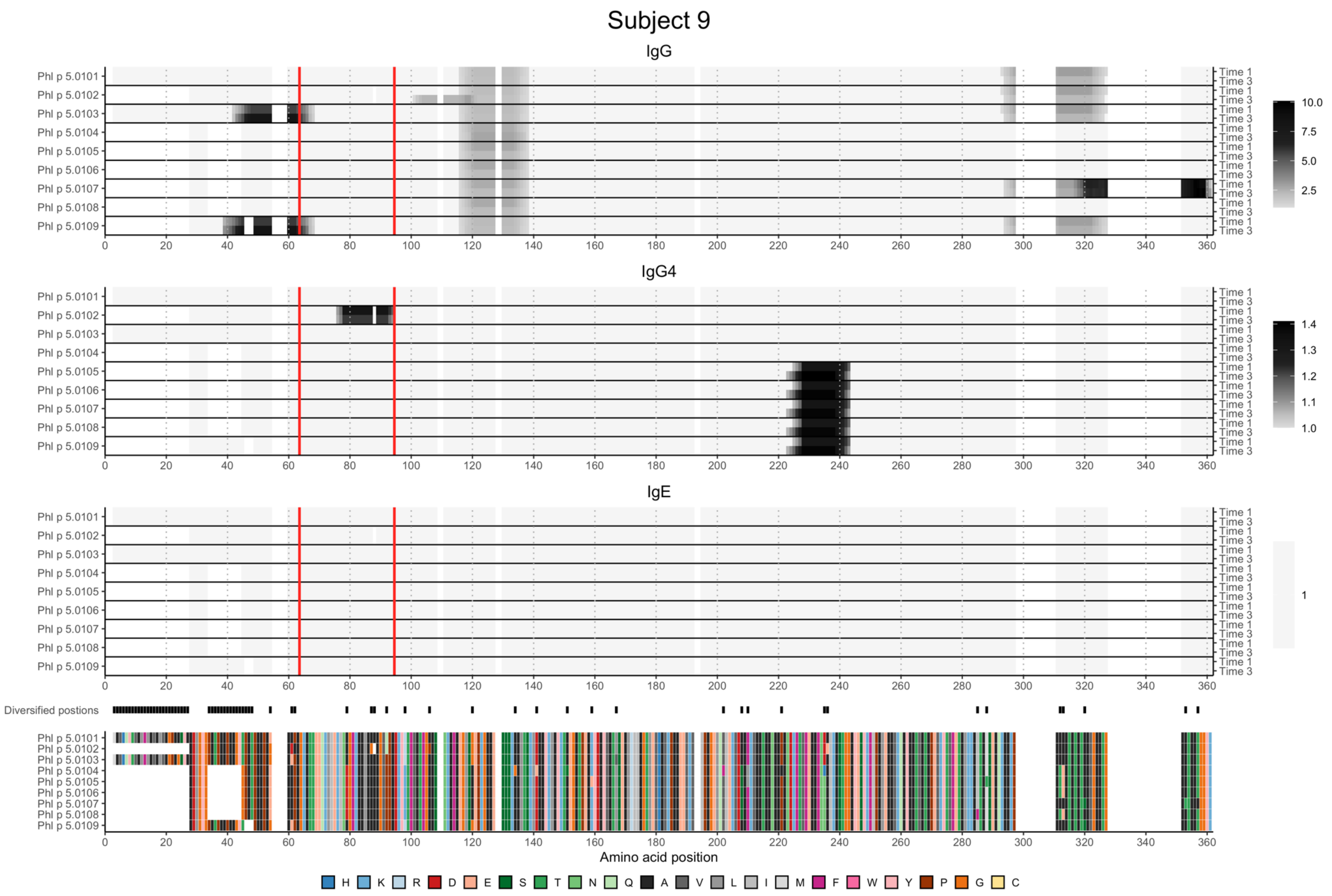

Subject 9
IgG
IgG4
IgE
Phl p 5.0101
Phl p 5.0102
Phl p 5.0103
Phl p 5.0104
Phl p 5.0105
Phl p 5.0106
Phl p 5.0107
Phl p 5.0108
Phl p 5.0109
Time 1
Time 3
10.0
7.5
5.0
2.5
1.4
1.3
1.2
1.1
1.0
1
Diversified postions
Amino acid position
H K R D E S T N Q A V L I M F W Y P G C

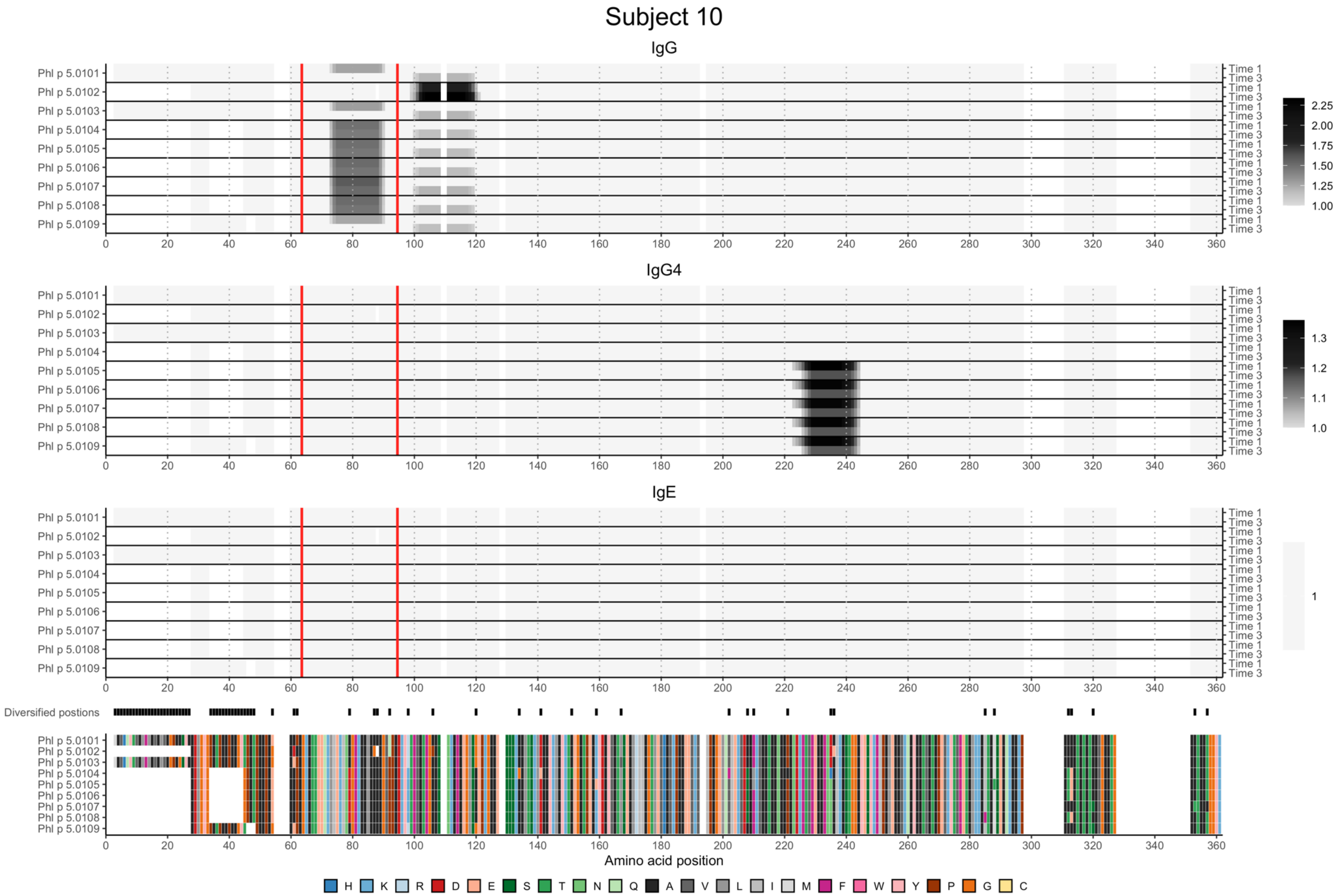

Subject 10
IgG
IgG4
IgE
Phl p 5.0101
Phl p 5.0102
Phl p 5.0103
Phl p 5.0104
Phl p 5.0105
Phl p 5.0106
Phl p 5.0107
Phl p 5.0108
Phl p 5.0109
Time 1
Time 3
2.25
2.00
1.75
1.50
1.25
1.00
1.3
1.2
1.1
1.0
1
Diversified postions
Amino acid position
H K R D E S T N Q A V L I M F W Y P G C

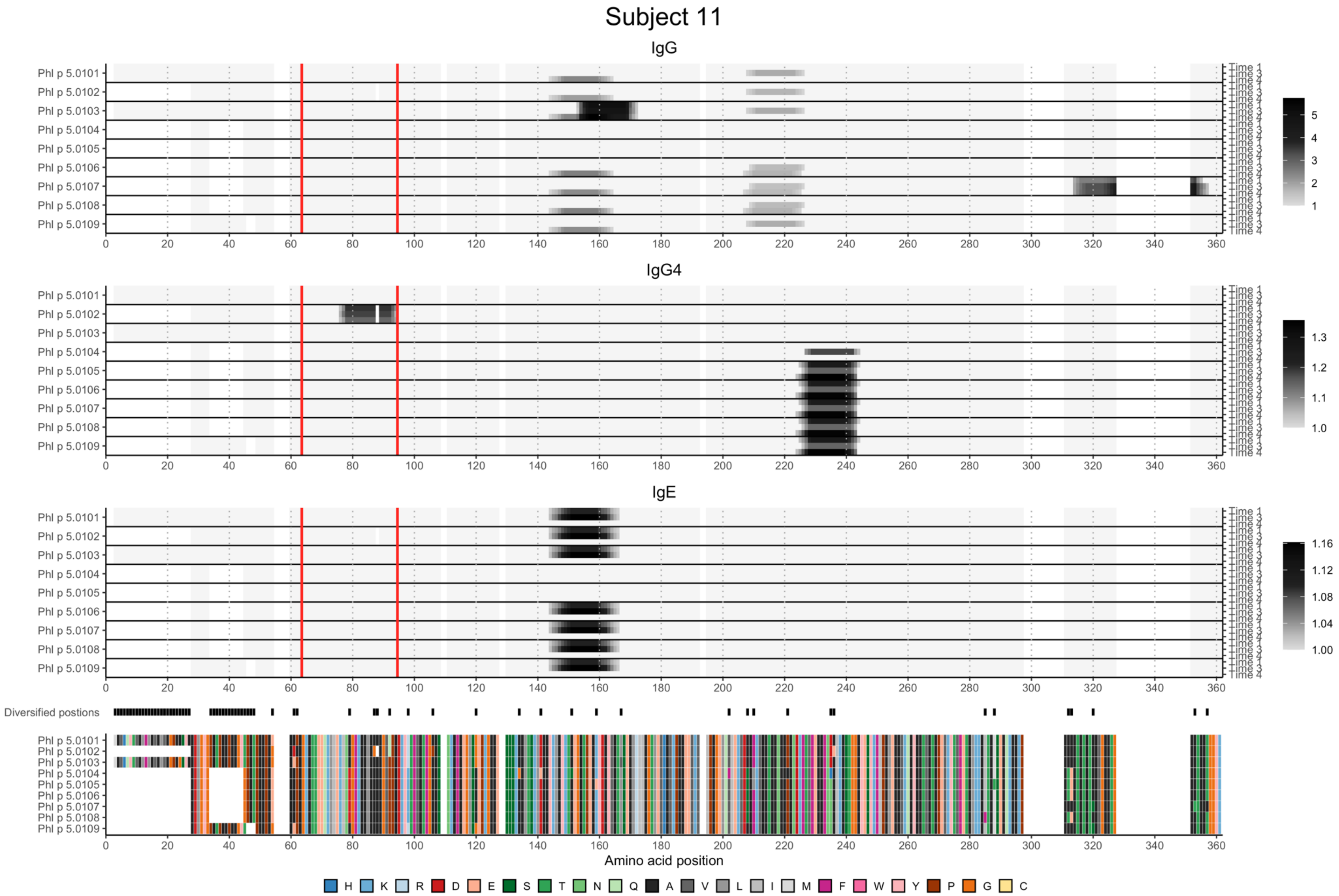

Subject 11
IgG
IgG4
IgE
Phl p 5.0101
Phl p 5.0102
Phl p 5.0103
Phl p 5.0104
Phl p 5.0105
Phl p 5.0106
Phl p 5.0107
Phl p 5.0108
Phl p 5.0109
Diversified postions
Amino acid position
H K R D E S T N Q A V L I M F W Y P G C

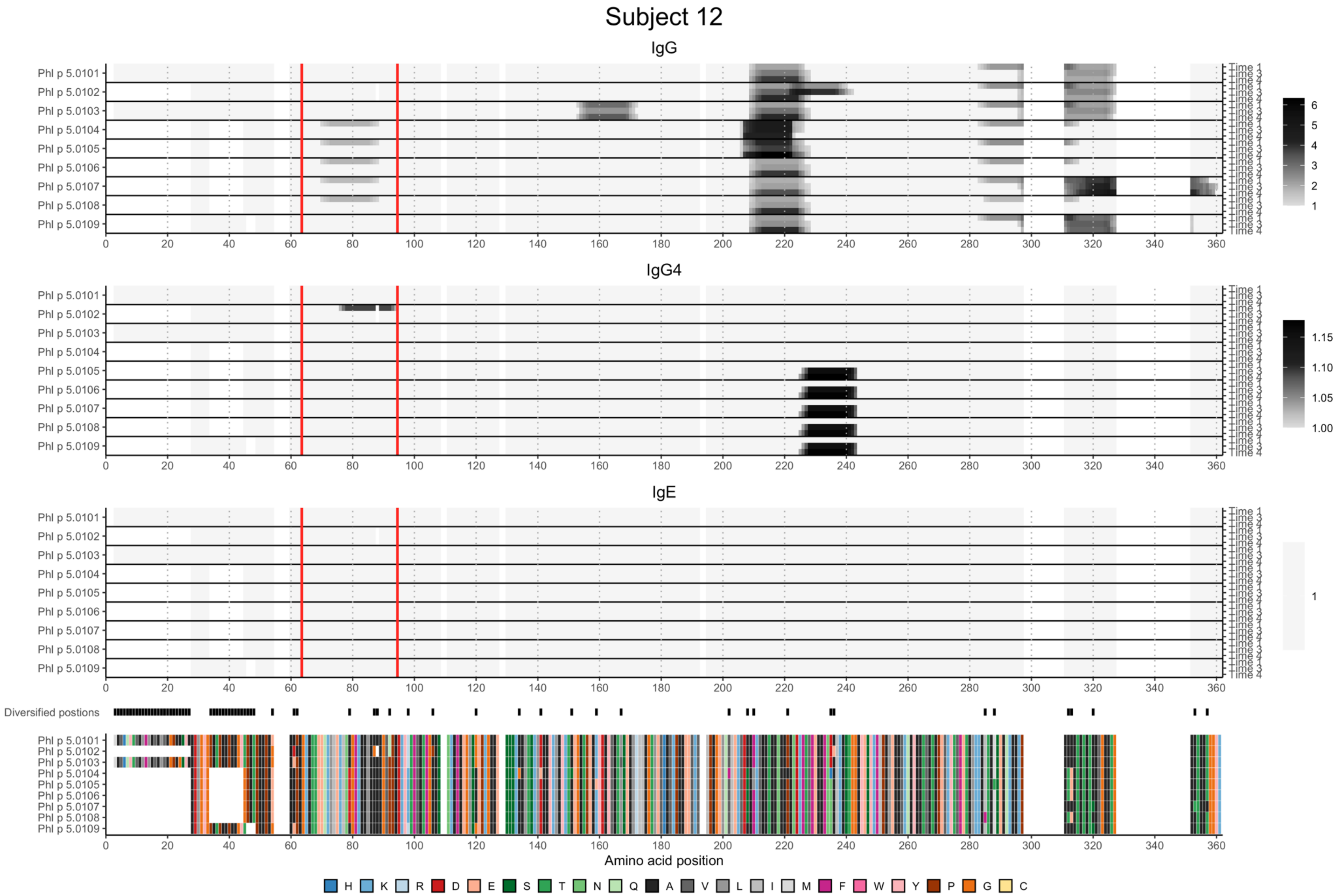

Subject 12
IgG
IgG4
IgE
Phl p 5.0101
Phl p 5.0102
Phl p 5.0103
Phl p 5.0104
Phl p 5.0105
Phl p 5.0106
Phl p 5.0107
Phl p 5.0108
Phl p 5.0109
0 20 40 60 80 100 120 140 160 180 200 220 240 260 280 300 320 340 360
6 5 4 3 2 1
1.15 1.10 1.05 1.00
1
Diversified postions
Amino acid position
H K R D E S T N Q A V L I M F W Y P G C

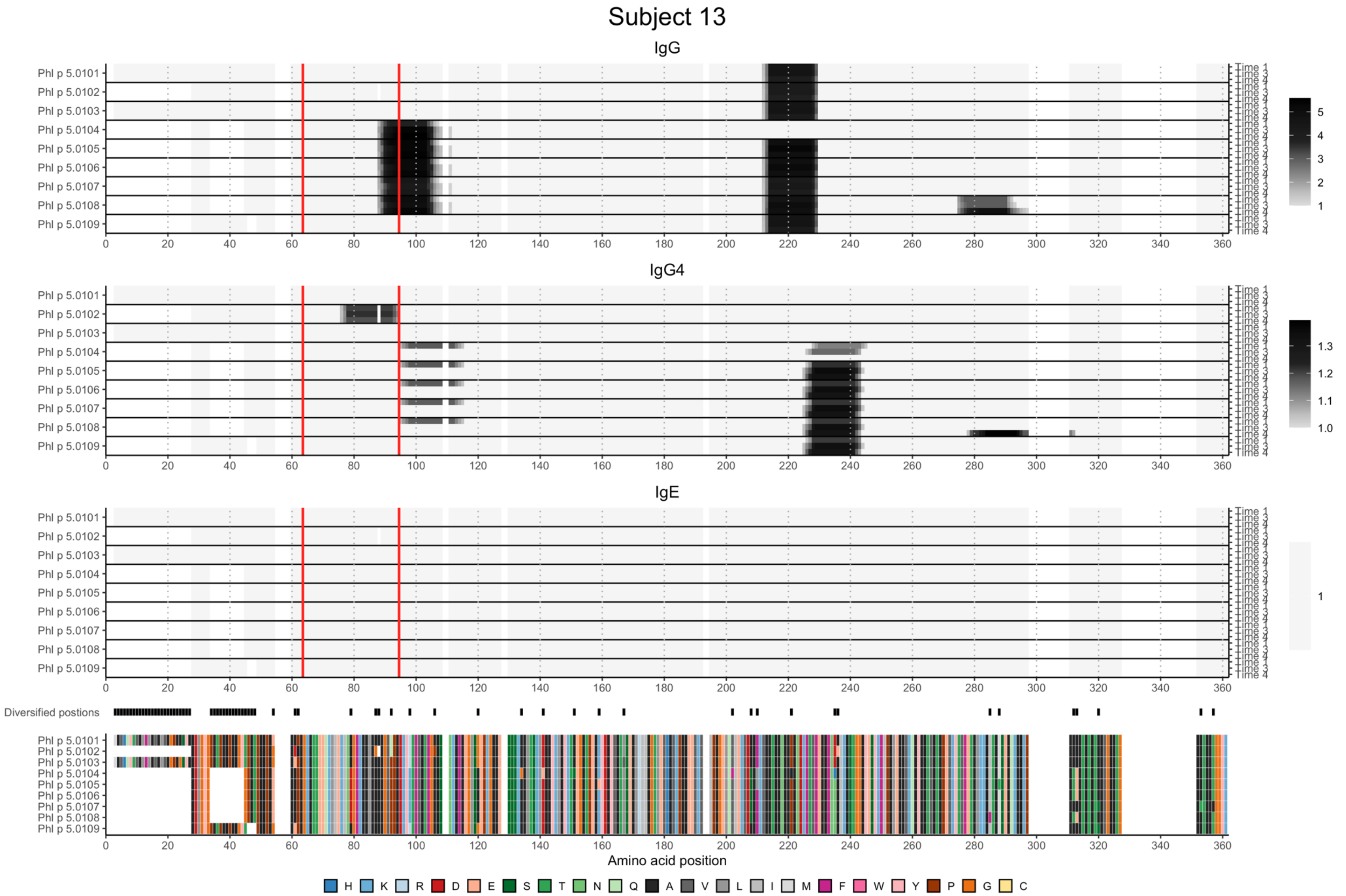

Subject 13
IgG
IgG4
IgE
Phl p 5.0101
Phl p 5.0102
Phl p 5.0103
Phl p 5.0104
Phl p 5.0105
Phl p 5.0106
Phl p 5.0107
Phl p 5.0108
Phl p 5.0109
Diversified postions
Amino acid position
H K R D E S T N Q A V L I M F W Y P G C

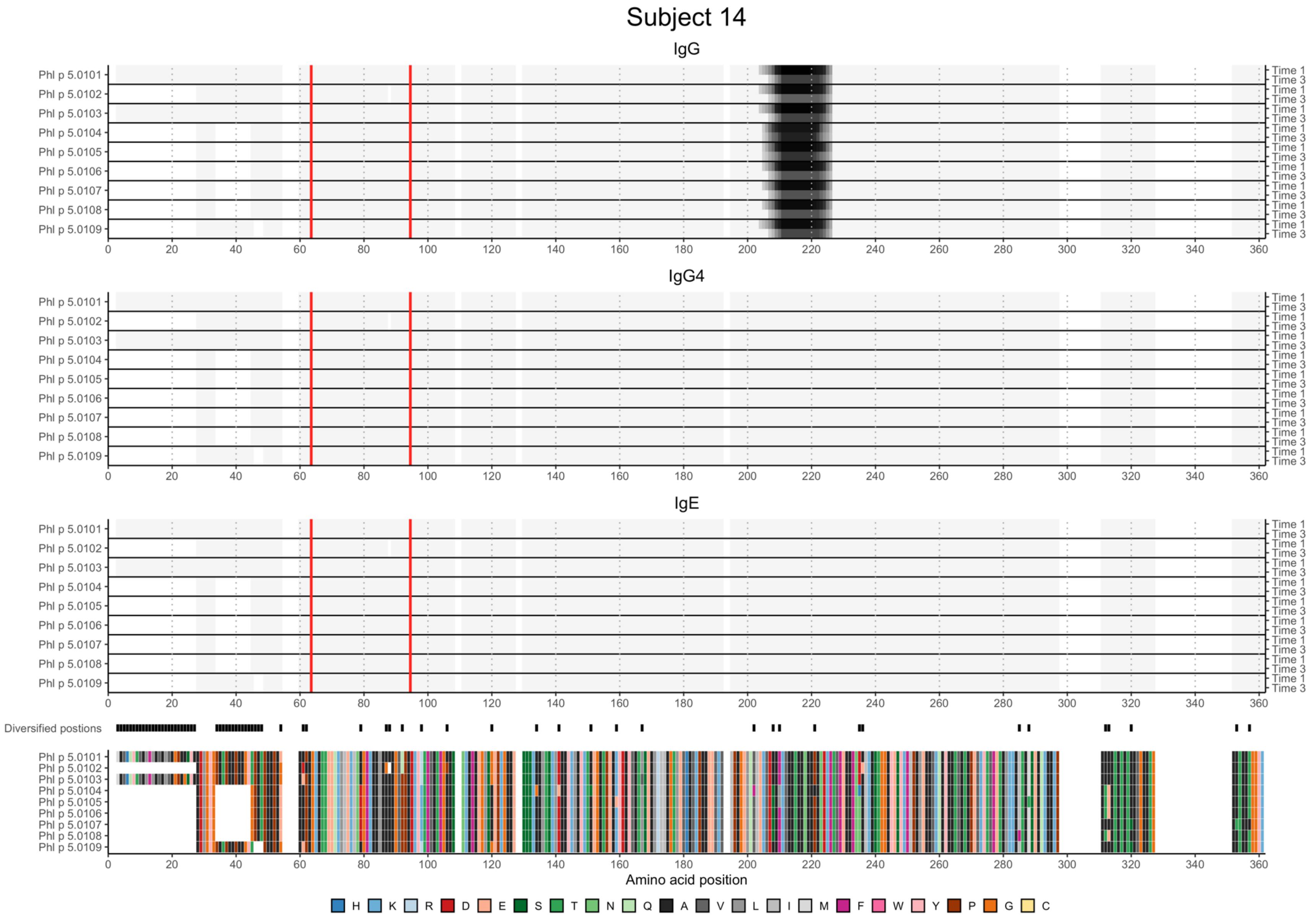

Subject 14
IgG
IgG4
IgE
Phl p 5.0101
Phl p 5.0102
Phl p 5.0103
Phl p 5.0104
Phl p 5.0105
Phl p 5.0106
Phl p 5.0107
Phl p 5.0108
Phl p 5.0109
Time 1
Time 3
Diversified postions
Amino acid position
H K R D E S T N Q A V L I M F W Y P G C

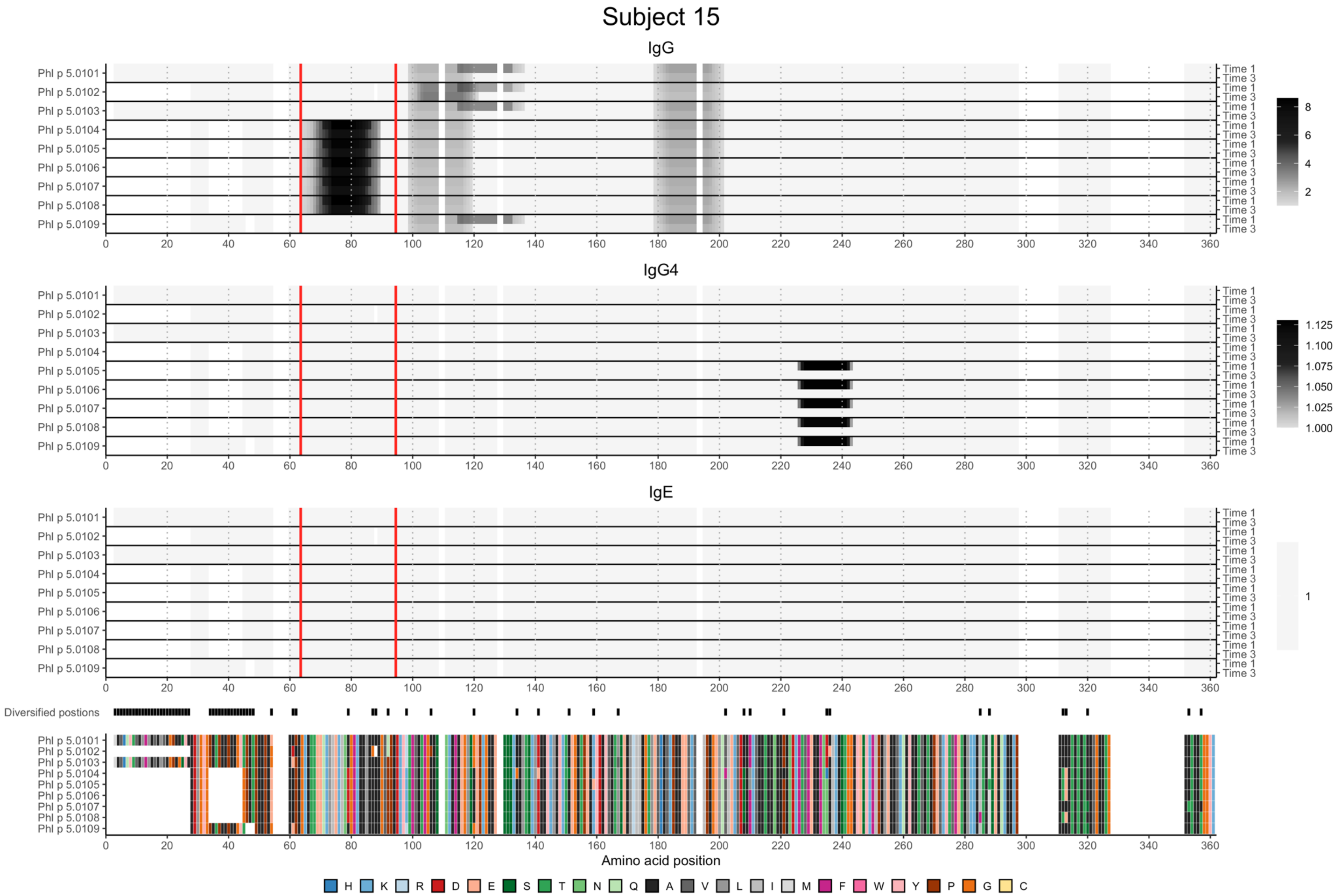

Subject 15
IgG
IgG4
IgE
Phl p 5.0101
Phl p 5.0102
Phl p 5.0103
Phl p 5.0104
Phl p 5.0105
Phl p 5.0106
Phl p 5.0107
Phl p 5.0108
Phl p 5.0109
Time 1
Time 3
Diversified postions
Amino acid position
H K R D E S T N Q A V L I M F W Y P G C

Supplementary Figure 10. Heatmap visualisation of IgG, IgG4, and IgE recognition of linear epitopes for Phl 5.01 allergen variants. Peptide signals have been used to estimate values on an amino acid level, as described. Amino acid values of 1 represents background, *i.e.*, amino acids that are not part of any reactive peptides. Epitope C have been highlighted in red. Sequence alignment of Phl p 5.01 allergen variants is also presented, with diversified residues marked above the alignment.
