# Supplementary material for: Linear Epitope Binding Patterns of Grass Pollen-Specific Antibodies in Allergy and in Response to Allergen-Specific Immunotherapy
Source: Front Allergy. 2022 Mar 31;3:859126. doi: 10.3389/falgy.2022.859126 (PMC9234942; doi:10.3389/falgy.2022.859126)
Supplement: Supplementary file 2 [file Data_Sheet_2.ZIP › Supplementary Figure 11.pdf]

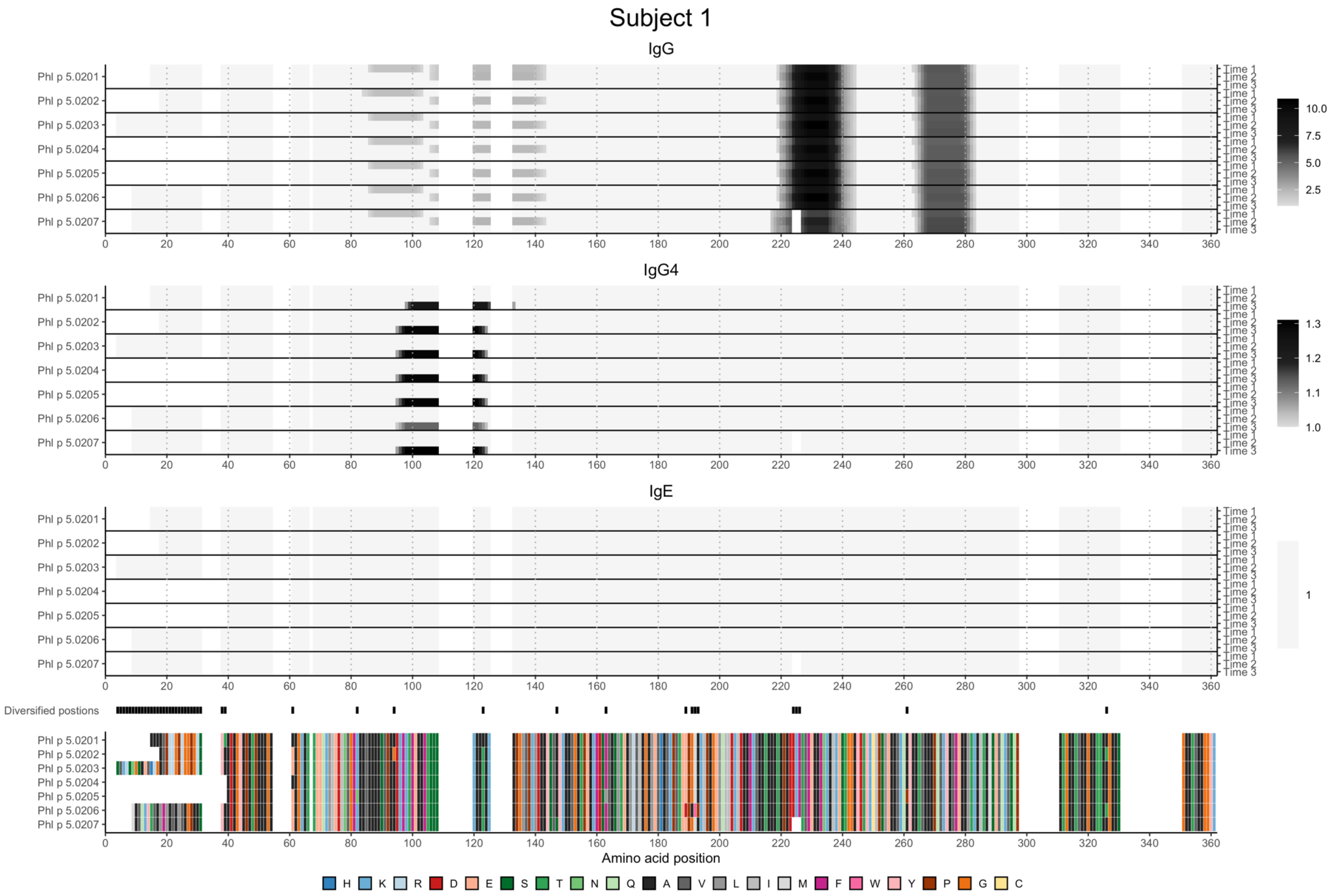

Subject 1
IgG
IgG4
IgE
Phl p 5.0201
Phl p 5.0202
Phl p 5.0203
Phl p 5.0204
Phl p 5.0205
Phl p 5.0206
Phl p 5.0207
Time 1
Time 2
Time 3
10.0
7.5
5.0
2.5
1.3
1.2
1.1
1.0
1
Diversified postions
Amino acid position
H K R D E S T N Q A V L I M F W Y P G C

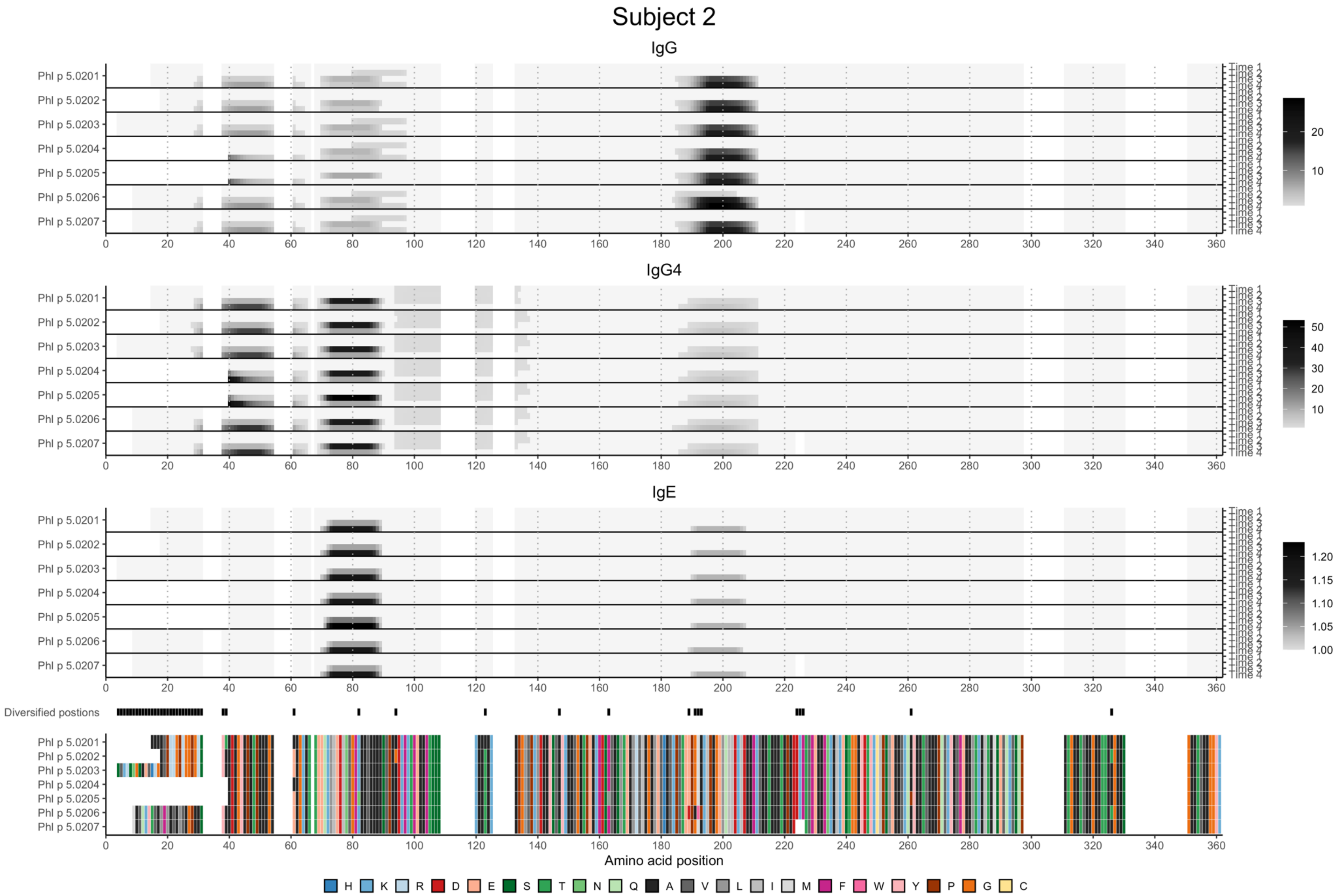

Subject 2
IgG
IgG4
IgE
Phl p 5.0201
Phl p 5.0202
Phl p 5.0203
Phl p 5.0204
Phl p 5.0205
Phl p 5.0206
Phl p 5.0207
Time 1
Time 2
Time 3
Time 4
Diversified postions
Amino acid position
H K R D E S T N Q A V L I M F W Y P G C

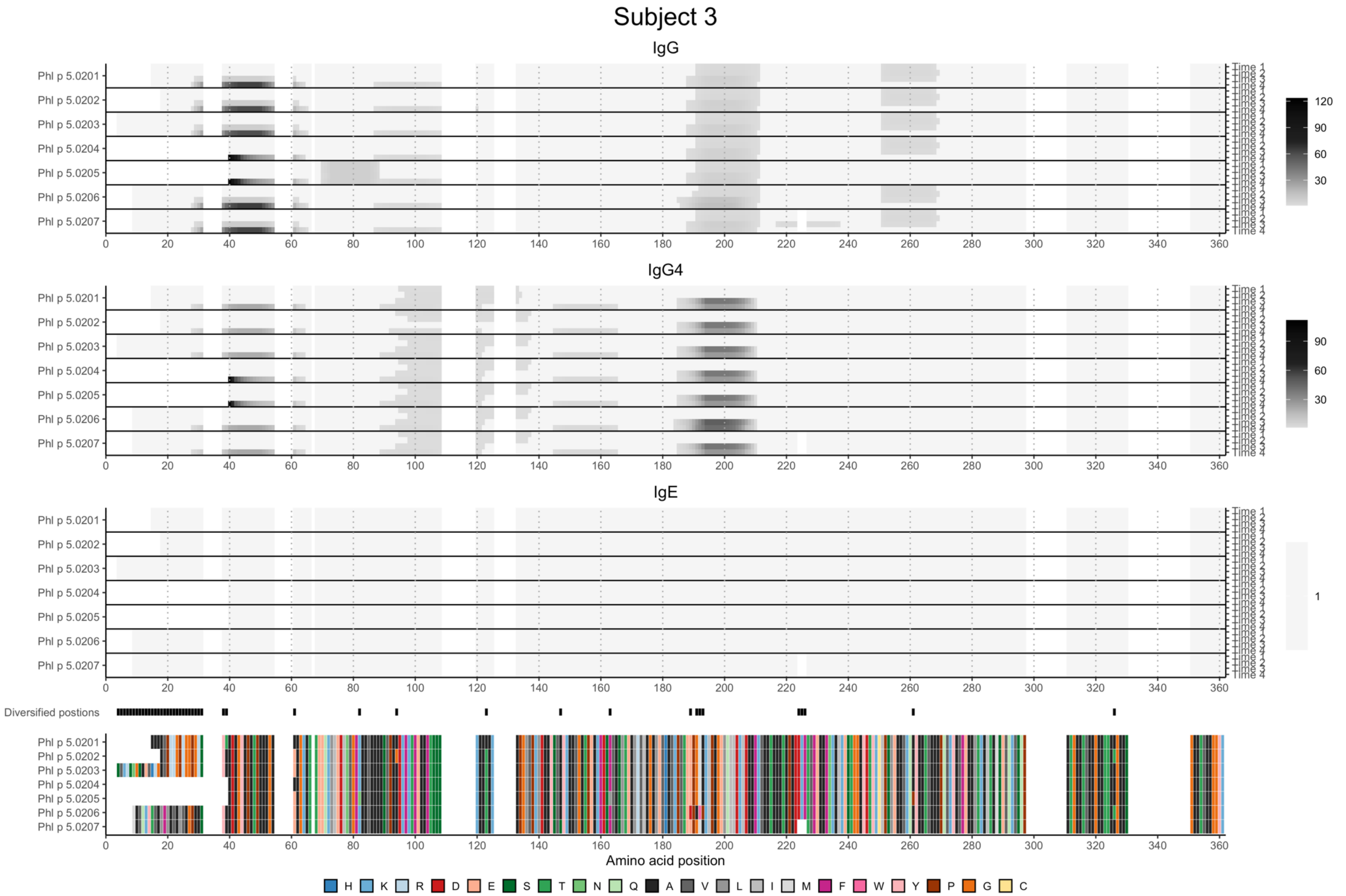
Subject 3
IgG
IgG4
IgE
Phl p 5.0201
Phl p 5.0202
Phl p 5.0203
Phl p 5.0204
Phl p 5.0205
Phl p 5.0206
Phl p 5.0207
Time 1
Time 2
Time 3
Time 4
120
90
60
30
1
0 20 40 60 80 100 120 140 160 180 200 220 240 260 280 300 320 340 360
Diversified postions
Amino acid position
H K R D E S T N Q A V L I M F W Y P G C

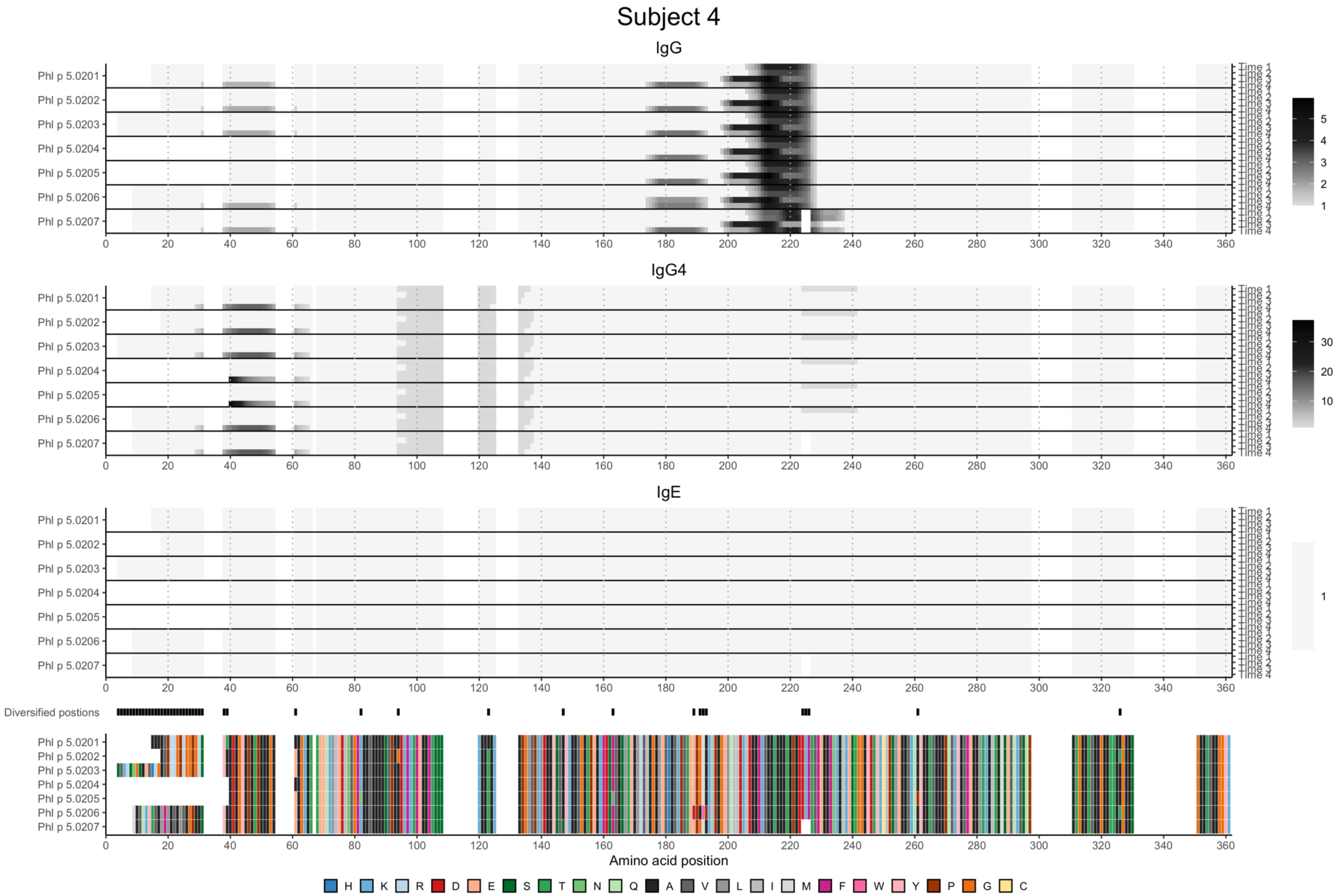
Subject 4
IgG
IgG4
IgE
Phl p 5.0201
Phl p 5.0202
Phl p 5.0203
Phl p 5.0204
Phl p 5.0205
Phl p 5.0206
Phl p 5.0207
Diversified postions
Amino acid position
H K R D E S T N Q A V L I M F W Y P G C

Subject 5

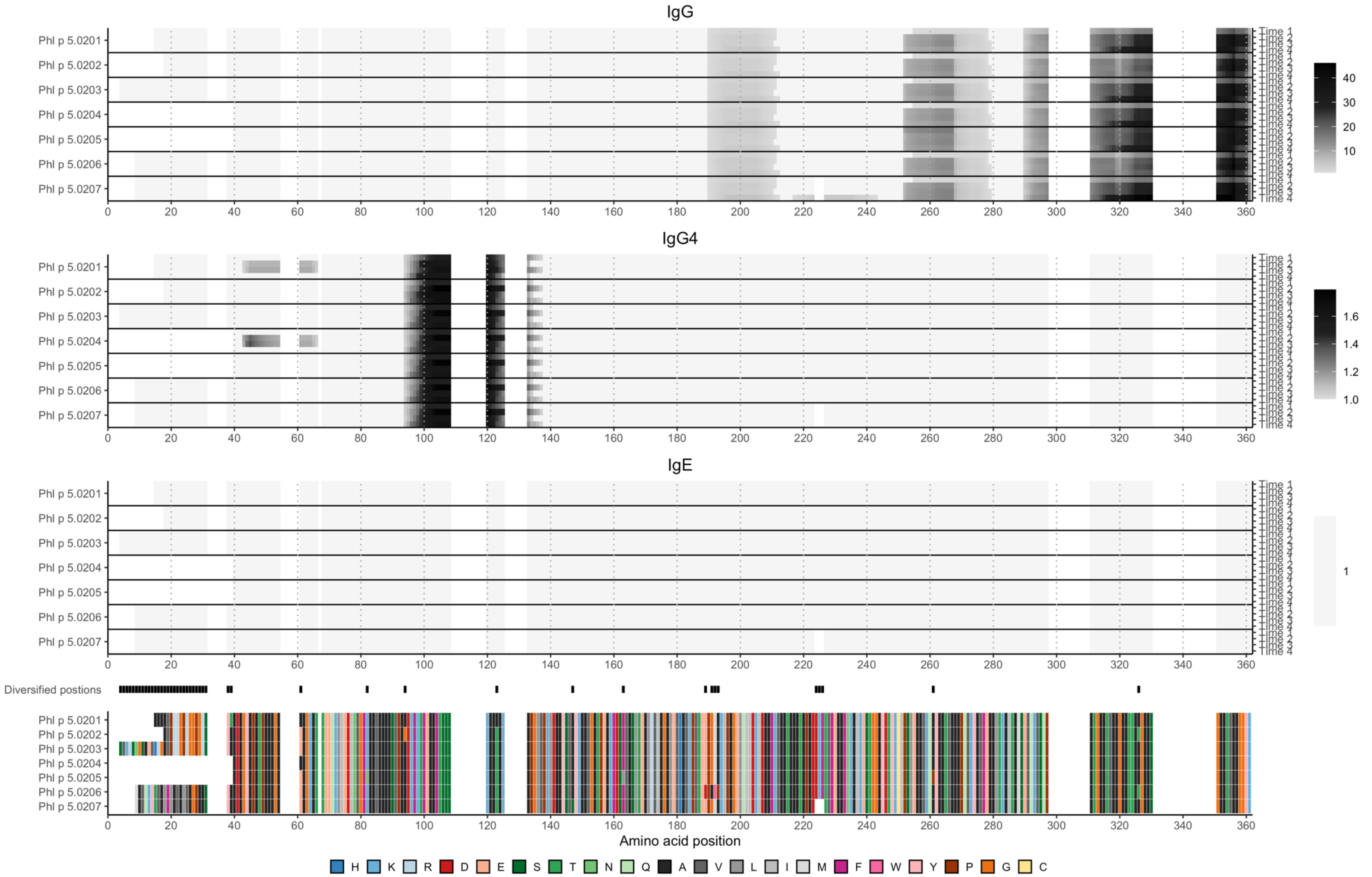

IgG
IgG4
IgE
Phl p 5.0201
Phl p 5.0202
Phl p 5.0203
Phl p 5.0204
Phl p 5.0205
Phl p 5.0206
Phl p 5.0207
Diversified postions
Amino acid position
H K R D E S T N Q A V L I M F W Y P G C

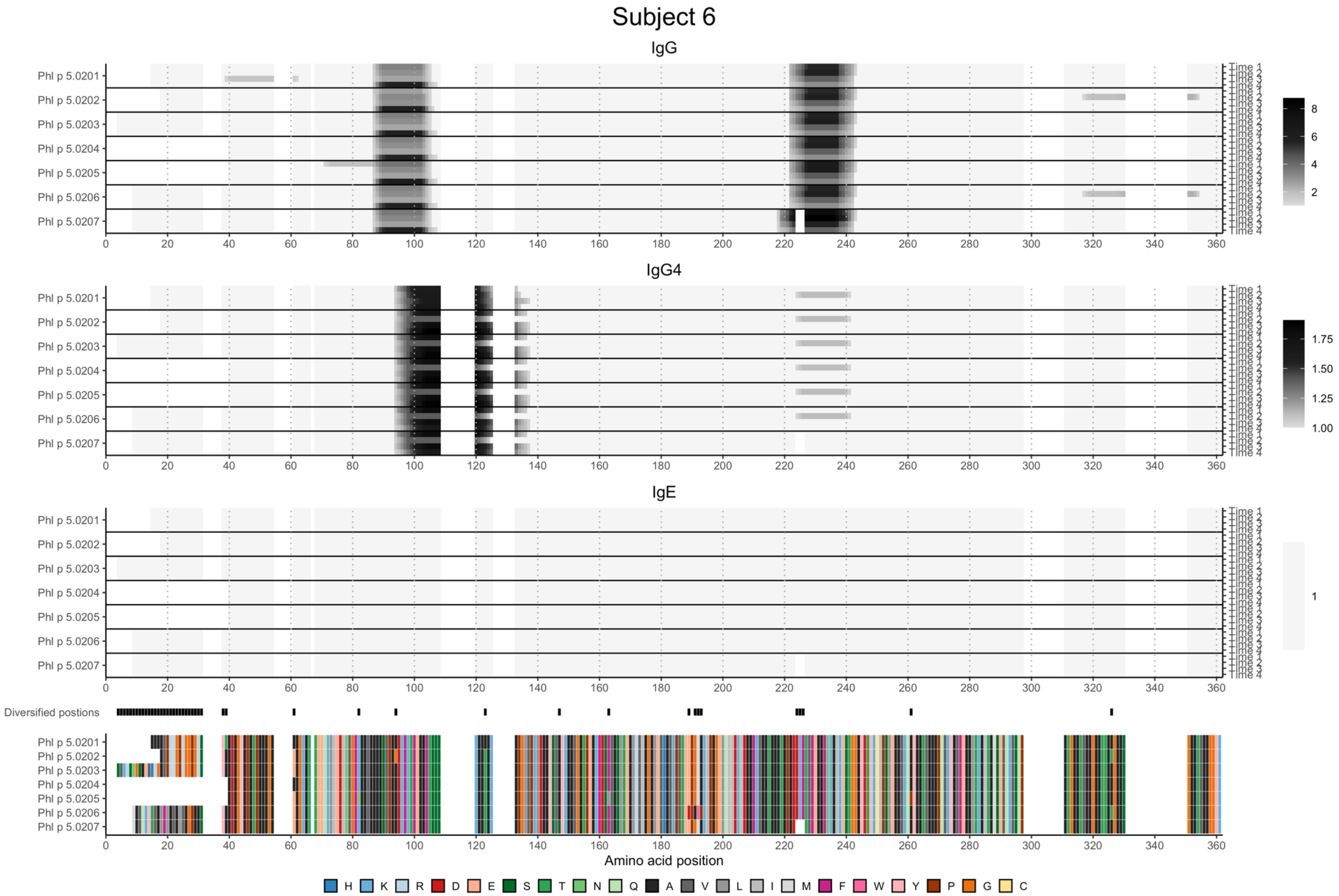
Subject 6
IgG
IgG4
IgE
Phl p 5.0201
Phl p 5.0202
Phl p 5.0203
Phl p 5.0204
Phl p 5.0205
Phl p 5.0206
Phl p 5.0207
Time 1
Time 2
Time 3
Time 4
Diversified postions
Amino acid position
H K R D E S T N Q A V L I M F W Y P G C

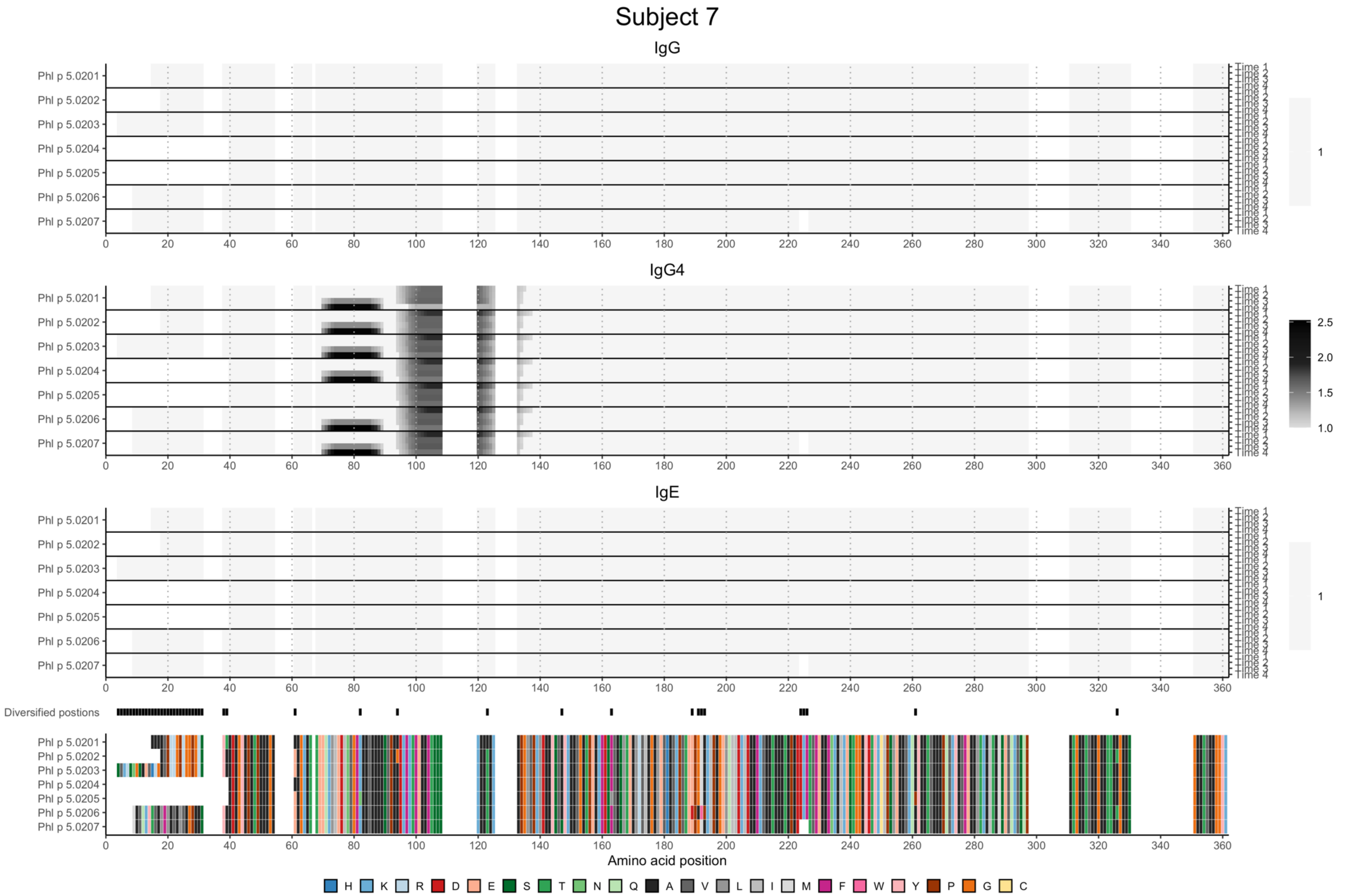
Subject 7
IgG
IgG4
IgE
Phl p 5.0201
Phl p 5.0202
Phl p 5.0203
Phl p 5.0204
Phl p 5.0205
Phl p 5.0206
Phl p 5.0207
Time 1
Time 2
Time 3
Time 4
1
2.5
2.0
1.5
1.0
Diversified postions
Amino acid position
H K R D E S T N Q A V L I M F W Y P G C

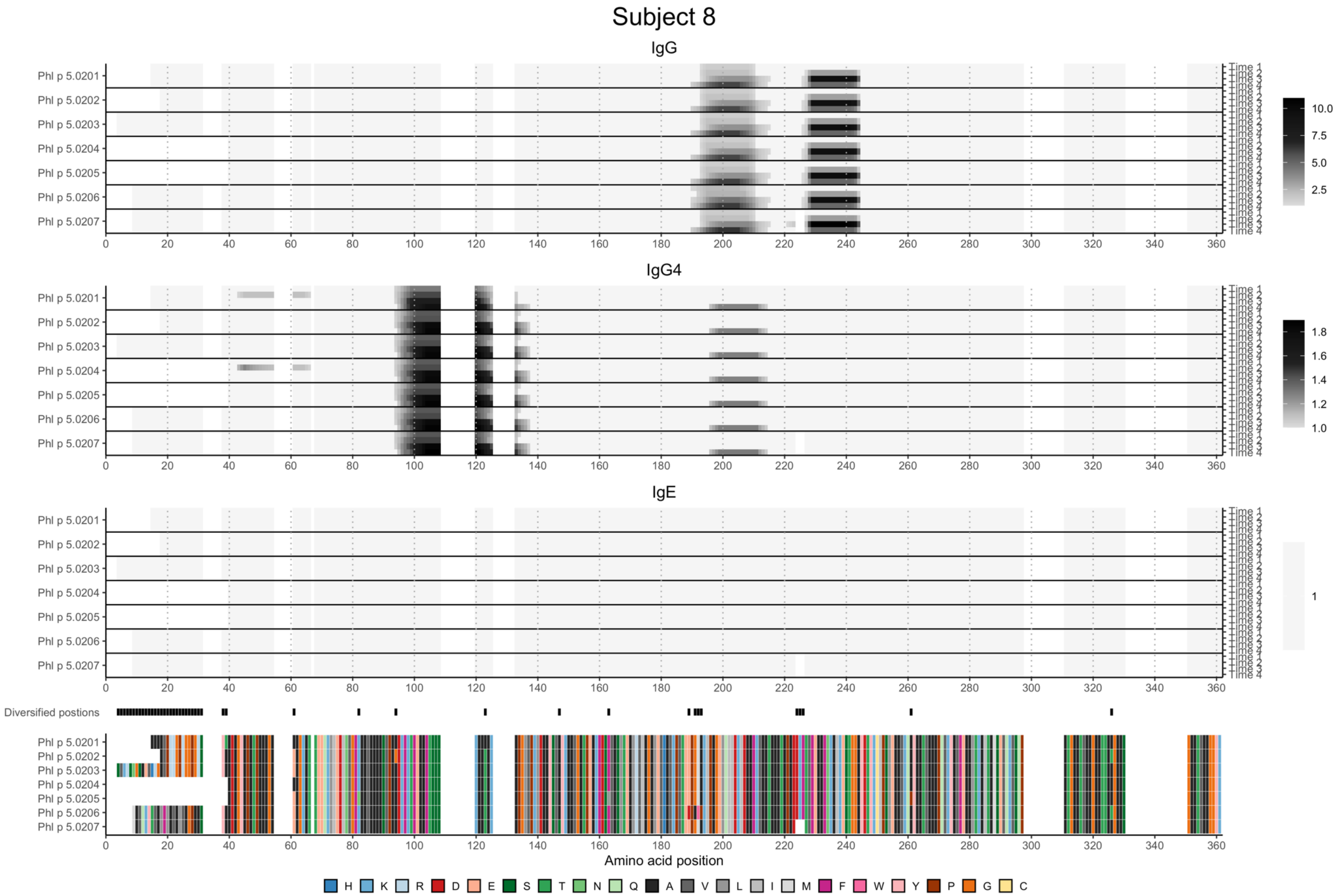

Subject 8
IgG
IgG4
IgE
Phl p 5.0201
Phl p 5.0202
Phl p 5.0203
Phl p 5.0204
Phl p 5.0205
Phl p 5.0206
Phl p 5.0207
Time 1
Time 2
Time 3
Time 4
10.0
7.5
5.0
2.5
1.8
1.6
1.4
1.2
1.0
1
Diversified postions
Amino acid position
0 20 40 60 80 100 120 140 160 180 200 220 240 260 280 300 320 340 360
H K R D E S T N Q A V L I M F W Y P G C

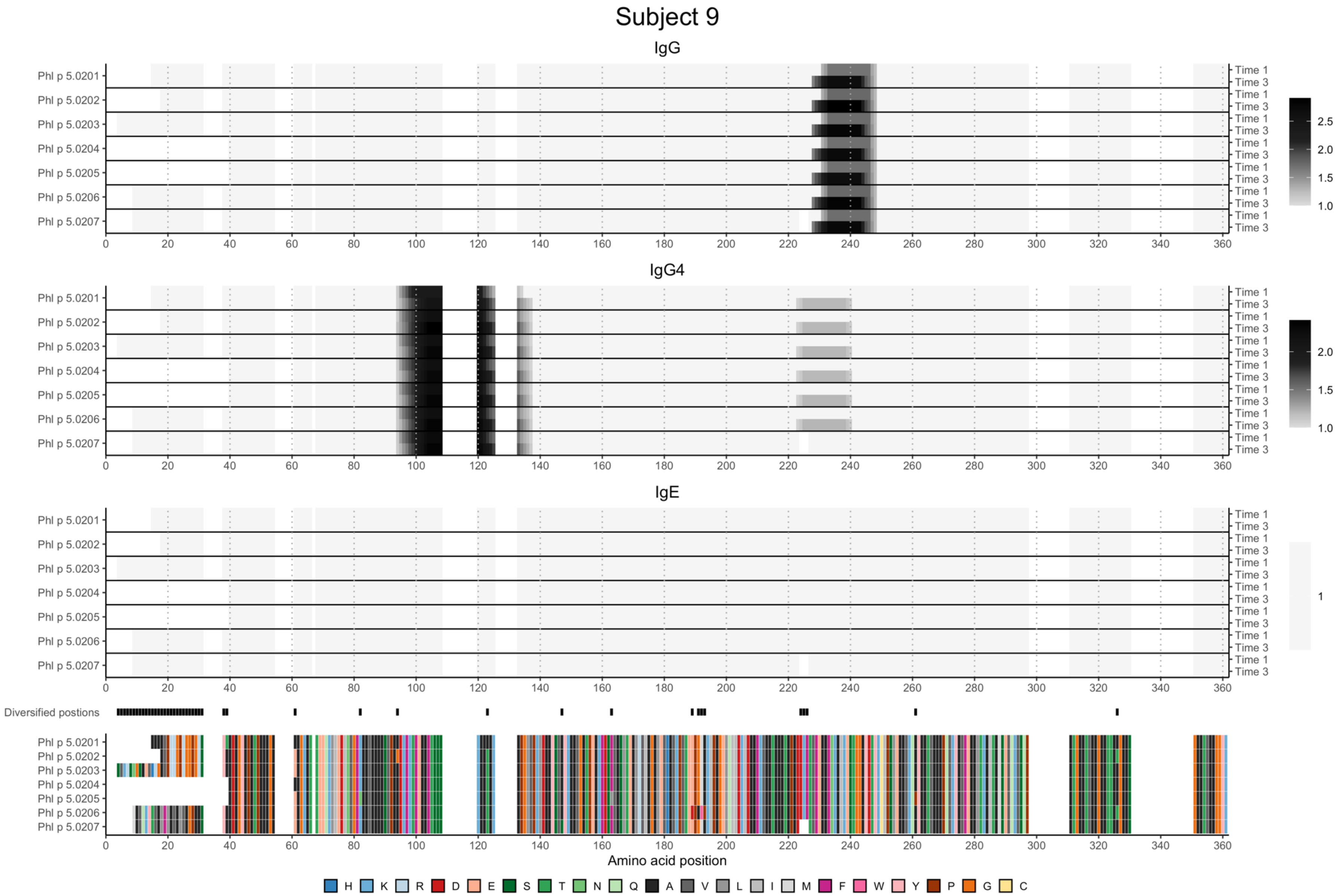
Subject 9
IgG
IgG4
IgE
Phl p 5.0201
Phl p 5.0202
Phl p 5.0203
Phl p 5.0204
Phl p 5.0205
Phl p 5.0206
Phl p 5.0207
Time 1
Time 3
Diversified postions
Amino acid position
H K R D E S T N Q A V L I M F W Y P G C

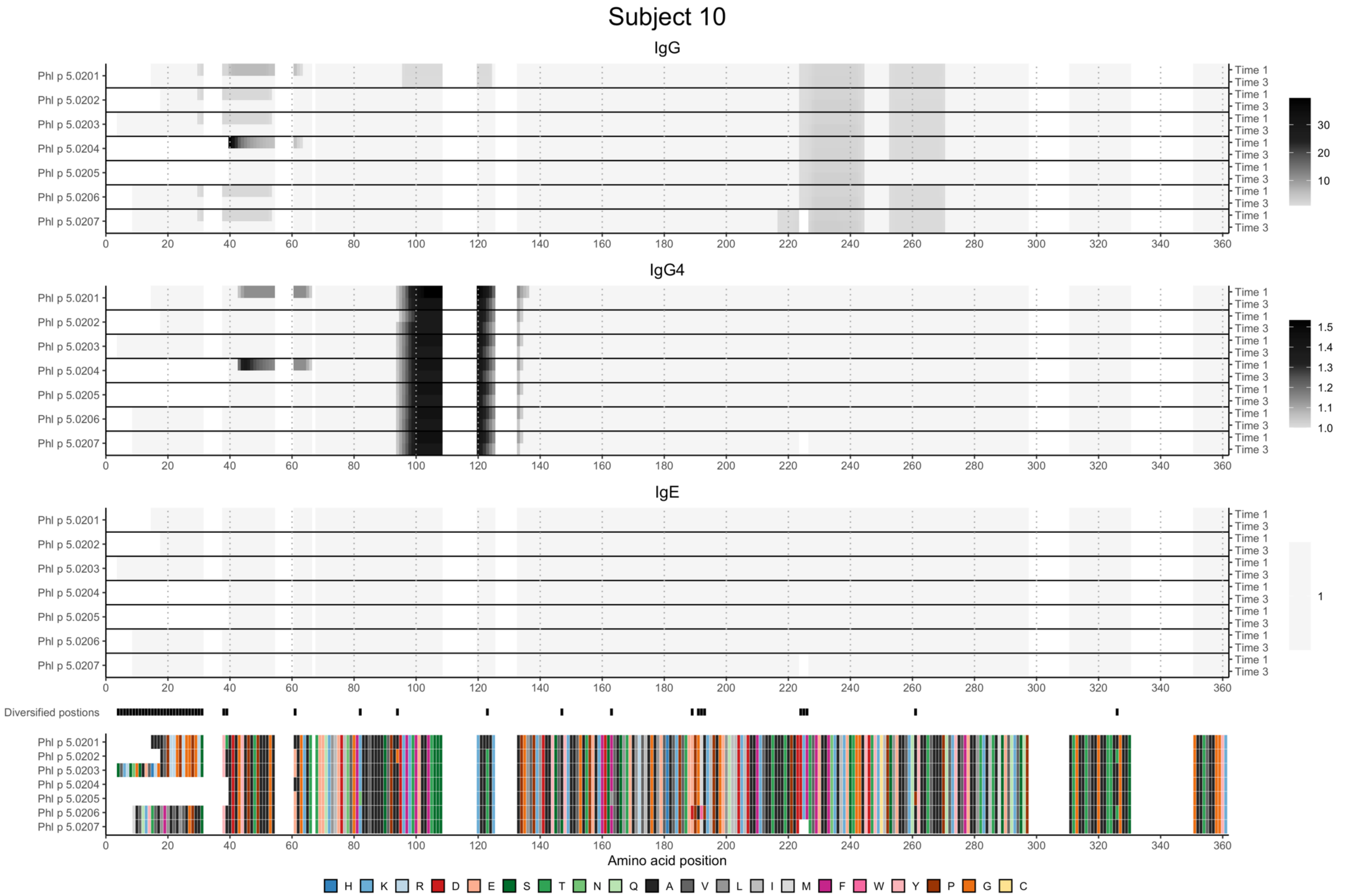

Subject 10
IgG
IgG4
IgE
Phl p 5.0201
Phl p 5.0202
Phl p 5.0203
Phl p 5.0204
Phl p 5.0205
Phl p 5.0206
Phl p 5.0207
Time 1
Time 3
Diversified postions
Amino acid position
H K R D E S T N Q A V L I M F W Y P G C

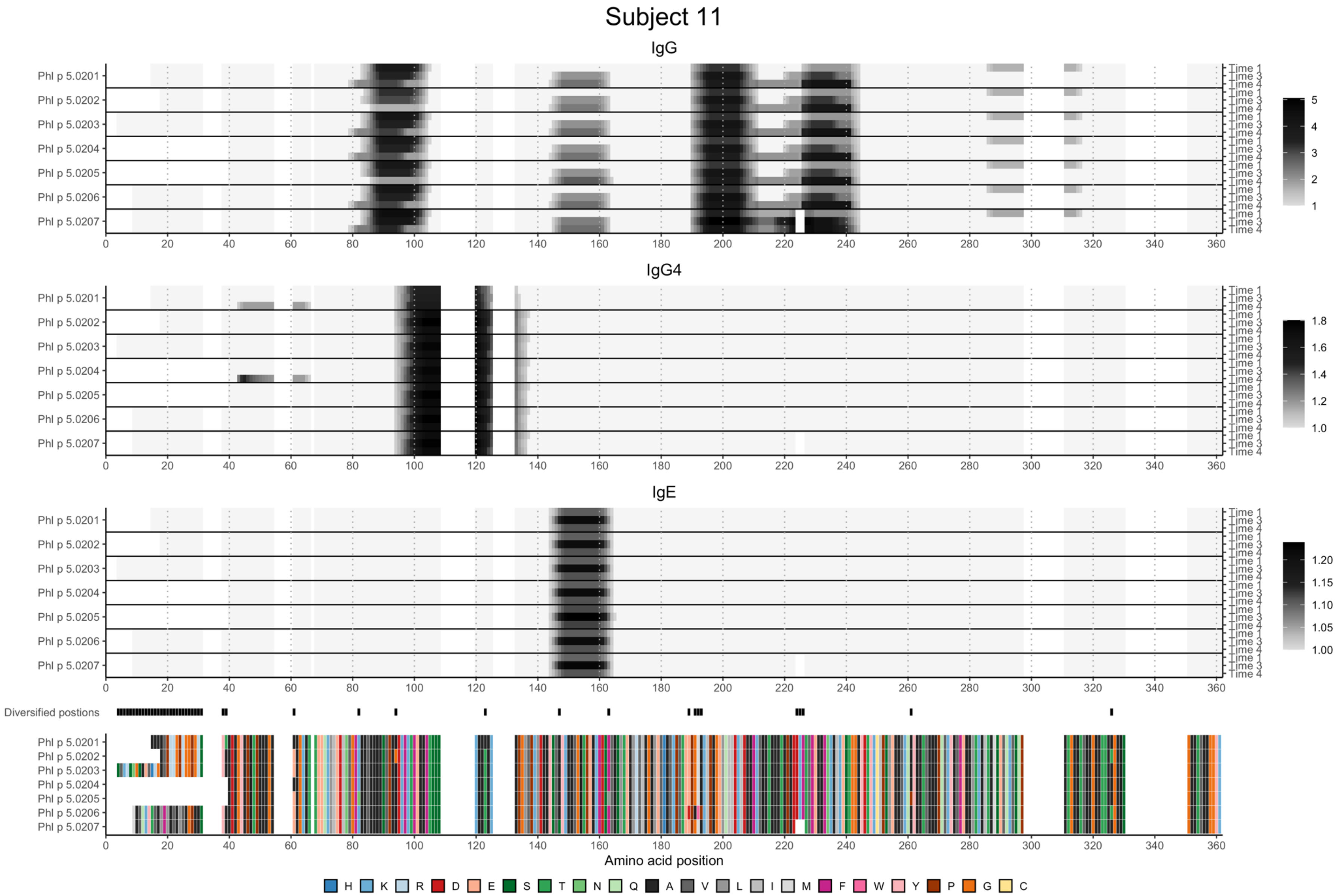

Subject 11
IgG
IgG4
IgE
Phl p 5.0201
Phl p 5.0202
Phl p 5.0203
Phl p 5.0204
Phl p 5.0205
Phl p 5.0206
Phl p 5.0207
Time 1
Time 3
Time 4
Diversified postions
Amino acid position
H K R D E S T N Q A V L I M F W Y P G C

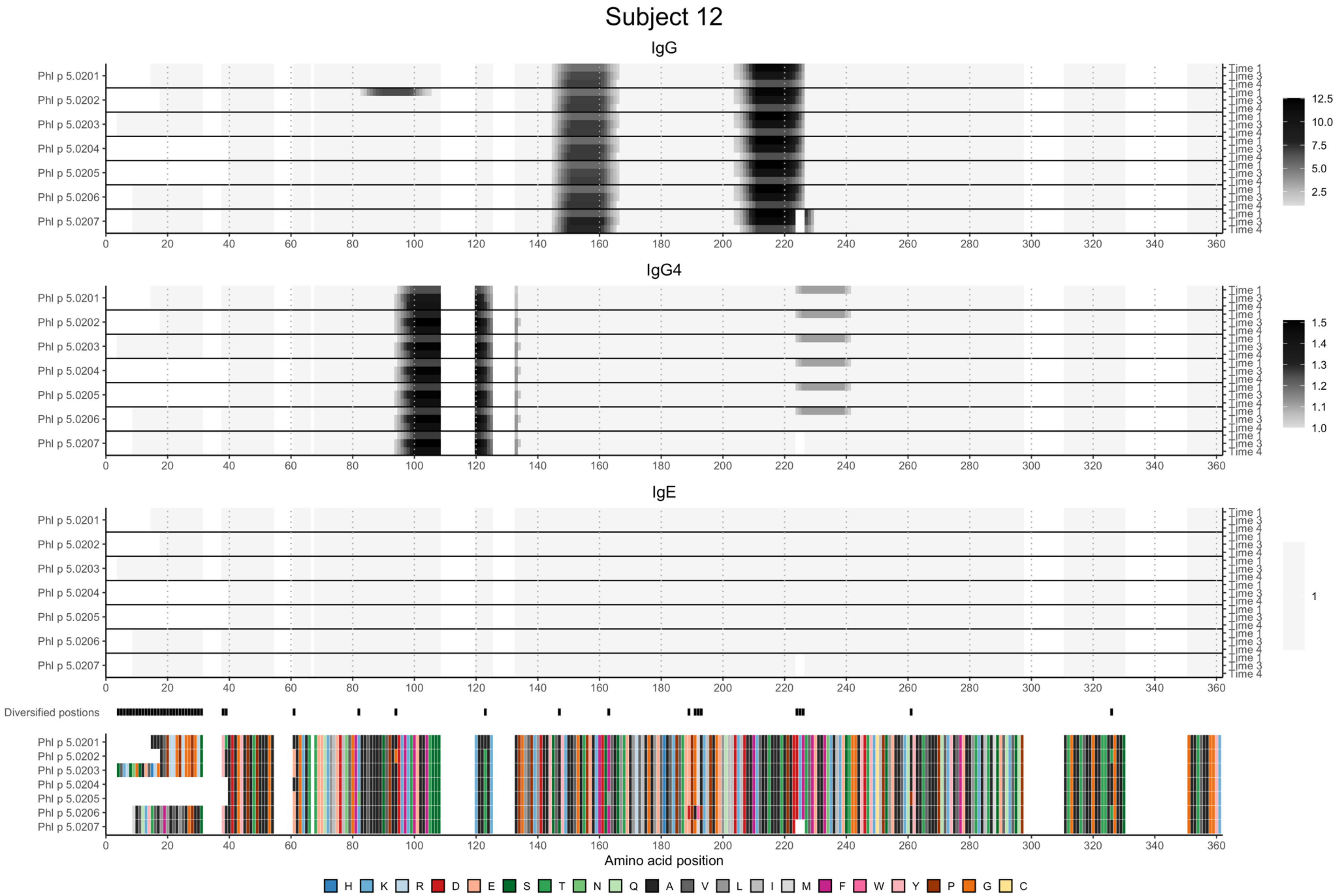

Subject 12
IgG
IgG4
IgE
Phl p 5.0201
Phl p 5.0202
Phl p 5.0203
Phl p 5.0204
Phl p 5.0205
Phl p 5.0206
Phl p 5.0207
Time 1
Time 3
Time 4
12.5
10.0
7.5
5.0
2.5
1.5
1.4
1.3
1.2
1.1
1.0
1
Diversified postions
Amino acid position
H K R D E S T N Q A V L I M F W Y P G C

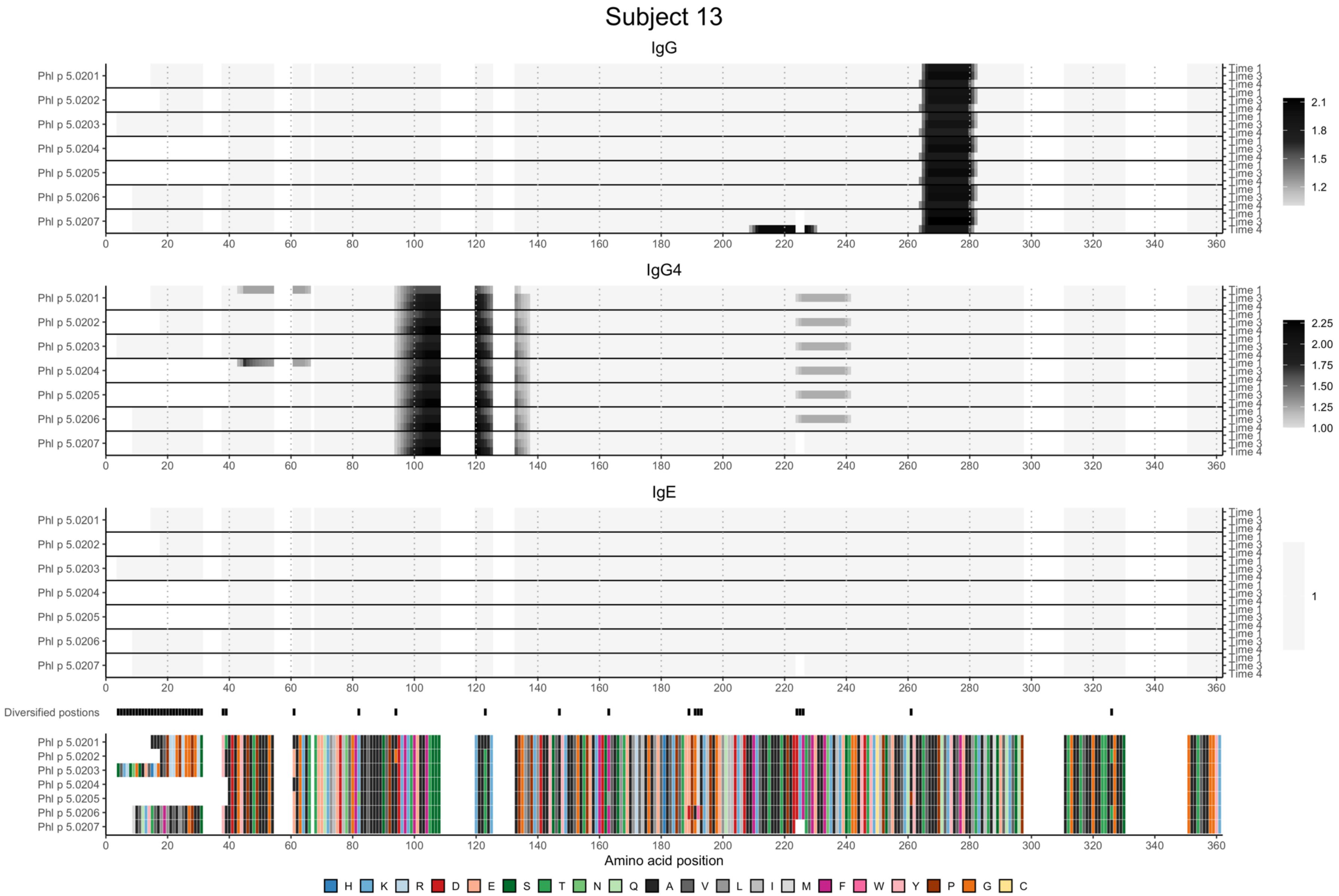

Subject 13
IgG
IgG4
IgE
Phl p 5.0201
Phl p 5.0202
Phl p 5.0203
Phl p 5.0204
Phl p 5.0205
Phl p 5.0206
Phl p 5.0207
Time 1
Time 3
Time 4
Diversified postions
Amino acid position
H K R D E S T N Q A V L I M F W Y P G C

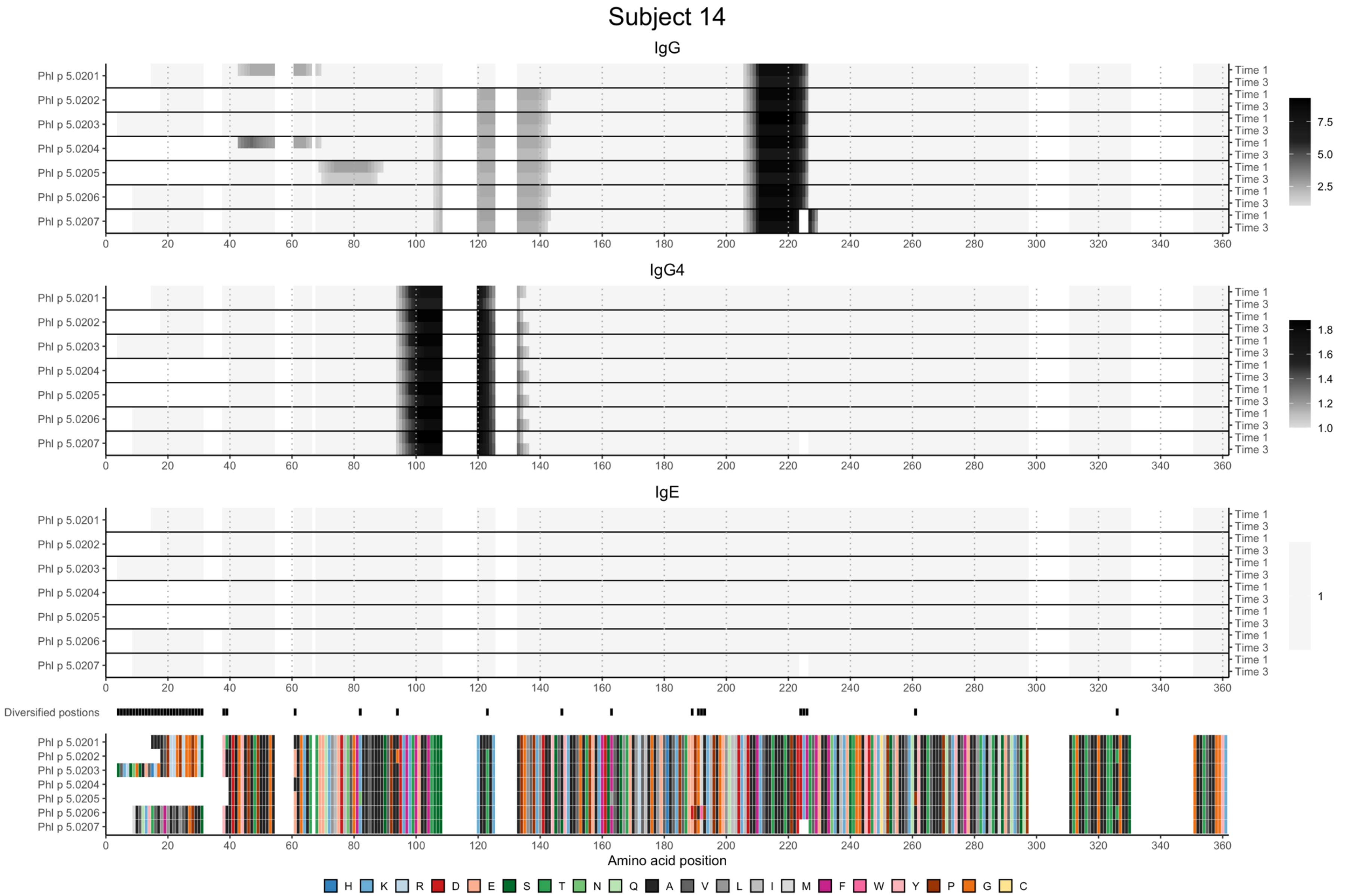
Subject 14
IgG
IgG4
IgE
Phl p 5.0201
Phl p 5.0202
Phl p 5.0203
Phl p 5.0204
Phl p 5.0205
Phl p 5.0206
Phl p 5.0207
Time 1
Time 3
7.5
5.0
2.5
1.8
1.6
1.4
1.2
1.0
1
Diversified postions
Amino acid position
0 20 40 60 80 100 120 140 160 180 200 220 240 260 280 300 320 340 360
H K R D E S T N Q A V L I M F W Y P G C

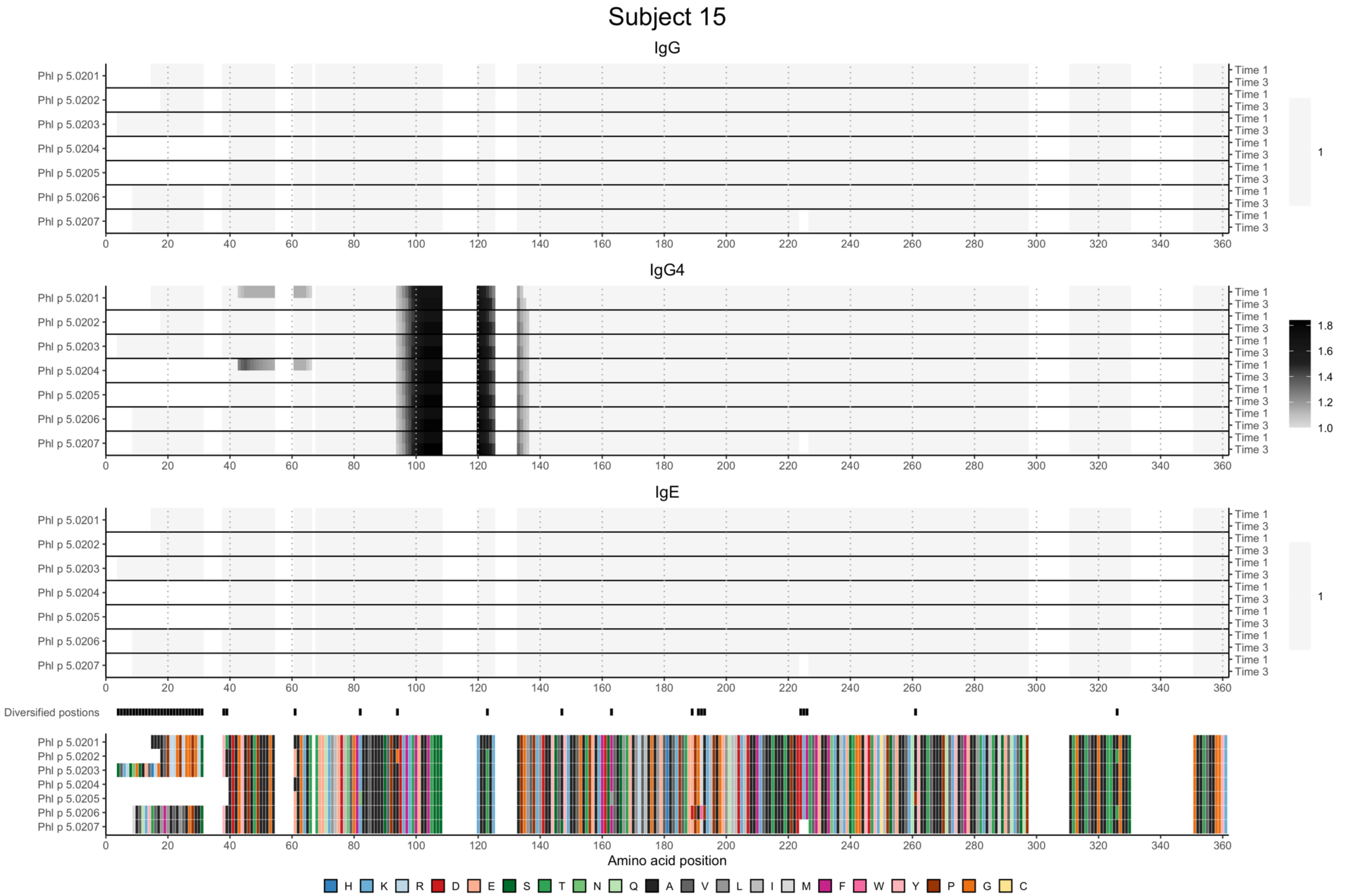
Subject 15
IgG
IgG4
IgE
Phl p 5.0201
Phl p 5.0202
Phl p 5.0203
Phl p 5.0204
Phl p 5.0205
Phl p 5.0206
Phl p 5.0207
Time 1
Time 3
1
1.8
1.6
1.4
1.2
1.0
0 20 40 60 80 100 120 140 160 180 200 220 240 260 280 300 320 340 360
Diversified postions
Amino acid position
H K R D E S T N Q A V L I M F W Y P G C

Supplementary Figure 11. Heatmap visualisation of IgG, IgG4, and IgE recognition of linear epitopes for Phl 5.02 allergen variants. Peptide signals have been used to estimate values on an amino acid level, as described. Amino acid values of 1 represents background, *i.e.*, amino acids that are not part of any reactive peptides. Epitope C have been highlighted in red. Sequence alignment of Phl p 5.02 allergen variants is also presented, with diversified residues marked above the alignment.
