# Supplementary material for: Linear Epitope Binding Patterns of Grass Pollen-Specific Antibodies in Allergy and in Response to Allergen-Specific Immunotherapy
Source: Front Allergy. 2022 Mar 31;3:859126. doi: 10.3389/falgy.2022.859126 (PMC9234942; doi:10.3389/falgy.2022.859126)
Supplement: Supplementary file 2 [file Data_Sheet_2.ZIP › Supplementary Figure 12.pdf]

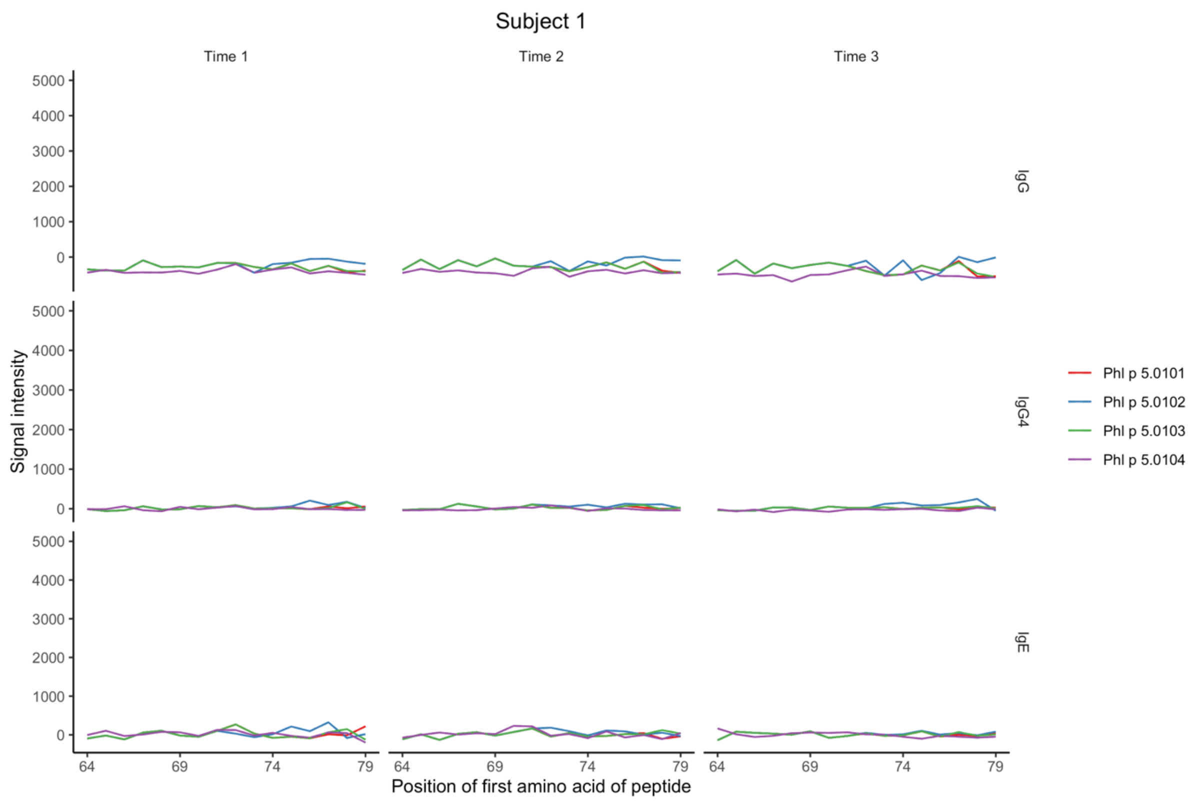
Subject 1
Time 1
Time 2
Time 3
IgG
IgG4
IgE
Signal intensity
Position of first amino acid of peptide
Phl p 5.0101
Phl p 5.0102
Phl p 5.0103
Phl p 5.0104

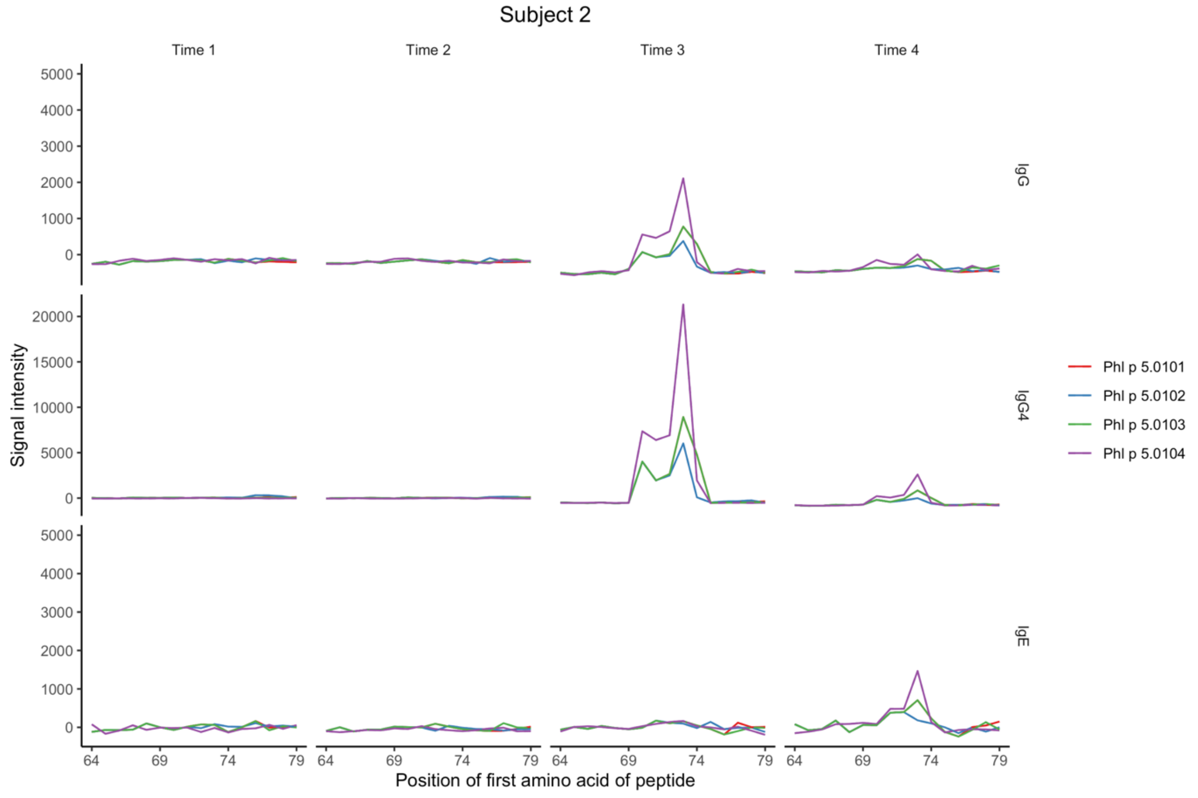
Subject 2
Time 1
Time 2
Time 3
Time 4
IgG
IgG4
IgE
Signal intensity
Position of first amino acid of peptide
Phl p 5.0101
Phl p 5.0102
Phl p 5.0103
Phl p 5.0104

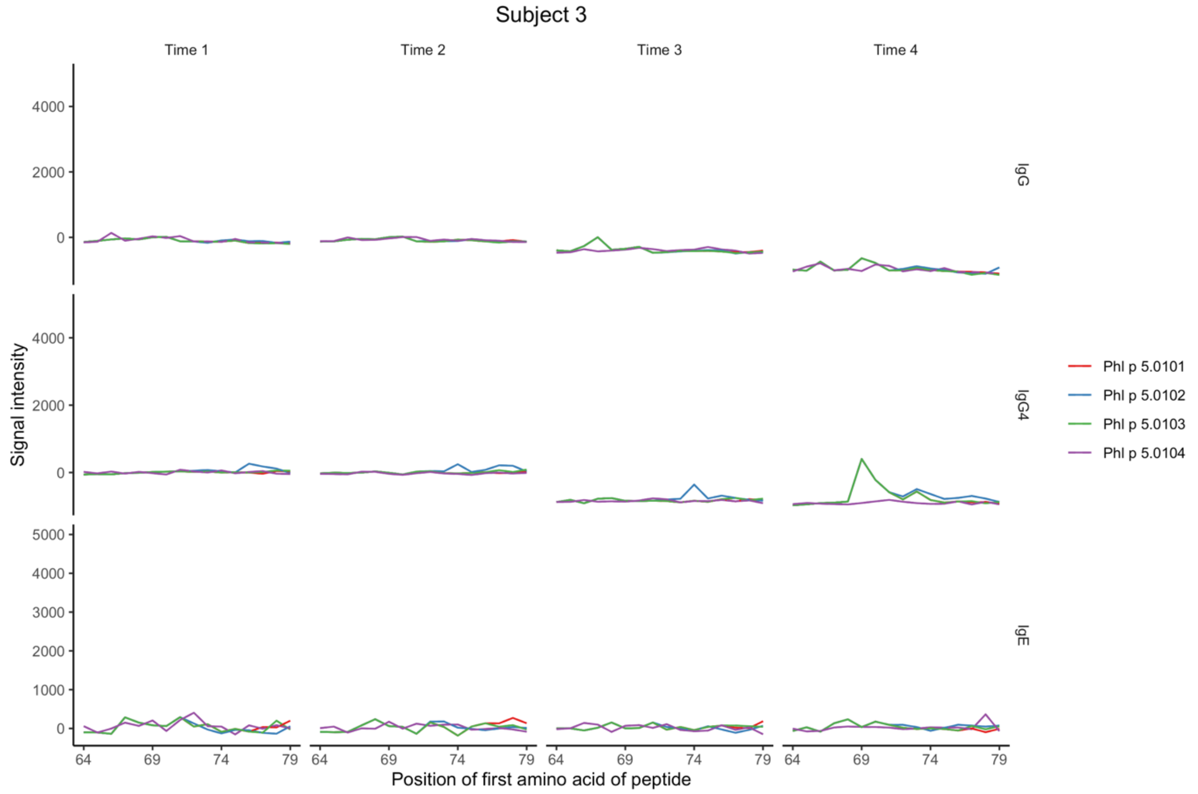

Subject 3
Time 1
Time 2
Time 3
Time 4
IgG
IgG4
IgE
Signal intensity
Position of first amino acid of peptide
Phl p 5.0101
Phl p 5.0102
Phl p 5.0103
Phl p 5.0104

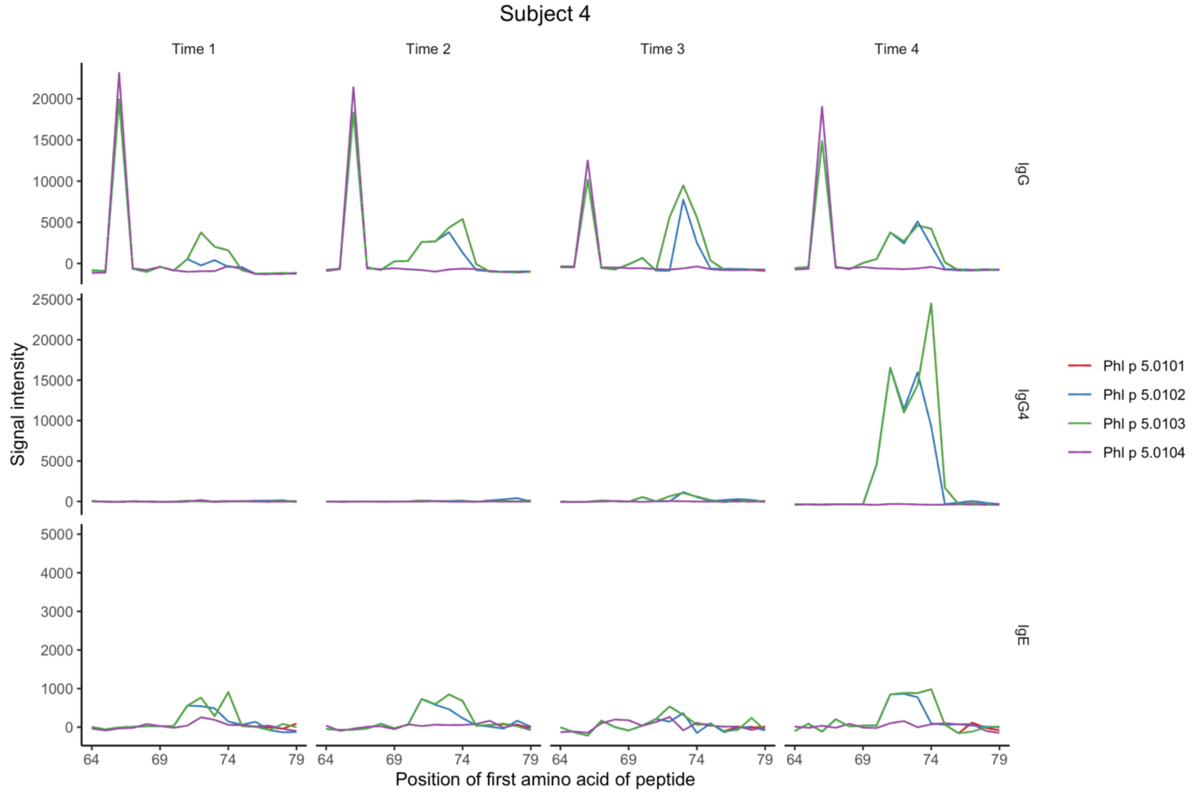

Subject 4
Time 1
Time 2
Time 3
Time 4
IgG
IgG4
IgE
Signal intensity
Position of first amino acid of peptide
Phl p 5.0101
Phl p 5.0102
Phl p 5.0103
Phl p 5.0104

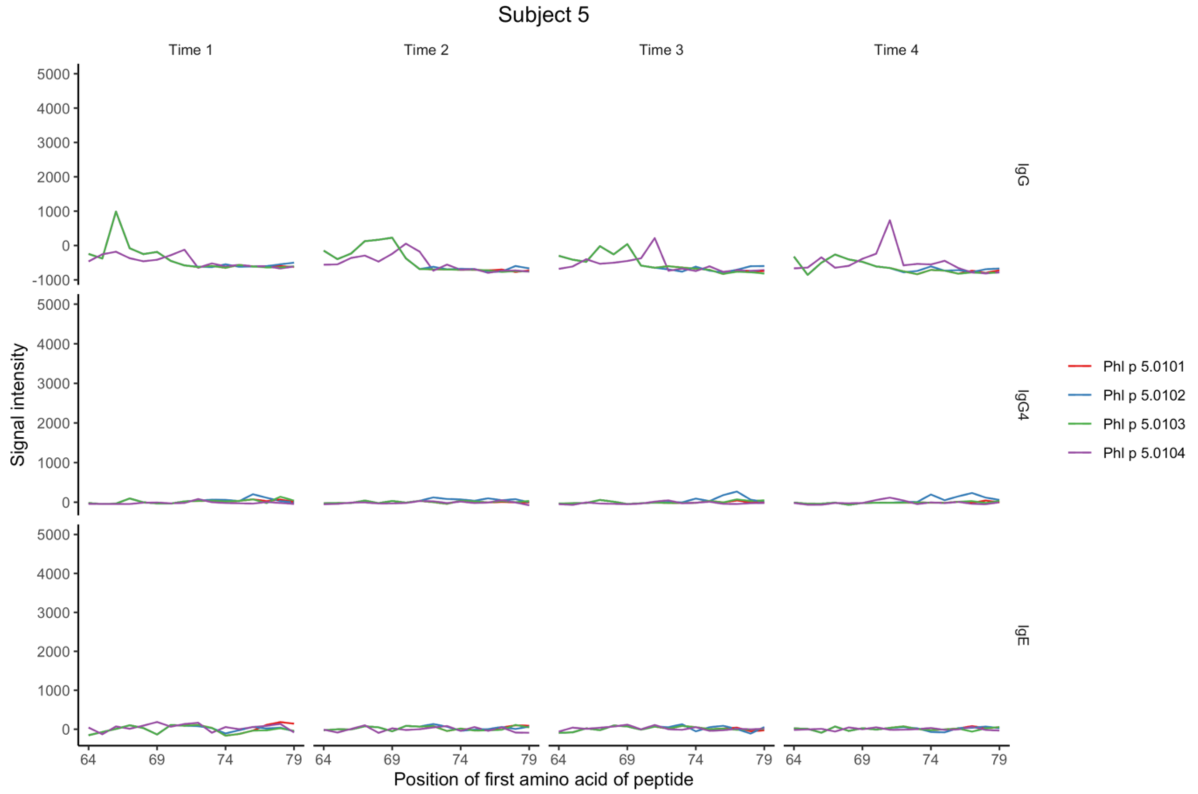
Subject 5
Time 1
Time 2
Time 3
Time 4
IgG
IgG4
IgE
Signal intensity
Position of first amino acid of peptide
Phl p 5.0101
Phl p 5.0102
Phl p 5.0103
Phl p 5.0104

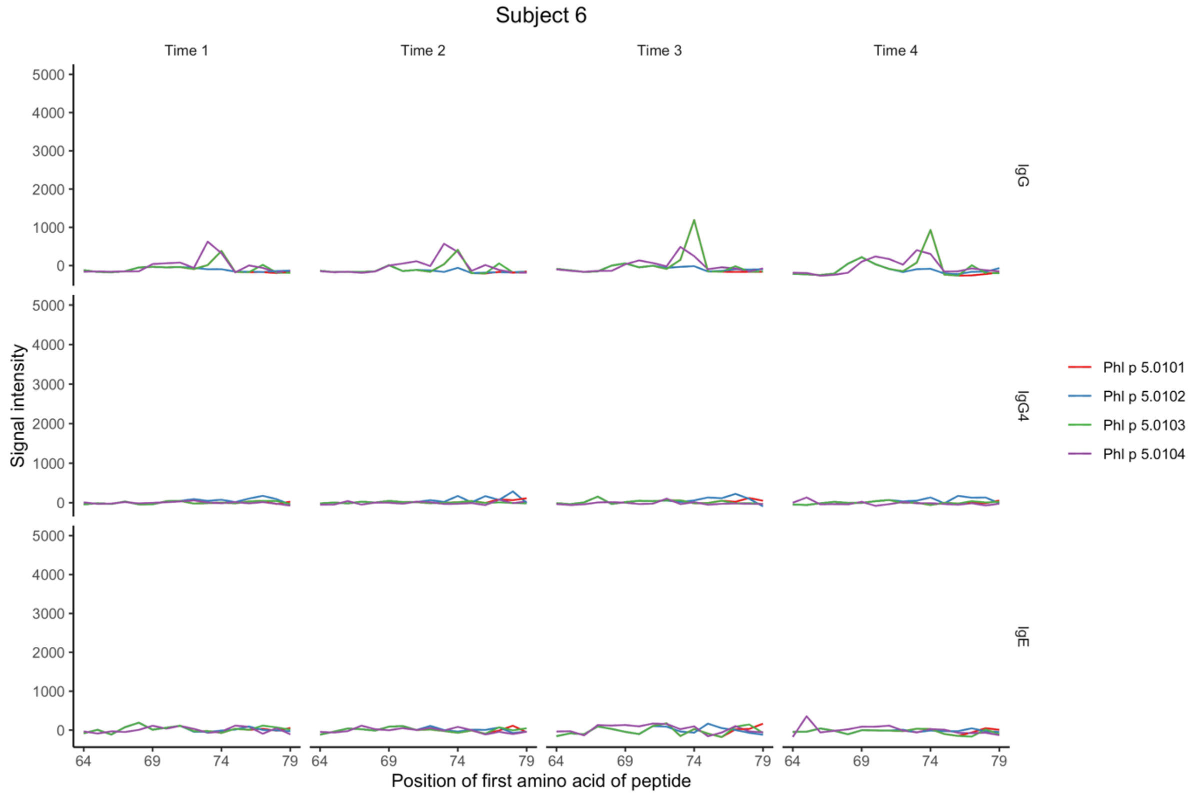
Subject 6
Time 1
Time 2
Time 3
Time 4
IgG
IgG4
IgE
Signal intensity
Position of first amino acid of peptide
Phl p 5.0101
Phl p 5.0102
Phl p 5.0103
Phl p 5.0104

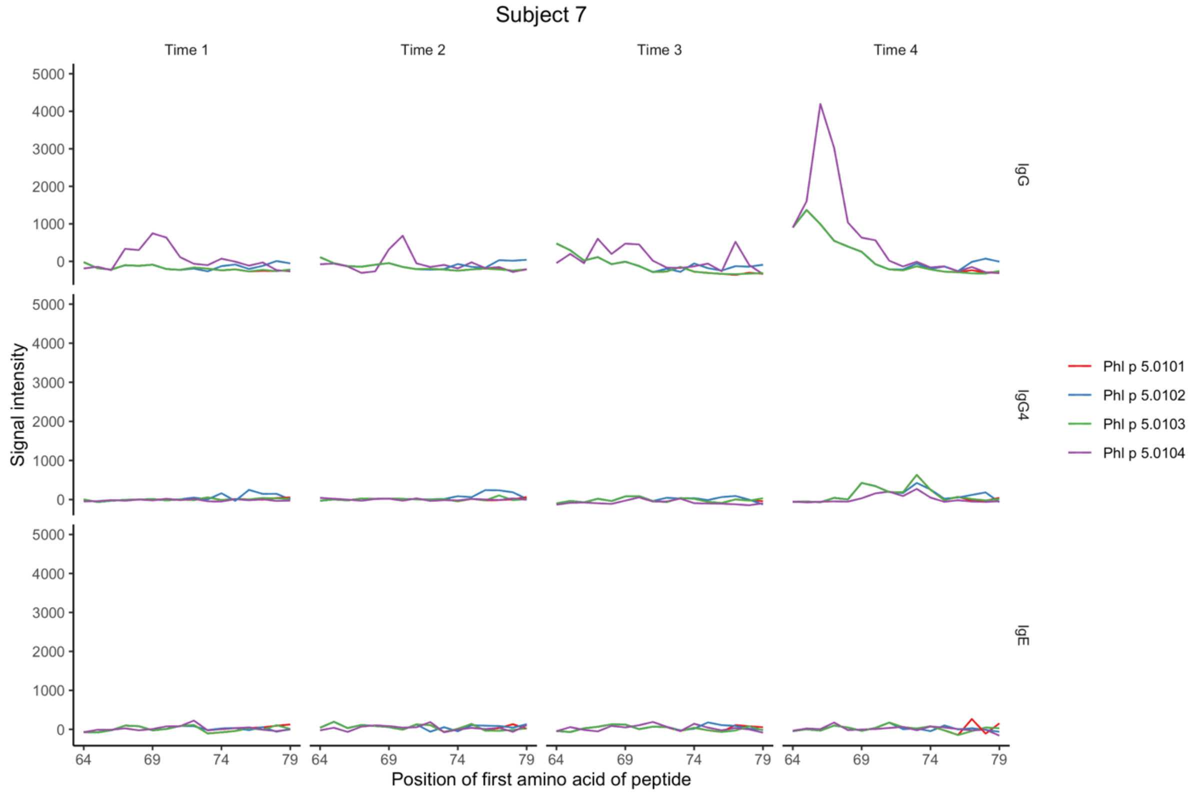
Subject 7
Time 1
Time 2
Time 3
Time 4
IgG
IgG4
IgE
Signal intensity
0
1000
2000
3000
4000
5000
64
69
74
79
Position of first amino acid of peptide
Phl p 5.0101
Phl p 5.0102
Phl p 5.0103
Phl p 5.0104

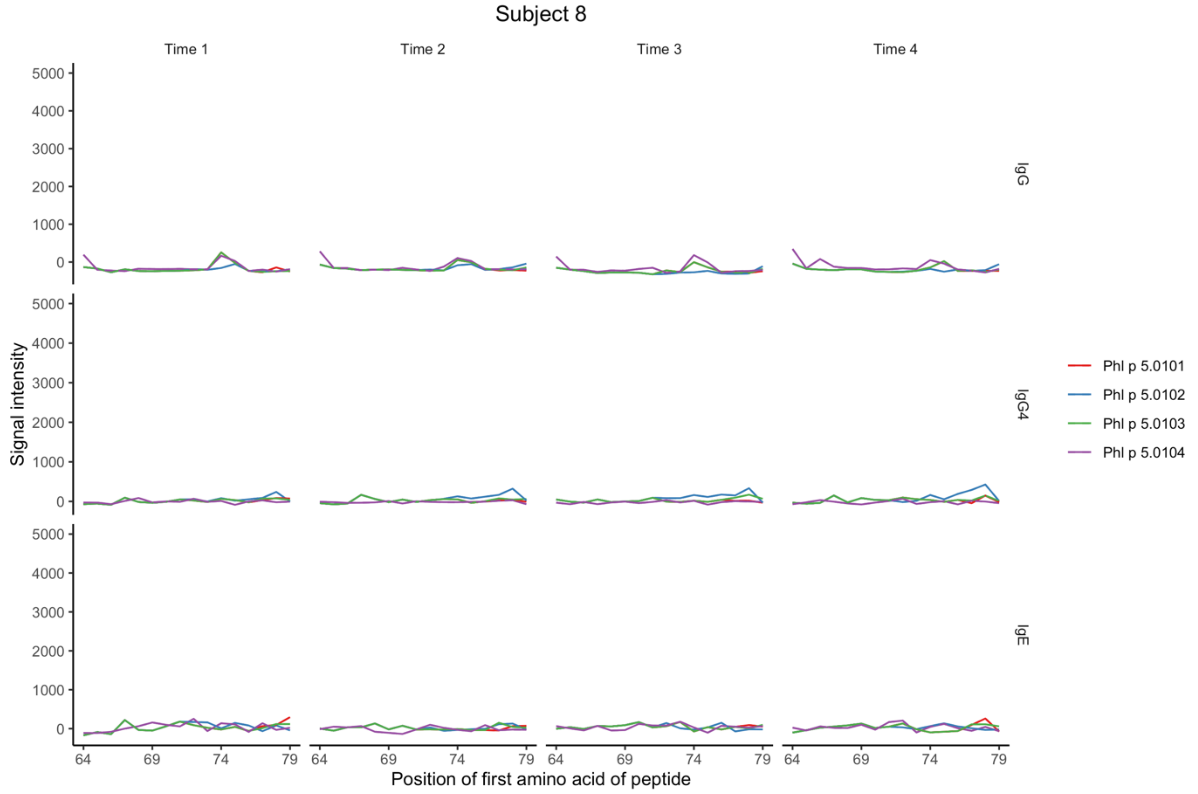
Subject 8
Time 1
Time 2
Time 3
Time 4
IgG
IgG4
IgE
Signal intensity
0
1000
2000
3000
4000
5000
64
69
74
79
Position of first amino acid of peptide
Phl p 5.0101
Phl p 5.0102
Phl p 5.0103
Phl p 5.0104

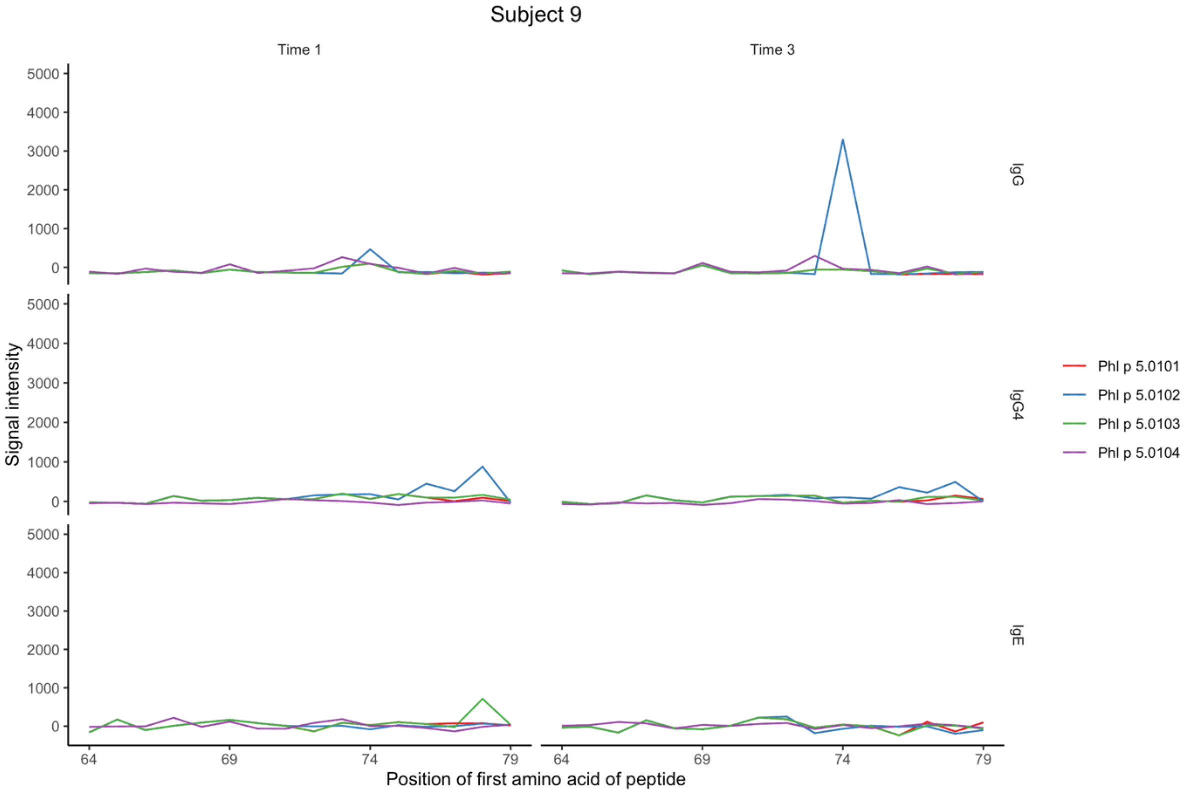
Subject 9
Time 1
Time 3
IgG
IgG4
IgE
Signal intensity
Position of first amino acid of peptide
Phl p 5.0101
Phl p 5.0102
Phl p 5.0103
Phl p 5.0104

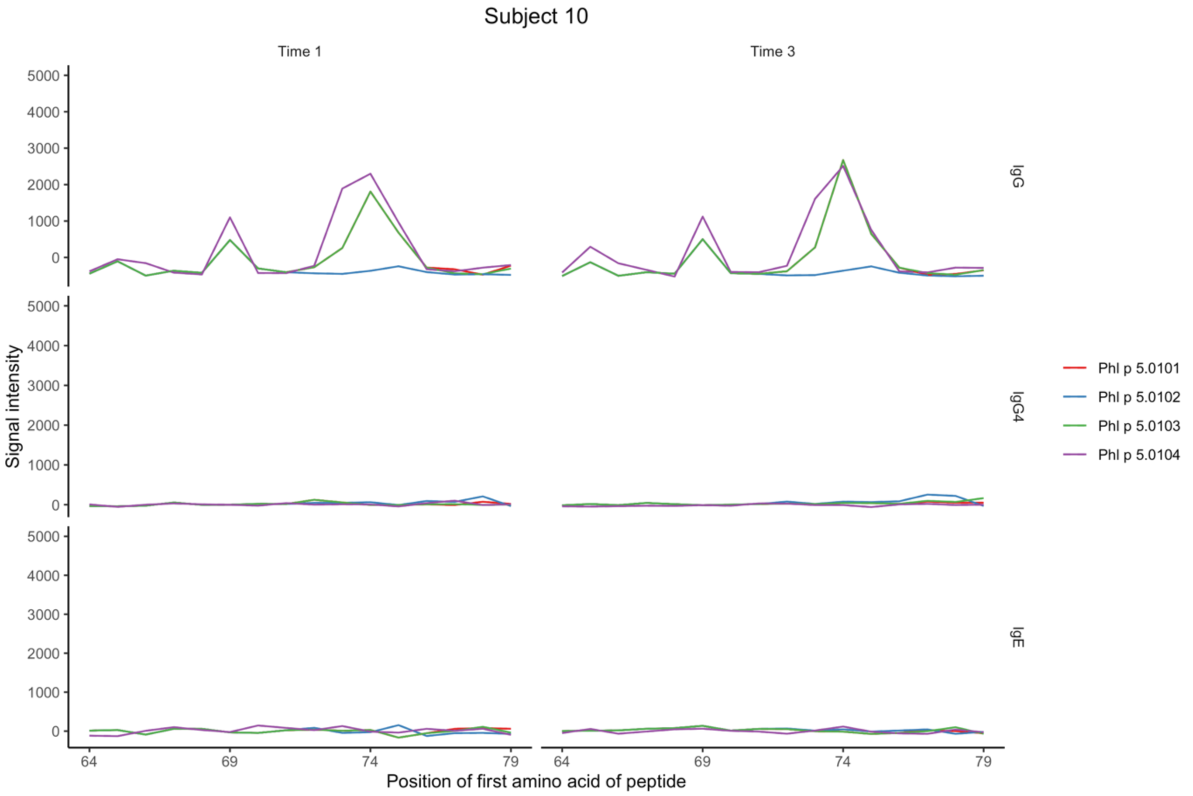
Subject 10
Time 1
Time 3
IgG
IgG4
IgE
Signal intensity
Position of first amino acid of peptide
Phl p 5.0101
Phl p 5.0102
Phl p 5.0103
Phl p 5.0104

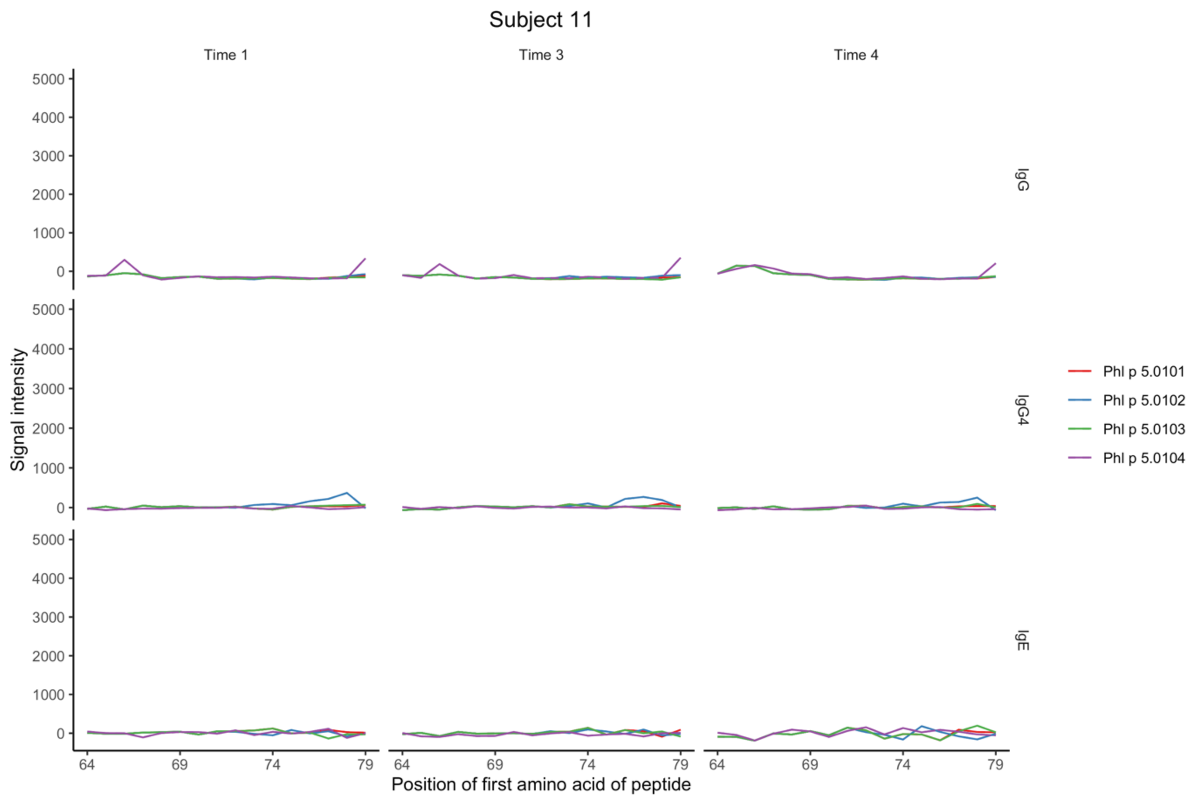
Subject 11
Time 1
Time 3
Time 4
Signal intensity
IgG
IgG4
IgE
5000
4000
3000
2000
1000
0
64
69
74
79
Position of first amino acid of peptide
Phl p 5.0101
Phl p 5.0102
Phl p 5.0103
Phl p 5.0104

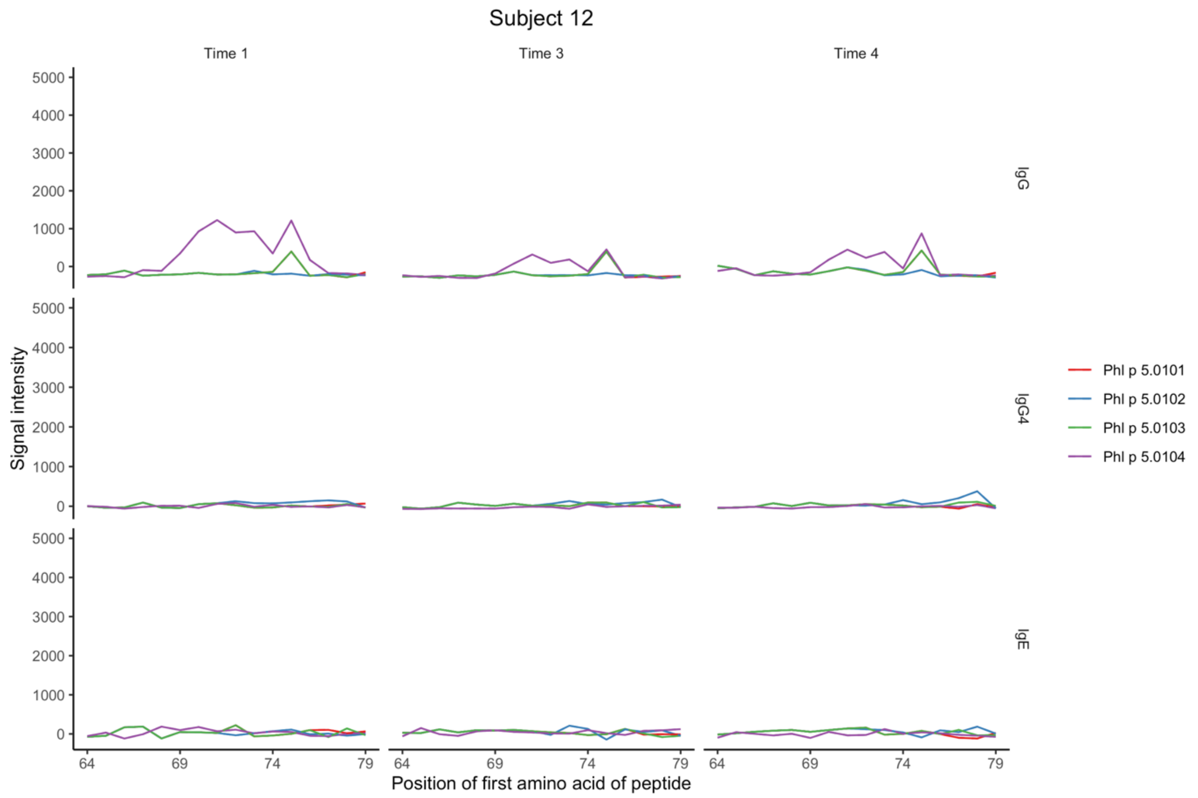
Subject 12
Time 1
Time 3
Time 4
Signal intensity
IgG
IgG4
IgE
5000
4000
3000
2000
1000
0
64
69
74
79
Position of first amino acid of peptide
Phl p 5.0101
Phl p 5.0102
Phl p 5.0103
Phl p 5.0104

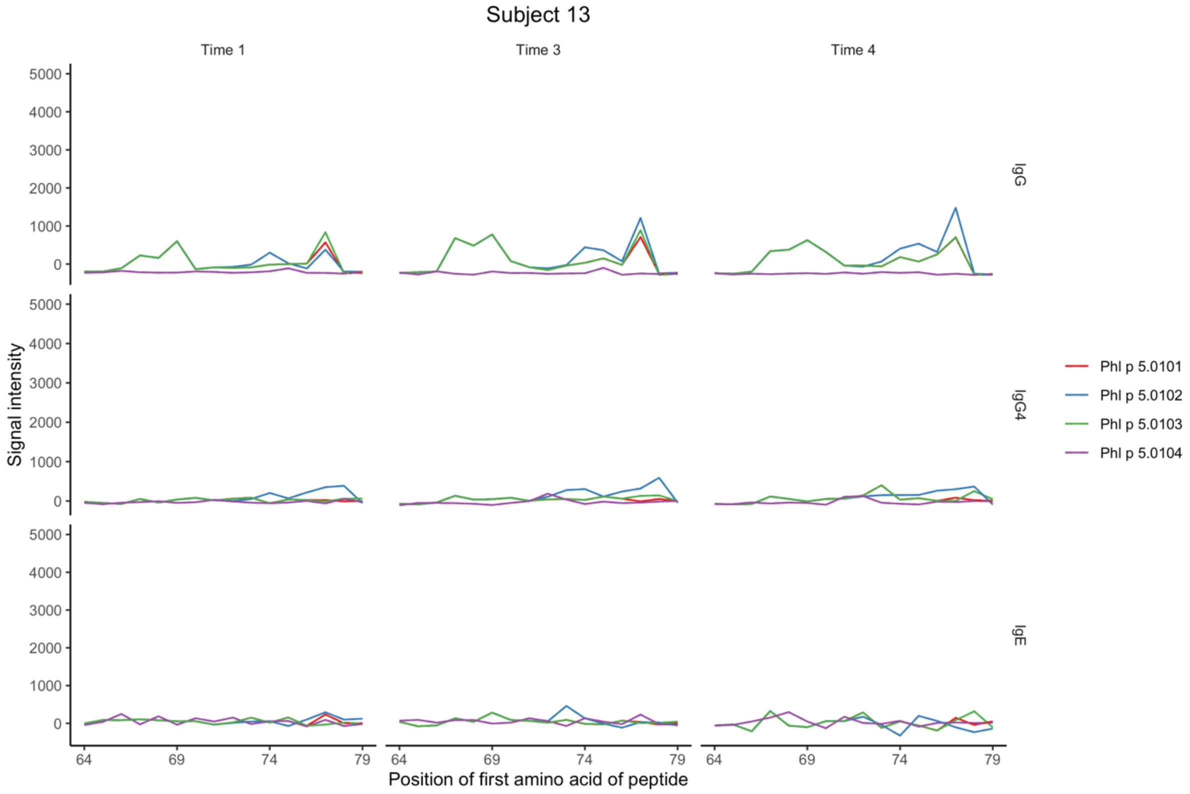
Subject 13
Time 1
Time 3
Time 4
5000
4000
3000
2000
1000
0
IgG
IgG4
IgE
Signal intensity
64
69
74
79
Position of first amino acid of peptide
Phl p 5.0101
Phl p 5.0102
Phl p 5.0103
Phl p 5.0104

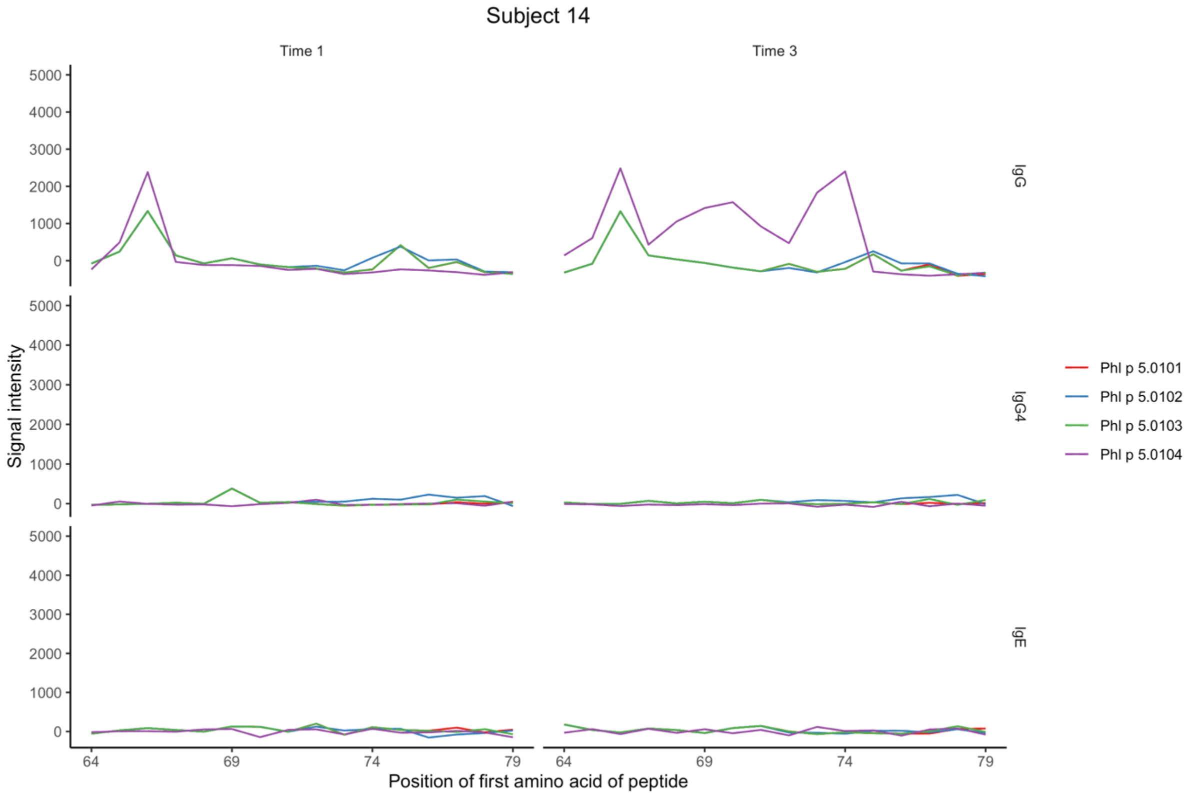
Subject 14
Time 1
Time 3
5000
4000
3000
2000
1000
0
IgG
IgG4
IgE
Signal intensity
64
69
74
79
Position of first amino acid of peptide
Phl p 5.0101
Phl p 5.0102
Phl p 5.0103
Phl p 5.0104

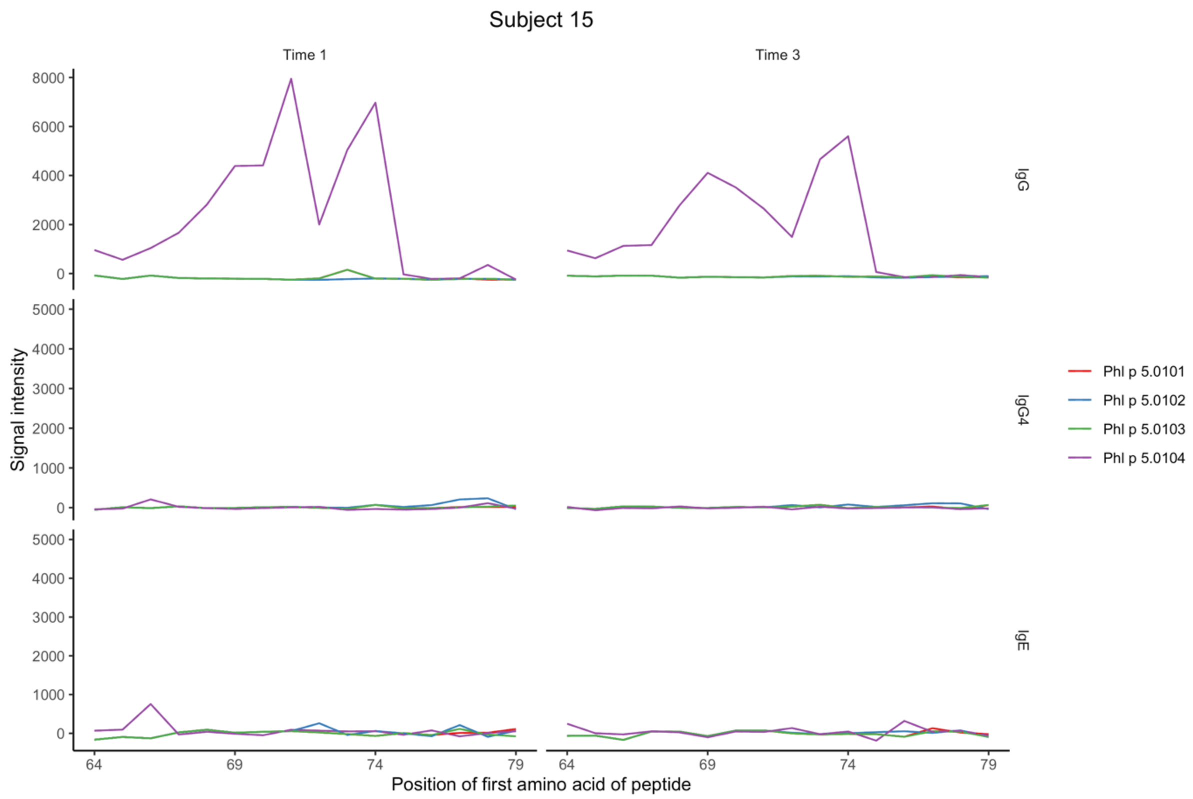

Supplementary Figure 12. IgG, IgG4, and IgE signal intensities for epitope C, *i.e.*, peptide 64-79 of Phl p 5.01 allergen variants. These peptides all stretch over residue 79, where they express either an alanine (Phl p 5.0101-5.0103 and Phl p 5.0109) or an aspartic acid (Phl p 5.0104-5.0108) amino acid. Variant Phl p 5.0109 is identical to Phl p 5.0103 in this region, and is therefore not included in the plots. Similarly, 5.0105-5.0108 have been excluded, as they are identical to 5.0104 in this region. Additionally, Phl p 5.0101 is identical to Phl p 5.0102 in peptides starting before position 73 and identical to Phl p 5.0102 in peptides starting before position 77. Hence, the signal of Phl p 5.0101 is only visible for peptide 78-79. Samples had been collected at AIT treatment initiation (time 1), and 8 weeks (time 2), 1 year (time 3), and 3 years later (time 4).
